# A molecular-logic gate for COX-2 and NAT based on conformational and structural changes: visualizing the progression of liver disease†
†Electronic supplementary information (ESI) available. See DOI: 10.1039/d0sc00574f


**DOI:** 10.1039/d0sc00574f

**Published:** 2020-05-25

**Authors:** Yuehua Chen, Yuzhu Wang, Yonggang Yang, Yuhuan Li, Yafu Wang, Ge Wang, Tony D. James, Xiaopeng Xuan, Hua Zhang, Yufang Liu

**Affiliations:** a Henan Key Laboratory of Green Chemical Media and Reactions , Ministry of Education , Henan Key Laboratory of Organic Functional Molecules and Drug Innovation , School of Chemistry and Chemical Engineering , School of Physics , Henan Normal University , Xinxiang 453007 , P. R. China . Email: zhanghua1106@163.com; b Department of Hepatobiliary and Pancreatic Surgery , Henan Provincial People's Hospital , Zhengzhou University People's Hospital , Henan University People's Hospital , Zhengzhou , Henan 450003 , P. R. China; c Department of Chemistry , University of Bath , Bath , BA2 7AY , UK . Email: t.d.james@bath.ac.uk; d Xinxiang Medical University , Xinxiang 453000 , P. R. China

## Abstract

Lighting up the relevant lesion boundaries during operations is vital for guiding the effective resection of hepatopathic tissue. We envisioned that molecular-logic gates, which are known for their excellent digital correlation between input and output signals, could be used to facilitate differential visualization of lesion boundaries. Herein, a series of flexible molecules, naphthalene imide-indole derivatives (**IAN**) were prepared and evaluated as molecular-logic gates. The input and output signals of the **IAN** derivatives were successfully used to highlight different hepatopathic regions in order to facilitate boundary differentiation. The **IAN** derivatives produce different signals due to collaborative changes in the conformation and structure. The hepatopathy-related enzymes (COX-2 and NAT) were used to induce conformational and structural changes in **IAN** derivatives. Based on these enzyme induced synergistic effects, **IAN** can sensitively emit different coloured signals such as green, cyan and blue (output signals) as a function of the different input signals, *i.e.* the different activity of COX-2 and NAT in solution and living cells. Significantly, the **IAN** derivatives were successfully used to distinguish the boundaries of hepatopathic lesions in tissues after spraying with **IAN** derivatives (mild cirrhosis, severe cirrhosis, in addition to early and late hepatocellular carcinoma) under a hand held lamp at 365 nm by naked eye.

## Abstract 2

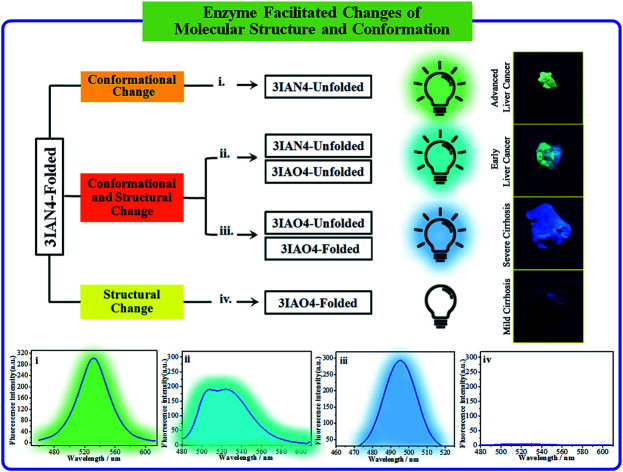
Lighting up the relevant lesion boundaries during operations is vital for guiding the effective resection of hepatopathic tissue.

## Introduction

Hepatoma can evolve from the severe deterioration of hepatitis and cirrhosis, and results in a high death rate.^1^ Surgery is the first effective choice for the treatment of hepatopathy including cirrhosis and hepatoma. The accurate monitoring of the boundary between cirrhosis and hepatoma using appropriate signals is the key to the success of surgery.^2^,^3^ To obtain excellent differentiated signals, previous research has used many different biological enzymes, because they show abnormal and different expression activities, during the process of hepatopathic deterioration.^4^,^5^ Among them, cyclooxygenase-2 (COX-2) and N-acetyltransferase (NAT) are attractive targets due to their involvement at all stages of the disease from the earliest inflammatory phase to the premalignant and malignant phase. Importantly, COX-2 and NAT show different expression in different diseased liver tissues and the expression sharply changes as liver disease worsens.^6^,^7^ So, the synergistic effect of COX-2 and NAT represents an important molecular event in the progression of liver disease. Using fluorescent signals to monitor the synergistic effects of COX-2 and NAT during the different deterioration stages is helpful to distinguish cirrhosis and hepatopathic tissues.

Based on this strategy, many excellent organic molecules have been designed to monitor biological enzymes involved in the deterioration process of hepatopathy.^8^–^11^ Over the past several years, many researchers have used molecular structure or conformational changes with target enzymes to regulate the ground state and excited state energy with different output signals.^12^–^16^ For example, some novel enzyme inhibitors with high affinity for COX-2 can selectively emit signals as a result of conformational changes.^17^–^19^ In addition, molecules have been developed to recognise NAT through reactions resulting in structural changes.^20^–^22^ Although these molecules display appropriate recognition signals (such as “off–on” or ratiometric signals) to monitor individual components (for example COX-2 or NAT), such outputs are not able to provide sufficient information on the synergistic effect of the two enzymes during the deterioration process of hepatopathy. In order to monitor the synergistic effects of different enzymes, one effective strategy has been to design molecules with multiple recognition elements.^23^–^26^ For example, multiplexed detection of enzymatic activity has been achieved using multiple recognition signals from responsive luminescent probes.^27^ Recently, smart molecular rotors have been developed to emit multiple signals for different biomacromolecules using molecular conformation changes.^28^ Inspired by the above research, we decided to develop a series of novel molecules with logic based outputs in response to COX-2 and NAT.

With this in mind, a series of the flexible organic molecules, naphthalene imide-indole derivatives (**IAN** derivatives) were designed and synthesized in this work, which display collaborative changes in molecular conformation and structure on exposure to COX-2 and NAT. **IAN** derivatives display a conformational change on exposure to COX-2 resulting in emission at 530 nm, in contrast exposure to NAT results in a structural change and emission at 490 nm. Importantly, **IAN** derivatives display good linear response relationships towards increasing activities of COX-2 and NAT. Therefore, the **IAN** derivatives could emit different outputs depending on the activities of COX-2 and NAT. More importantly, such output signals produce clear logic responses for the different stages in liver disease deterioration. Indicating that our molecular-logic **IAN** derivatives could be used to monitor and differentiate cirrhosis and hepatoma, which in turn could potentially be developed into a system capable of distinguishing the boundary of different lesions during surgery.

## Results and discussion

### Design strategy of **IAN** derivatives

With this research, our aim was to develop a chemical visualization method to effectively guide liver lesion resection during surgery. Which, should improve a patient's postoperative recovery and reduce the damage to the physiological organs. Mild lesions of the liver, such as, mild cirrhosis, do not need to be surgically removed. Therefore, in order, to improve signal contrast, the optimum system is where mild lesions of the liver and normal tissue do not produce any signal resulting in enhanced visualization of important lesions borders during surgery. However, for heavy lesions (severe cirrhosis, early liver cancer and advanced liver cancer), different high-contrast visual signals are needed to distinguish them from the mild lesions and healthy tissue. Such specific clinical demand sets a considerable design challenge for the development of efficient imaging agents (molecular-logic gates). We propose that to achieve this target, we need to uncover new mechanisms of action and recognition targets.

**Scheme 1:**
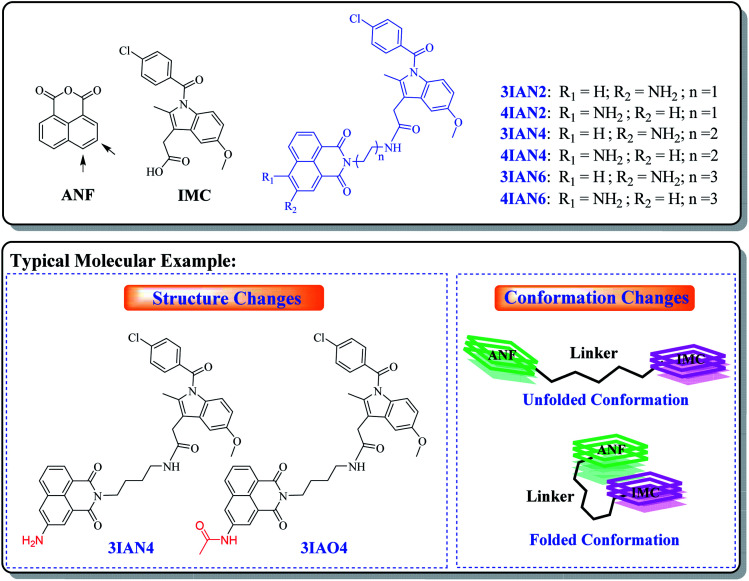
Design of **IAN** derivatives and the typical molecules.

The fundamental chemical problem in designing such molecules is how to regulate changes of their conformation and structure to achieve the desired goal.^29^ Firstly, COX-2 and NAT were selected as the specific targets due to their importance during the different stages in the development of liver disease. Since, both are closely associated with all stages of liver disease and their expression changes with the deterioration of hepatopathy. More importantly, the synergistic effect of COX-2 and NAT can be used to indicate severe deterioration in hepatopathy. With this in mind, a series of flexible organic molecules, naphthalene imide-indole derivatives (**IAN** derivatives) were designed and synthesized, with which to monitor COX-2 and NAT through changes of the molecular structure and conformation. In the molecular design, 1,8-naphthalimide (**ANF**, Scheme 1), was selected as the fluorophore. Since the naphthalimide could easy be substituted by an amino group and other functional groups at the 3- or 4-position.^30^ To facilitate conformational changes of the **IAN** derivatives, different flexible linkers (ethanediamine, butanediamine and hexamethylenediamine, Scheme 1) were introduced into **IAN** derivatives (**3IAN2**, **3IAN4**, **3IAN6**, **4IAN2**, **4IAN4** and **4IAN6**). Then, indomethacin (**IMC**, Scheme 1) as a specific recognition group for COX-2 was added to the other side of the flexible linker, in order to specially interact with the hydrophobic pocket of COX-2.^17^,^18^,^31^ Finally, an amino group was introduced into the 3- or 4-position of **ANF** to interact with NAT, because it can be specifically acetylated by NAT to produce a structural change. Based on this design strategy, we expect that **IAN** derivatives could emit different signals for different liver disease deterioration stages through changes of the conformation and structure. **IAN** derivatives could then be used to mark the boundary between cirrhosis and hepatoma. The synthetic routes of **IAN** derivatives and corresponding intermediates are given in Scheme S1.†

### Spectral properties and discussion of mechanism

**Fig. 1:**
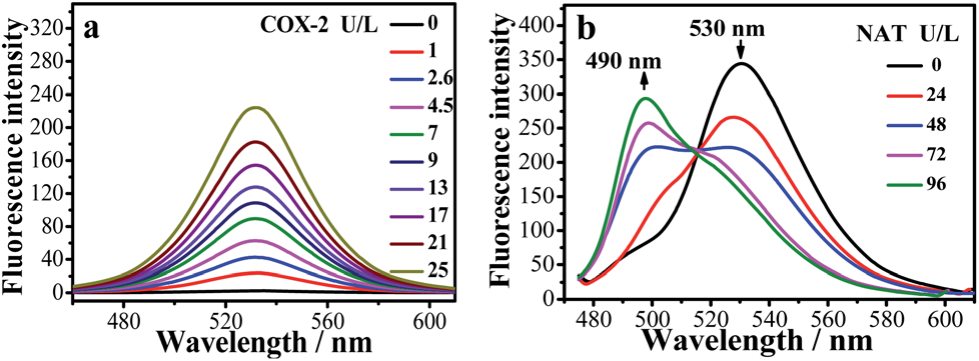
(a) Emission spectra of **3IAN4** (3.0 μM) with COX-2 (0–25 U L^–1^) in buffer at 25 °C. (b) The emission spectra of **3IAN4** (3.0 μM) with NAT (0–96 U L^–1^) in the presence of acetyl-CoA. 37.5 U L^–1^ of COX-2 was first added into solution, which ensured that **3IAN4** fully reacts with NAT.

The fluorescence changes for the **IAN** derivatives (**3IAN2**, **3IAN4**, **3IAN6**, **4IAN2**, **4IAN4** and **4IAN6**) were investigated in PBS buffer solution. In the absence of COX-2 and NAT in the test system, **3IAN2** and **4IAN2** emitted at about 530 nm when excited at 430 nm (Fig. S1a, h, i and Table S1†). However, **3IAN4** (Fig. 1a), **3IAN6** (Table S1†), **4IAN4** (Table S1†) and **4IAN6** (Table S1†) do not produce a distinct emission signal when they were excited at their respective maximum excitation wavelengths (Fig. S1†). Upon adding COX-2, **3IAN2** and **4IAN2** do not change (Fig. S1a†). But, significantly, there was a remarkable “off–on” signal for the emission spectrum of **3IAN4** (Fig. 1a), **3IAN6** (Fig. S1b†), **4IAN4** (Fig. S1c†) and **4IAN6** (Fig. S1d†). For example, **3IAN4** emitted a strong green emission signal at 530 nm about 30 s after encountering COX-2 (Fig. S1g†) when it was excited at 428 nm (Fig. S1i and Table S1†). The intensity of this emission was gradually enhanced with increasing activity of COX-2 from 0 to 21 U L^–1^, resulting in a good linear relationship between the intensity of the emission and the activity of COX-2. The detection limit of **3IAN4** (Fig. S1e†) for COX-2 was 0.83 U L^–1^ (0.10 ng mL^–1^), which is much lower than the previously reported values.^32^,^33^

**Fig. 2:**
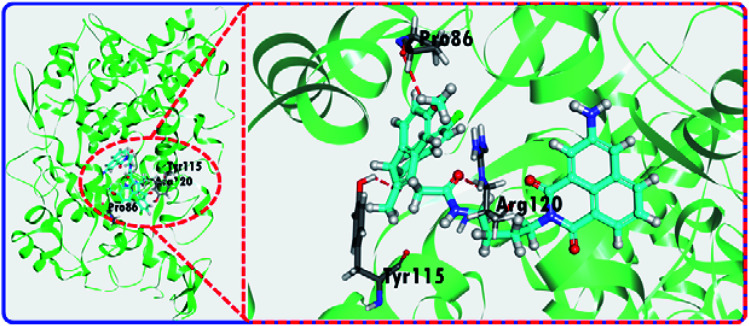
The molecular docking results of **3IAN4** with COX-2 (PDB ID: ; 3NT1).

To illustrate the mechanism of the “off–on” signaling of **3IAN4**, **3IAN6**, **4IAN4** and **4IAN6** for COX-2, **3IAN4** was selected as a typical molecule to optimize the conformation by Gaussian 16. The data from the frontier molecular orbital (FMO) energies of the optimized conformations (Table S2†) indicate that **3IAN4** exists predominately in a stable folded conformation (*i.e.***3IAN4-Folded**) in PBS buffer solution, since the FMO energy of **3IAN4-Folded** was 8.3 kcal mol^–1^ (Table S2†) lower than that of the unfolded conformation (**3IAN4-Unfolded**, see the Fig. 2 and S2a†). Furthermore, the distance of two parallel planes between **IMC** and **ANF-3NH_2_** in the optimized conformation of **3IAN4-Folded** was about 0.4 nm (Fig. S2a†),^18^ which results in a photoinduced electron transfer process (PET) between **IMC** and **ANF-3NH_2_**. More importantly, due to an oscillator strength of 0.001, the electronic transition from the HOMO to LUMO is prohibited (Fig. S2a†).^18^ In other words, the excited state energy could not return from the excited state to the ground state by radiative transition (Fig. S2a†). That is, the emission signal of **3IAN4-Folded** is quenched. Therefore, the molecular docking results (Fig. 2) indicated that **3IAN4** was forced to adopt the unfolded conformation (namely **3IAN4-Unfolded**) within the hydrophobic cavity of COX-2 due to size constraints and multiple hydrogen bonds, the binding affinity of **3IAN4** to COX-2 was found to be 56.33 kcal mol^–1^ (Table S3†),^18^ and the *K*_d_ is 2.461 μM (Fig. S8†). The quantum calculation for **3IAN4-Unfolded** indicates that there was no PET process between **IMC** and **ANF-3NH_2_** (Fig. S2a†). Therefore, its emission signal was restored. Indicating that the remarkable “off–on” signal for **3IAN4** with COX-2 was due to changes in the PET process during conformational changes. The other molecules (**3IAN6**, **4IAN4** and **4IAN6**) all exhibited the same behavior as **3IAN4** due to similar conformational changes. Conversely, according to their FMO energies (Table S2†), unfolded **3IAN2** and **4IAN2** are stable conformations, therefore, **3IAN2** and **4IAN2** do not undergo conformational changes during the recognition process with COX-2.

**Fig. 3:**
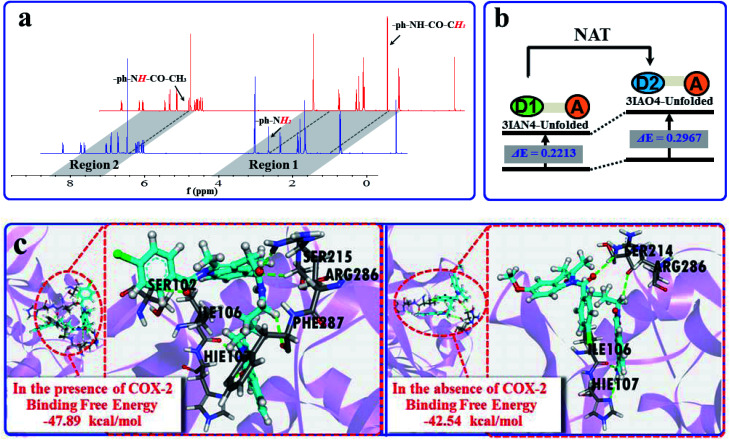
(a) The ^1^H NMR spectra changes of **3IAN4** with NAT under acetyl-CoA. (b) Simple diagram of energy difference (Δ*E*) of **3IAN4-Unfolded** and **3IAO4-Unfolded**. (c) The molecular dynamics calculation results and the binding free energies for the reaction between **3IAN4** and NAT (PDB ID: ; 1BM0) in the presence and absence of COX-2 (PDB ID: ; 3NT1).

Subsequently, the recognition signals of **IAN** derivatives (**3IAN2**, **3IAN4**, **3IAN6**, **4IAN2**, **4IAN4** and **4IAN6**) with NAT were investigated in PBS buffer solution. Fig. 1b and S3† indicate that **3IAN2**, **3IAN4**, **3IAN6**, **4IAN2**, **4IAN4** and **4IAN6** produce similar signal outputs towards NAT. When NAT was available in the test system, the intensity (Fig. 1b) at 530 nm decreased while a new signal at 490 nm gradually increased after about 180 s (Fig. S1g†). With increasing activity of NAT (0–96 U L^–1^, Fig. S1f†), the intensity of the signal at 490 nm gradually increased while the signal at 530 nm decreased. During the addition of NAT, the green signal gradually becomes a blue signal, and *F*_490 nm_/*F*_530 nm_ has a good linear relationship with increasing NAT. The detection limit for **3IAN4** (Fig. S1f†) with NAT was 0.30 U L^–1^ (3.75 ng mL^–1^). Importantly, the time between the two recognition events is significantly shortened to about 150 s compared with previously reported work,^34^ indicating that **3IAN4** is suitable for the imaging of enzymes in living biological systems. ^1^H NMR titration (Fig. 3a) and Gaussian 16 (Fig. S2b† and 3b) were used to illustrate the origin of the ratiometric signals. Fig. 3a indicated that the ph-***NH*_2_** group (region 1 in Fig. 3a) in **3IAN4-Unfolded** was acetylated by NAT to form a –ph-***NH***-CO–CH_3_ group (region 2 in Fig. 3a), a new product **3IAO4-Unfolded** was generated. Furthermore, the energy difference (Δ*E* = *E*_LUMO_ – *E*_HOMO_) of **3IAN4-Unfolded** is lower than that of **3IAO4-Unfolded** (Fig. S2b† and 3b). The binding affinity of **3IAO4** for COX-2 was found to be 53.73 kcal mol^–1^ (Table S3†), which indicates that **3IAO4** can also bind with COX-2.

### The logic input and output signals for COX-2 and NAT in solution

**Fig. 4:**
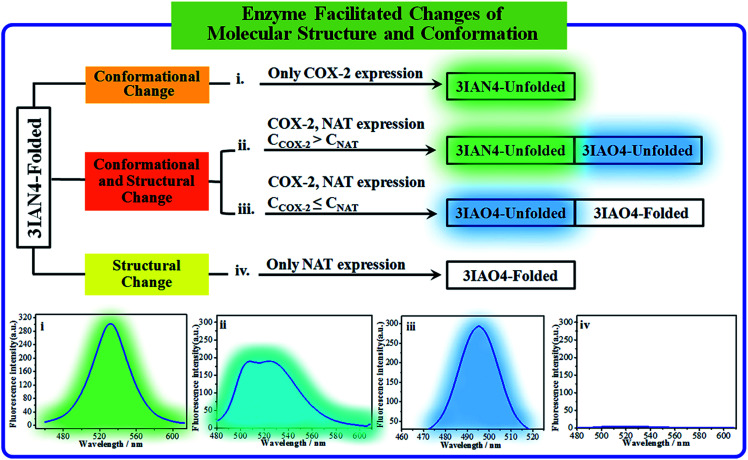
The signal changes for **3IAN4** as a molecular logic gate for COX-2 and NAT in solution.

COX-2 and NAT are two important inflammatory and cancer related enzymes. Therefore, the logical signal changes of the typical molecular example – **3IAN4** towards COX-2 and NAT was investigated in PBS buffer solution. Under the synergistic action of COX-2 and NAT, four logic based signaling phenomena were observed (Fig. 4) which are as follows: (i) when only the activity of COX-2 is present in the system, **3IAN4** produces an emission signal at 530 nm, the green signal (Fig. 4(i)), which is a conformational change. (ii) When COX-2 and NAT co-exist in the system, and the activity of COX-2 is higher than that of NAT, emission signals could be seen at 490 nm and 530 nm, the blue and green signal (Fig. 4(ii)). This is a collaborative process of the conformational and structural change (unfolded and partially reacted). Furthermore, the molecular dynamics results show that the binding free energy of **3IAN4** and NAT in the presence of COX-2 is –47.89 kcal mol^–1^ (Fig. 3c and Table S4†). (iii) As COX-2 and NAT co-exist in the system, and the activity of NAT is higher than or equal to that of COX-2, **3IAN4** produces only one emission signal at 490 nm, a blue signal (Fig. 4(iii)), which is a collaborative process of the conformational and structural change (unfolded and fully reacted). That is, some of **3IAN4-Folded** (no fluorescence) has transformed into **3IAO4-Unfolded** (blue fluorescence) in the presence of NAT and COX-2. And some of **3IAN4-Folded** (no fluorescence) has transformed into **3IAO4-Folded** (no fluorescence) in the presence of NAT (iv) When there is only the activity of NAT in the system, **3IAN4** produces no emission signal (Fig. 4(iv)) under the action of a structural change (fully reacted and folded), which is specific for mild lesions of the liver, that is, mild cirrhosis. In this case, no signal from **3IAN4** for NAT is very helpful to improve the signal contrast during surgery, and to effectively assist the lighting up of the relevant lesion boundaries. The **3IAN4** molecular logic gate was specifically designed to not emit any signal in order to improve surgery (*i.e.* only light up tissue that should be removed). Furthermore, the molecular dynamic results indicate that the binding free energy of **3IAN4** and NAT in the absence of COX-2 is –42.54 kcal mol^–1^ (Fig. 3c and Table S4†) and the *K*_d_ is 2.231 μM (Fig. S8†). These data indicate that the presence of COX-2 does not affect how **3IAN4** reacts with NAT. Thus, **3IAN4** could emit different logic based signals depending on the activity of COX-2 and NAT.

### The logic input and output signals for COX-2 and NAT in living cells

**Fig. 5:**
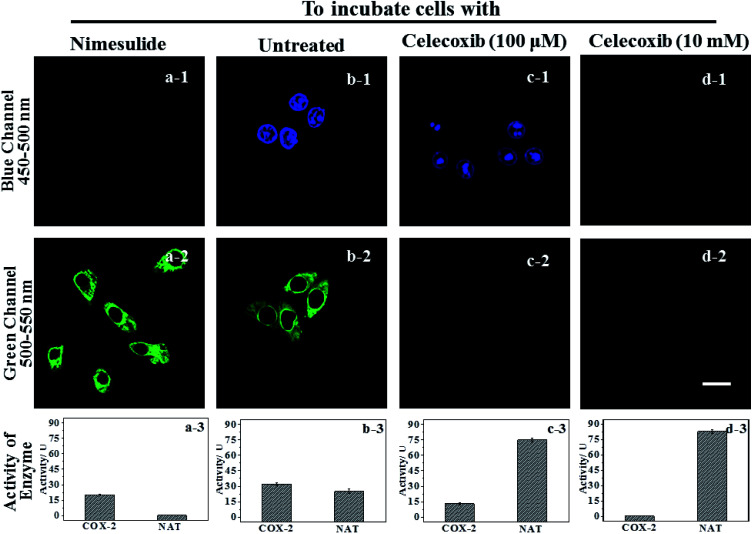
The signal changes of **3IAN4** (3.0 μM) as a molecular logic gate for COX-2 and NAT in HepG2 cells. Blue channel: excitation wavelength = 405 nm, scan range = 450–500 nm. Green channel: excitation wavelength = 488 nm, scan range = 500–550 nm. (a-3), (b-3), (c-3) and (d-3) were the activity of COX-2 and NAT in groups (a–d), which were obtained by ELISA. The cells in (b) group were untreated; the cells in (a) group were treated by nimesulide (10 mM); the cells in (c) group were treated by celecoxib (100 μM). The cells in (d) group were treated by celecoxib (10 mM). The incubation time of celecoxib (10 mM and 100 μM) and nimesulide (10 mM) are 2–6 h, respectively. The appropriate incubation times of celecoxib and nimesulide were ensured by measuring the activities of NAT and COX-2 in every sample as detected by an ELISA assay. Scale: 20 μm.

**IAN** derivatives (Fig. S4–S6†) have low cytotoxicity, excellent photostability and permeability for living cells, so they were incubated with cells and tissues. Treated cells were used for microscopic imaging (Fig. 5), where the content of COX-2 and NAT was regulated by adding inhibitors (celecoxib for COX-2 and nimesulide for NAT).

HepG2 cells were incubated with the inhibitor of NAT, which results in the cells only expressing active COX-2, and no activity for NAT (Fig. 5a-3). Accordingly, there was only a strong green signal at 500–550 nm (Fig. 5a-2). While the blue channel at 450–500 nm was negative (Fig. 5a-1). This process was mainly due to a COX-2 induced conformational change. HepG2 cells that have not been treated express active COX-2 and NAT, and the activity of COX-2 was greater than that of NAT (Fig. 5b-3). These cells emit green and blue signals at 450–500 nm and 500–550 nm after staining with **3IAN4** respectively (Fig. 5b-1 and b-2), which was ascribed to the COX-2 and NAT collaborative process of conformational and structural change (unfolded and partially reacted). Celecoxib was used to inhibit and adjust the activity of COX-2. When the activity of NAT was greater than or equal to that of COX-2 (Fig. 5c-3), only a blue signal was observed (Fig. 5c-2), and the green channel has no signal (Fig. 5c-1), which was due to the COX-2 and NAT conformational and structural change (unfolded and fully reacted). Increasing the amount of celecoxib to completely inhibit the activity of COX-2 in the cells (Fig. 5d-3), resulted in no signal for both channels (blue channel: Fig. 5d-1; green channel Fig. 5d-2). This phenomenon was attributed to a NAT induced structural change and a folded system. Furthermore, all the imaging results in Fig. 5 display a common feature, that is a non-merged signal pattern of green and blue channels. Which can be ascribed to the different cellular locations of COX-2 and NAT, which was verified by the intracellular co-localization imaging (Fig. S11†).

### Using logic to distinguish the boundaries of hepatopathic lesions

**Fig. 6:**
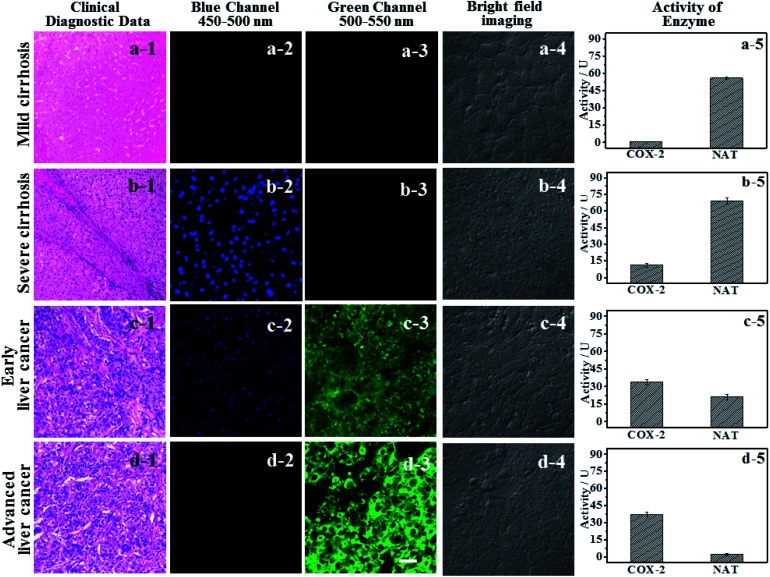
The signal changes of **3IAN4** (10.0 μM) as a molecular-logic gate to differentially monitor cirrhosis and hepatoma. (a) Mild cirrhosis. (b) Severe cirrhosis. (c) Early liver cancer. (d) Advanced liver cancer. (a-1 to d-1) Hematoxylin–eosin staining data. (a-2 to d-2), (a-3 to d-3) and (a-4 to d-4) are microscopic imaging. (a-2 to d-2) Blue channel: excitation wavelength = 405 nm, scan range = 450–500 nm; (a-3 to d-3) green channel: excitation wavelength = 488 nm, scan range = 500–550 nm; (a-4 to d-4) bright field imaging. (a-5 to d-5) were the activity of COX-2 and NAT in groups (a–d), which were obtained by ELISA. Scale: 20 μm.

**Fig. 7:**
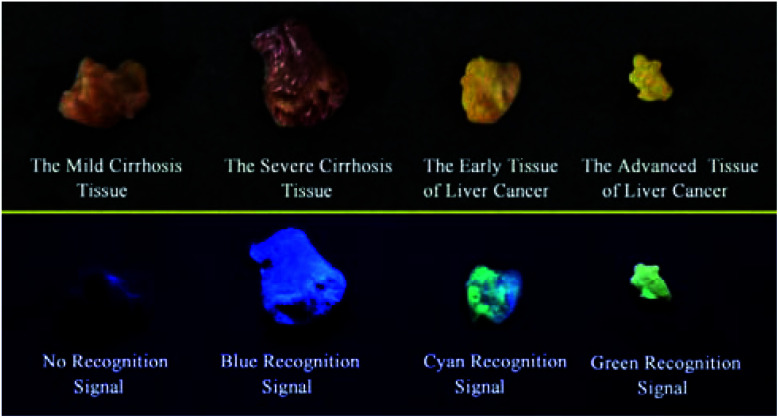
Visualization of tumour resection by the naked eye under ultraviolet illumination.

It is extremely important for successful surgery that the boundaries of cirrhosis and hepatoma are accurately labeled by marking dyes. So, COX-2 and NAT specific-targeting **3IAN4** was used to distinguish the boundaries of the different hepatopathic samples, such as mild cirrhosis, severe cirrhosis, early liver cancer and advanced liver cancer. The activities of COX-2 and NAT in all the samples was detected by ELISA. The stages of all the liver samples were confirmed by the pathological examination after resection (Fig. 6a). The tissue samples were stained with 3.0 μM of **3IAN4** for 15 min at 37 °C, the signals were scanned using two channels (blue channel: 450–500 nm; green channel: 500–550 nm). Fig. 6 shows the signal at various stages during the development of liver cirrhosis and hepatoma tissues at the same incubation time. During the mild cirrhosis (Fig. 6a), no signal for any channel was observed (Fig. 6a-2 to a-4). Since the activity of COX-2 in the sample for this stage are very low and the activity of NAT is high (Fig. 6a-5), which results in only a NAT induced structural change. As the disease progresses (Fig. 6b–d), the activities of COX-2 and NAT in each sample varies. For severe cirrhosis (the activity of COX-2 ≤ the activity of NAT), and results in just a blue signal (Fig. 6b-2), caused by COX-2 and NAT conformational and structural changes. For the early samples of liver cancer (the activity of COX-2 > the activity of NAT), therefore the blue channel (Fig. 6c-2) and green channel (Fig. 6c-3) all emit bright signals, which is also attributed to COX-2 and NAT conformational and structural changes. For the advanced liver cancer tissue (Fig. 6d), the activity of COX-2 is highly expressed and the activity of NAT hardly expressed (Fig. 6d-5). Therefore, just a green signal was observed (Fig. 6d-3), *i.e.* only a molecular conformation change. Therefore, **3IAN4** as molecular-logic gate could output different signals to distinguish the different stages of the hepatopathic samples by microscopic imaging due to the differing activity levels of COX-2 and NAT in the samples. Furthermore, **3IAN4** could distinguish the boundaries of different stages of the hepatopathic samples by naked eye using a hand held lamp (365 nm, Fig. 7 and S10†), validated using ten patients samples. After spraying the sample with **3IAN4** different signals could be seen allowing for differentiation of mild cirrhosis (no signal), severe cirrhosis (blue signal), early liver cancer (cyan signal) and advanced liver cancer (green signal), respectively. Furthermore, the parallel results (Fig. S10†) demonstrate the universality of the signal changes for similar liver samples. Such multiple logic signals produced at various stages of hepatopathy are very important to help distinguish between different lesion boundaries, and are suitable for guiding surgery to effectively remove the lesions.

## Conclusions

In conclusion, based on the clinical requirement to light up the relevant lesion boundaries during operations, we have designed and synthesized a series of novel **IAN** derivatives, which could be used as a visual tool to distinguish between the boundaries of hepatopathic lesions. These molecular logic based signals were produced due to the collaborative conformation and structural change of **IAN** derivatives by hepatopathy-related COX-2 and NAT activity. When only COX-2 exhibits enzymatic activity or the activity of COX-2 exceeds that of NAT in the test system, then the **IAN** derivatives emit a strong green fluorescence signal due to conformational changes of the **IAN** derivatives. When the activity of COX-2 equals or slightly exceeds that of NAT, a green and blue fluorescence signal can be observed in the respective channels because of the collaborative effect of conformation and structural changes of the **IAN** derivatives. With only the activity of NAT expressed in the system, no output signal colour is observed. Therefore, **IAN** derivatives can emit a logical signal correlated with the different liver lesions, that is, different colour output including green, blue and cyan depending on the different activity of COX-2 and NAT enzymes in mild cirrhosis, severe cirrhosis, early liver cancer and advanced liver cancer tissues. Meanwhile, the logic based signals can be explained using Gaussian 16, molecular docking and ^1^H NMR. The logic based signals can be observed during the imaging of cancer cell models with different activities of COX-2 and NAT. In addition, after spraying **IAN** derivatives onto mild cirrhosis, severe cirrhosis, early and late hepatocellular carcinoma tissues, the colour of these diseased tissues changed from colourless, blue, cyan to green as visualised by the unaided eyes under a hand held lamp (365 nm). Consequently, this novel **IAN** derivative could potentially provide a visual tool to help distinguish relevant lesion boundaries during surgery and improve the success rate of hepatopathic resection operations.

## Conflicts of interest

The authors declare no competing financial interests.

## Supplementary Material

Supplementary information
