# Supplementary material for: A molecular-logic gate for COX-2 and NAT based on conformational and structural changes: visualizing the progression of liver disease
Source: Chem Sci. 2020 May 25;11(24):6209–16. doi: 10.1039/d0sc00574f (PMC7480271; doi:10.1039/d0sc00574f)
Supplement: Supplementary file 1 [file SC-011-D0SC00574F-s001.pdf]

Electronic Supplementary Material (ESI) for Chemical Science.
This journal is © The Royal Society of Chemistry 2020

Supporting Information

A molecular-logic gate for COX-2 and NAT based on conformational and structural changes: Visualizing tholore progression of liver disease

Yuehua Chen,[a,†] Yuzhu Wang,[b,†] Yonggang Yang,[a,†] Yuhuan Li,[a] Yafu Wang,[a] Ge Wang,[d] Tony D James,[a,c*] Xiaopeng Xuan,[a] Hua Zhang,[a,*] Yufang Liu[a]

[a] Henan Key Laboratory of Green Chemical Media and Reactions, Ministry of Education; Henan Key Laboratory of Organic Functional Molecules and Drug Innovation; School of Chemistry and Chemical Engineering; School of Physics; Henan Normal University, Xinxiang 453007, P. R. China

[b] Department of Hepatobiliary and pancreatic surgery, Henan Provincial People's Hospital; Zhengzhou University People's Hospital; Henan University People's Hospital, Zhengzhou, Henan, 450003, P. R. China

[c] Department of Chemistry, University of Bath, Bath, BA2 7AY, UK

[d] Xinxiang Medical University, Xinxiang 453000, P. R. China

Corresponding Author's email: t.d.james@bath.ac.uk; zhanghua1106@163.com. † These authors contributed equally.

Contents

1. **Procedures section........S-2**
2. **Synthetic route of IAN derivatives........S-6**
3. **Synthetic procedures for IAN derivatives and intermediates........S-6**
4. **The basic optical data and the response to COX-2........S-12**
5. **The theoretical calculation data S-14**
6. **The response to NAT S-15**
7. **The reproducibility and selectivity S-16**
8. **The photostability, pH-stability and water solubility of 3IAN4 and 3IAO4 ....S-17**
9. **The cytotoxicity of 3IAN4 and 3IAO4 ....S-17**
10. **The solvatochromism of 3IAN4 and 3IAO4 ....S-18**
11. **The binding affinities and binding free energies........S-18**
12. **Imaging and Visualization........S-20**
13. **References........S-22**
14. **Attached spectra........S-23**

1. Procedures section

A.R. grade of solvents and reagents were used in this work. Column chromatographic was used to purify compounds and silica gel (200-300 mesh) was used as fillers. Cyclooxygenase-2 (COX-2, Enzyme Commission Number 3.6.1.3) and N-acetyltransferase (NAT, Enzyme Commission Number 2.3.1.5) were obtained from Sigma Chemical Co. (USA). Doubly purified water was used in all experiments, which was prepared using by a Milli-Q system. IAN derivatives as stock were used in spectrographic determination and cell experiments. HPLC (Agilent 1290 Infinity)-HRMS (Bruker microToF II, Bruker Co., Switzerland) with an auto sampler operated in-line with a quantum triple quadrupole instrument was carried out in mass spectral studies on ESI positive or negative ion mode. NMR spectra were obtained from Avance 400 and 600 MHz spectrometer (Bruker Co., Switzerland).

1.1 Spectrographic determination in vitro.

A Lambd 950 spectrophotometer from PerkinElmer (USA) and a LS-55 spectrophotometer from PerkinElmer (USA) were used to measure absorption spectra and fluorescence spectra, respectively. In all spectral experiments, the final solutions contained < 5 ‰ DMSO. Each experiment was carried out in five replicates (n = 5). The relative fluorescence quantum yields were determined using Rhodamine B (Φ_F = 0.97 in methanol) by the following equation:

$$\Phi_x = \Phi_s(F_x/F_s)(A_s/A_x)(\lambda_{exs}/\lambda_{exx})(n_x/n_s)^2 \quad (1)$$

where Φ represents quantum yield; F is the integrated area under the corrected emission spectrum; A is absorbance at the excitation wavelength; λ_{ex} is the excitation wavelength; n is the refractive index of the solution (because of the low concentrations of the solutions, 10^{-7}-10^{-8} mol/L, the refractive indices of the solutions were replaced with those of the solvents); and the subscripts x and s refer to the unknown and the standard, respectively. The detection limit was calculated by three times the standard deviation divided by the slope of the blank. The data were obtained from replicate experiments (n = 5)

To avoid fluorescence quenching caused by excessive concentration, the concentration (3.0 μM) of probes was used into the all fluorescence spectrum test. To improve the accuracy of the experimental values (absorbance value), that is, the absorbance value is at the range of 0.3-0.7, the concentration (30 μM) of probes was used into the all absorption spectra test.

1.2 Quantum Calculations.

The geometric structure of IAN derivatives was calculated using Gaussian 16 program suite.[S1] The functional ωB97X-D and TZVP basis set was used in the Density functional theoretical (DFT) methods.[S2] The electronic transition energies and corresponding oscillator strengths were calculated by time-dependent density functional theory (TD-DFT) at ωB97X-D/TZVP level. Water is used as solvent in the CPCM calculations to evaluate the solvent effect.[S3]

1.3 Photostability in solution.

IAN derivatives were dissolved in DMSO-water (5:5 v/v) all at concentrations of 3.0 μM. The solutions were irradiated by a 500W iodine-tungsten lamp situated 250 mm away for 7 h. An aqueous solution of sodium nitrite (50 g/L) was placed between the samples and the lamp as a light filter (to cut off the light shorter than 400 nm) and as a heat filter. The photostabilities were expressed in terms of remaining absorption (%) calculated from the changes of absorbance at the absorption maximum before and after irradiation by iodine-tungsten lamp. The absorbance was determined. The data were obtained from replicate experiments (n = 5).

1.4 Photostability in cells.

The fluorescence intensity was determined, after cell staining with 3.0 μM **IAN** derivatives for 30 min at 37 °C under 5% CO_2, by spectral confocal multiphoton microscopy (Olympus, FV1200) with a high-performance mode-locked titanium-sapphire laser source (MaiTai, Spectra-Physics, USA). The change of fluorescence intensity with scan time was determined by spectral confocal multiphoton microscopy (Olympus, FV1200) in the consecutive t-scan mode for 60 min. The intensity of **IAN** derivatives were recorded with the relevant emission. Excitation wavelength: 800 nm with constant intensity. The data were obtained from replicate experiments (n=5).

1.5 Cell and human hepatopathic tissue samples.

HepG 2 cell lines were obtained from the Chinese Academy of Medical Sciences. The human hepatopathic tissue samples from individuals were obtained from Department of Hepatobiliary and pancreatic surgery, Henan Provincial People's Hospital (Zhengzhou, China) and Xinxiang Medical University (Henan, China, ethics statement Reference No. 2015016) was followed for all experiments performed during this study. (1) The first type of experiment: The hepatopathic tissue samples were sliced up to obtain 10 μm deep tissue slices by freezing microtome, and the hepatopathic tissue samples were incubated with 3IAN4 (10 μM of 3IAN4 for 15 min) before imaging. Olympus spectral confocal multiphoton microscope (FV1200) with MaiTai femtosecond laser source (Spectra-Physics) was used for the imaging experiment. (2) The second type of experiment: hematoxylin-eosin staining was used to detect the histopathology of all tissues. (3) The third type of experiment: Tissues were spayed with 3IAN4 (10 μM of 3IAN4 for 15 min). The samples were then imaged under the hand held lamp. All samples were incinerated after the experiment according to the ethics guidelines (Reference No. 2015016).

1.6 Fluorescence Imaging in Cells in hepatopathic tissue samples.

Olympus spectral confocal multiphoton microscope (FV1200) with MaiTai femtosecond laser source (Spectra-Physics) was used in image experiment. Inverted microscope was used in cell and in hepatopathic tissues imaging. The imaging parameters are as follow. Internal PMTs = 16 bit, pixels = 1024 × 1024. Lasers: 405 nm and 488 nm. The scan ranges were ascertained according to fluorescence of dye.

1.7 Cytotoxicity.

HepG 2 cell lines were prepared for cell viability studies in 96-well plates (1×10^5 cells per well that were incubated in 100 μL). The cells were incubated for an additional 24 h with **3IAN4** in different concentrations. Subsequently, 100 μL of 3-(4,5-dimethylthiazol-2-yl)-2,5-diphenyltetrazolium bromide (MTT, Sigma Chemical

Co. U.S.A.) was added into each well, followed by further incubation for 24 h at 37 °C. The DMEM was remove and DMSO (200 μL/well) added to dissolve the reddish-blue crystals. Optical density (OD) was determined by a microplate reader (Spectra Max M5, Molecular Devices) at 570 nm with subtraction of the absorbance of the cell-free blank volume at 630 nm. The results from the six individual experiments were averaged. The relative cell viability (100%) was calculated using the following equation:

$$\text{cell viability}(\%) = (OD_{dye}-OD_{k\text{-}dye})/(OD_{ctrl}-OD_{k\text{-}ctrl}) \times 100 \quad (2)$$

1.8 Molecular docking.

All works of molecular docking were conducted in Yinfo Cloud Platform (http://cloud.yinfotek.com/). The UCSF DOCK 6.9 software was used for the molecular docking. The chemical structure of the small molecule **3IAN4** was drawn by JSME and converted to 3D structure with energy minimization in MMFF94 force field. The crystal structure of COX-2 was taken from the RCSB Protein Data Bank. PDB encoding is 3NT1. The DMS tool was employed to build molecular surface of the receptor using a probe atom with a 1.4 Å radius. The binding pocket was defined by the crystal ligand and spheres were generated filling the site by employing the Sphgen module in UCSF Chimera. A box enclosing the spheres was set with center of (25.455, 20.111, 23.683) and sizes of (-55.760, -62.573, -33.680), within which grids necessary for rapid score evaluation were created by the Grid module. Finally, DOCK 6.9 program was utilized to conduct semi-flexible docking where 10000 different orientations were produced. Clustering analysis were performed (RMSD threshold was set 2.0 Å) for candidate poses and the best scored ones were output.

1.9 Molecular dynamics simulation.

MD was performed using the sander module implemented in the Amber 18 suite, with the ff14SB force field used for the protein system, and the GAFF force field used for the ligand. ANTECHAMBER module was used to calculate ligands 3IAN4 in the presence and absence of COX-2 atom charges. Hydrogen atoms and sodium ions (to neutralize the negative charges) were added to protein with the leap utility. The simulation system was immersed in a truncated octahedral box of TIP3P explicit water, extended 10 Å outside the protein on all sides. To start the MD simulation, initial structure of the complex was relaxed to minimize energy during 10,000 minimization steps (5,000 steepest descent steps, SD, and 5,000 conjugate-gradient steps, CG). After energy minimization, the system was gradually heated in the NVT ensemble from 0 to 300 K over 50 ps using the Berendsen coupling algorithm. This procedure was followed by 50 ps of NPT simulation at 300 K and 1 atm pressure using the Langevin dynamics algorithm. After equilibration, 50ns production MD simulation was performed. In all MD simulations, bonds involving hydrogen atoms were constrained using the SHAKE algorithm. Long range interactions were treated using the particle-mesh Ewald (PME) method and a non-bonded interaction cutoff of 10 Å was used. A time step of 2.0 fs was used and coordinates of the system were saved every 50 ps. Binding free energy calculation of the ligand was performed using the MMGBSA module in amber.

1.10 The incubation time of celecoxib and nimesulide.

In order to build appropriate models for the different activity of NAT and COX-2 in HepG 2 cells, we evaluated the inhibitory effect of celecoxib and nimesulide. Through experimental measurement, the inhibitory concentration of celecoxib to COX-2 (IC50 35 μM) is lower than that of the probe 3IAN4 (IC50 75 μM), indicating that the binding of celecoxib and COX-2 is stronger than that of the probe 3IAN4 and COX-2. The incubation time of celecoxib (10 mM and 100 μM) and nimesulide (10 mM) are 2-6 h. And the activities of NAT and COX-2 in every sample were detected using an ELISA assay to make sure that cell model is the model we want. And then, the appropriate cell models were washed three times by PBS.

1.11 ELISA assay.

(1) The sample preparation: After the normal incubation, the HepG 2 cells were crushed to obtain the supernatant by centrifugation (2000-3000rpm). The supernatant was diluted to a suitable concentration. The enzyme activity was then measured with the ELISA kit of NAT (N-acetyltransferase-like protein ELISA kit, Product code: KS14841, Keshun Biological) and COX-2 (PTGS2/COX-2(Prostaglandin Endoperoxide Synthase 2 ELISA Kit, Product code: E-EL-M0959c, Elabscience®).

(2) Enzyme activity of COX-2: 100 μL of the sample was added to the corresponding plate well and incubated at 37°C for 90 min. Then, the liquid in the plate was discarded, 100 μL of biotinylated antibody working liquid was immediately added and incubated at 37 °C for 60 min. All the liquid was discarded and the sample washed 3 times. Then 100 μL of HRP enzyme binding solution was added to each well, and incubated at 37 °C for 30 min, the liquid was discarded and the sample washed 5 times. Then 90 μL of substrate solution was added to each well and incubated at 37 °C for about 15 min. Finally, 50 μL of termination fluid was added into each well. Then the well was immediately read at 450 nm by a microplate reader and the data processed.

(3) Enzyme activity of NAT: 40 μL of the sample was added to the corresponding plate well and the mixture gently shook. Then, 100 μL of the enzyme-labeled reagent was added to each well, except for blank wells. The plate was sealed by plate film, and incubated at 37 °C for 60 min. The liquid was discarded, ad each well was filled with washing liquid, and left to stand for 30 seconds, before discard the liquid, this process was repeated 5 times. Then 50 μL of the colour reagent A and 50 μL of the colour reagent B was added to each well with gentle mixing, keeping them out of light at 37 °C for 15 min. 50 μL of termination fluid was added into each well. Then the well was immediately read at 450 nm by a microplate reader and the data processed.

2. Synthetic route of IAN derivatives and intermediates

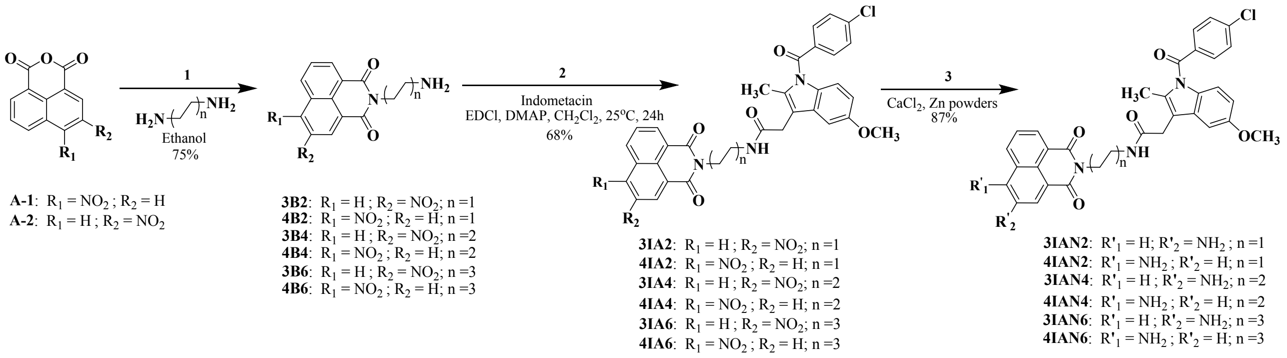

Scheme S1 The synthetic route of **IAN** derivatives.

3. Synthetic procedures for IAN derivatives and intermediates.

The synthesis of A-1

Synthesis of **A-1** was synthesized by the previously reported method.[S4] Yield 91 %. Mp 239 °C. ^{1}H NMR (400 MHz, DMSO-d6), δ: 8.87 (d, J = 8.6 Hz, 1H), 8.41 (d, J = 7.4 Hz, 1H), 8.25 (d, J = 8.5 Hz, 1H), 7.51 (d, J = 8.6 Hz, 1H), 7.05 (d, J = 8.6 Hz, 1H). ^{13}C NMR (100 MHz, DMSO-d6) δ: 149.78, 133.96, 128.67, 127.13, 125.47, 123.98. ESI-MS: m/z calcd for $C_{12}H_5NO_5$: 243.0168, found: 243.0163.

The synthesis of A-2

Synthesis of **A-2** was synthesized by the previously reported method.[S4] Yield 93 %. Mp 247 °C. ^{1}H NMR (400 MHz, DMSO-d6), δ: 8.94 (d, J = 8.6 Hz, 1H), 8.48 (d, J = 7.1 Hz, 1H), 8.32 (d, J = 8.5 Hz, 1H), 7.74 (t, J = 7.9 Hz, 1H), 6.95 (d, J = 8.6 Hz, 1H). ^{13}C NMR (100 MHz, DMSO-d6) δ: 149.54, 133.72, 128.39, 126.90, 125.24, 123.70. ESI-MS: m/z calcd for $C_{12}H_5NO_5$: 243.0168, found: 243.0179.

The synthesis of 3B2

To **A-2** (1.0 mmol) in hot ethanol (20 mL), ethanediamine (1.3 mmol) was added and the mixture was refluxed for 10 h and then allowed to cool. A yellow solid was precipitated and filtered off, and crystallized in DMF/H_2O to yield the desired compound **3B2**. Yield 81%. Mp > 300 °C. ^{1}H NMR (400 MHz, DMSO-d6), δ: 9.09 (d, J = 11.0 Hz, 1H), 8.63 (d, J = 11.0 Hz, 1H), 8.47 (d, J = 11.0 Hz, 1H), 7.89 (d, J = 11.0 Hz, 1H), 7.10 (d, J = 11.0 Hz, 1H), 5.20 (s, 2H), 3.26 (m, 2H), 2.69 (m, 2H). ^{13}C NMR (100 MHz, DMSO-d6) δ: 149.54, 142.65, 134.24, 134.20, 133.68, 128.10, 126.56, 124.90, 123.45, 45.32, 43.14. ESI-MS: m/z calcd for $C_{14}H_{11}N_3O_4$: 285.0750, found: 285.0756.

The synthesis of 4B2

To **A-1** (1.0 mmol) in hot ethanol (20 mL), ethanediamine (1.3 mmol) was added and the mixture was refluxed for 10 h and then allowed to cool. A yellow solid was precipitated and filtered off, and crystallized in DMF/H_2O to yield the desired compound **4B2**. Yield 81%. Mp > 300 ^oC. ^1H NMR (400 MHz, DMSO-d6), δ: 9.08 (d, J = 11.0 Hz, 1H), 8.47 (d, J = 11.0 Hz, 1H), 8.24 (d, J = 11.0 Hz, 1H), 7.88 (d, J = 11.0 Hz, 1H), 7.10 (d, J = 11.0 Hz, 1H), 5.20 (s, 2H), 3.26 (m, 2H), 2.69 (m, 2H). ^{13}C NMR (100 MHz, DMSO-d6) δ: 149.68, 142.74, 134.37, 133.82, 128.29, 126.70, 125.04, 123.55, 45.41, 43.28. ESI-MS: m/z calcd for $C_{14}H_{11}N_3O_4$: 285.0750, found: 285.0754.

The synthesis of 3B4

To **A-2** (1.0 mmol) in hot ethanol (20 mL), butanediamine (1.3 mmol) was added and the mixture was refluxed for 11 h and then allowed to cool. A yellow solid was precipitated and filtered off, and crystallized in DMF/H_2O to yield the desired compound **3B4**. Yield 75%. Mp > 300 ^oC. ^1H NMR (400 MHz, DMSO-d6), δ: 9.00 (d, J = 8.6 Hz, 1H), 8.55 (d, J = 7.1 Hz, 1H), 8.39 (d, J = 8.5 Hz, 1H), 7.81 (t, J = 7.9 Hz, 1H), 7.02 (d, J = 8.6 Hz, 1H), 5.20 (s, 2H), 3.15 (m, 2H), 2.47 (m, 2H), 1.59-1.55 (m, 4H). ^{13}C NMR (100 MHz, DMSO-d6) δ: 149.54, 142.65, 134.22, 133.72, 128.44, 126.90, 125.24, 123.70, 45.44, 43.30, 25.36, 24.93. ESI-MS: m/z calcd for $C_{16}H_{15}N_3O_4$: 313.1063, found: 313.1069.

The synthesis of 4B4

To **A-1** (1.0 mmol) in hot ethanol (20 mL), butanediamine (1.3 mmol) was added and the mixture was refluxed for 11 h and then allowed to cool. A yellow solid was precipitated and filtered off, and crystallized in DMF/H_2O to yield the desired compound **4B4**. Yield 77%. Mp > 300 ^oC. ^1H NMR (400 MHz, DMSO-d6), δ: 8.99 (d, J = 11.0 Hz, 1H), 8.54 (d, J = 11.0 Hz, 1H), 8.38 (d, J = 11.0 Hz, 1H), 7.80 (d, J = 11.0 Hz, 1H), 6.99 (d, J = 11.0 Hz, 1H), 5.24 (s, 2H), 3.15 (m, 2H), 2.47 (m, 2H), 1.57 (m, 4H). ^{13}C NMR (100 MHz, DMSO-d6) δ: 149.67, 142.75, 134.39, 133.82, 128.61, 127.05, 125.34, 123.84, 43.45. ESI-MS: m/z calcd for $C_{16}H_{15}N_3O_4$: 313.1063, found: 313.1057.

The synthesis of 3B6

To **A-2** (1.0 mmol) in hot ethanol (20 mL), hexamethylenediamine (1.3 mmol) was added and the mixture was refluxed for 14 h and then allowed to cool. A yellow solid was precipitated and filtered off, and crystallized in DMF/H_2O to yield the desired compound **3B6**. Yield 69%. Mp > 300 ^oC. ^1H NMR (400 MHz, DMSO-d6), δ: 9.00 (d, J = 11.0 Hz, 1H), 8.55 (d, J = 11.0 Hz, 1H), 8.39 (d, J = 11.0 Hz, 1H), 7.81 (d, J = 11.0 Hz, 1H), 7.02 (d, J = 11.0 Hz, 1H), 5.22 (s, 2H), 3.15 (m, 2H), 2.47 (m, 2H), 1.57 (m, 4H), 1.31 (m, 4H). ^{13}C NMR (100 MHz, DMSO-d6) δ: 149.54, 142.60, 134.22, 134.20, 133.68, 128.44, 126.85, 125.24, 123.70, 45.44, 43.26, 32.14, 30.01, 25.32, 24.88. ESI-MS: m/z calcd for $C_{18}H_{19}N_3O_4$: 341.1376, found: 341.1377.

The synthesis of 4B6

To **A-1** (1.0 mmol) in hot ethanol (20 mL), hexamethylenediamine (1.3 mmol) was added and the mixture was refluxed for 14 h and then allowed to cool. A yellow solid was precipitated and filtered off, and crystallized in DMF/H_2O to yield the desired compound **4B6**. Yield 62%. Mp > 300 ^oC. ^1H NMR (400 MHz, DMSO-d6), δ: 9.00 (d, J = 11.0 Hz, 1H), 8.55 (d, J = 11.0 Hz, 1H), 8.39 (d, J = 11.0 Hz, 1H), 7.81 (d, J = 11.0 Hz, 1H), 6.98 (d, J = 11.0 Hz, 1H), 5.30 (s, 2H), 3.10 (m, 2H), 2.42 (m, 2H), 1.57 (m, 4H), 1.31 (m, 4H). ^{13}C NMR (100 MHz, DMSO-d6) δ: 149.89, 142.94,

134.53, 134.07, 128.74, 127.20, 125.58, 124.04, 45.78, 30.36, 25.66, 25.23. ESI-MS: m/z calcd for $C_{18}H_{19}N_3O_4$: 341.1376, found: 341.1379.

The synthesis of 3IA2

To a solution of **3B2** (0.36 mmol) in 20 mL CH_2Cl_2, indomethacin (0.42 mmol), 1-(3-dimethylaminopropyl)-3-ethylcarbodiimide hydrochloride (EDCl) (0.70 mmol), and 4-dimethylaminopyridine (DMAP) (0.70 mmol) were added. The solution was stirred at room temperature under nitrogen for 24 h and then concentrated. The residue was purified by silica gel column chromatography by using CH_2Cl_2/CH_3OH 60:1 (v/v) as eluent, affording **3IA2** as yellow solid. Yield 69 %. Mp > 300 °C. ^1H NMR (400 MHz, $CDCl_3$), δ 9.00 (d, J = 8.5 Hz, 1H), 8.52 (d, J = 8.5 Hz, 1H), 8.42 (d, J = 8.5 Hz, 1H), 7.82 (d, J = 8.5 Hz, 1H), 7.70 (d, J = 8.5 Hz, 2H), 7.51 (d, J = 8.5 Hz, 2H), 7.02 (d, J = 8.5 Hz, 1H), 6.95 (dd, J = 13.3, 2.4 Hz, 2H), 6.87 (d, J = 9.0 Hz, 1H), 6.82 (s, 1H), 3.83 (s, 2H), 3.81 (s, 3H), 3.13 (d, J = 8.5 Hz, 2H), 2.66 (d, J = 8.5 Hz, 2H), 2.47 (s, 3H). ^{13}C NMR (100 MHz, $CDCl_3$) δ: Unknown NMR (400 MHz,) δ 171.61, 167.70, 154.10, 149.49, 142.65, 140.16, 137.62, 135.10, 134.22, 133.72, 131.31, 129.35, 128.75, 126.85, 125.24, 123.70, 113.45, 112.19, 106.41, 100.63, 55.86, 45.42, 43.28, 31.69. ESI-MS: m/z calcd for $C_{33}H_{25}ClN_4O_7$: 624.1412, found: 624.1417.

The synthesis of 4IA2

To a solution of **4B2** (0.36 mmol) in 20 mL CH_2Cl_2, indomethacin (0.42 mmol), 1-(3-dimethylaminopropyl)-3-ethylcarbodiimide hydrochloride (EDCl) (0.70 mmol), and 4-dimethylaminopyridine (DMAP) (0.70 mmol) were added. The solution was stirred at room temperature under nitrogen for 24 h and then concentrated. The residue was purified by silica gel column chromatography by using CH_2Cl_2/CH_3OH 60:1 (v/v) as eluent, affording **4IA2** as yellow solid. Yield 73 %. Mp > 300 °C. ^1H NMR (400 MHz, $CDCl_3$), δ: 9.06 (d, J = 8.5 Hz, 1H), 8.52 (d, J = 8.5 Hz, 1H), 8.42 (d, J = 8.5 Hz, 1H), 7.85 (d, J = 8.5 Hz, 1H), 7.73 (d, J = 8.5 Hz, 2H), 7.54 (d, J = 8.5 Hz, 2H), 7.02 (d, J = 8.5 Hz, 1H), 6.95 (dd, J = 13.3, 2.4 Hz, 2H), 6.87 (d, J = 9.0 Hz, 1H), 6.82 (s, 1H), 3.83 (s, 2H), 3.81 (s, 3H), 3.13 (d, J = 8.5 Hz, 2H), 2.66 (d, J = 8.5 Hz, 2H), 2.47 (s, 3H). ^{13}C NMR (100 MHz, $CDCl_3$) δ: 171.61, 167.70, 154.10, 142.91, 140.50, 137.88, 135.45, 135.41, 134.53, 133.99, 131.67, 129.61, 129.11, 128.70, 127.24, 125.50, 124.05, 113.71, 112.53, 106.68, 100.99, 55.78, 45.42, 43.26, 31.69. ESI-MS: m/z calcd for $C_{33}H_{25}ClN_4O_7$: 624.1412, found: 624.1419.

The synthesis of 3IA4

To a solution of **3B4** (0.36 mmol) in 20 mL CH_2Cl_2, indomethacin (0.42 mmol), 1-(3-dimethylaminopropyl)-3-ethylcarbodiimide hydrochloride (EDCl) (0.70 mmol), and 4-dimethylaminopyridine (DMAP) (0.70 mmol) were added. The solution was stirred at room temperature under nitrogen for 24 h and then concentrated. The residue was purified by silica gel column chromatography by using CH_2Cl_2/CH_3OH 60:1 (v/v) as eluent, affording **3IA4** as yellow solid. Yield 68 %. Mp > 300 °C. ^1H NMR (400 MHz, $CDCl_3$), δ: 9.00 (d, *J* = 8.5 Hz, 1H), 8.52 (d, *J* = 8.5 Hz, 1H), 8.42 (d, *J* = 8.5 Hz, 1H), 7.82 (d, *J* = 8.5 Hz, 1H), 7.70 (d, *J* = 8.5 Hz, 2H), 7.51 (d, *J* = 8.5 Hz, 2H), 7.02 (d, *J* = 8.5 Hz, 1H), 6.95 (dd, *J* = 13.3, 2.4 Hz, 2H), 6.87 (d, *J* = 9.0 Hz, 1H), 6.82 (s, 1H), 3.83 (s, 2H), 3.81 (s, 3H), 3.13 (d, *J* = 8.5 Hz, 2H), 2.66 (d, *J* = 8.5 Hz, 2H), 2.47 (s, 3H), 1.51 (m, 4H). ^{13}C NMR (100 MHz, $CDCl_3$) δ: 171.57, 167.74, 154.08, 149.54, 142.65, 140.21, 137.65, 135.10, 134.22, 133.72, 131.32, 131.11, 129.34, 128.80, 128.44, 126.90, 125.24, 123.70,

113.45, 112.24, 106.39, 100.68, 55.83, 45.44, 43.30, 31.74, 25.36, 24.93, 12.79. ESI-MS: m/z calcd for $C_{35}H_{29}ClN_4O_7$: 652.1725, found: 652.1729.

The synthesis of 4IA4

To a solution of **4B4** (0.36 mmol) in 20 mL CH_2Cl_2, indomethacin (0.42 mmol), 1-(3-dimethylaminopropyl)-3-ethylcarbodiimide hydrochloride (EDCl) (0.70 mmol), and 4-dimethylaminopyridine (DMAP) (0.70 mmol) were added. The solution was stirred at room temperature under nitrogen for 24 h and then concentrated. The residue was purified by silica gel column chromatography by using CH_2Cl_2/CH_3OH 60:1 (v/v) as eluent, affording **4IA4** as yellow solid. Yield 78 %. Mp > 300 °C. ^1H NMR (400 MHz, $CDCl_3$), δ: 9.04 (d, J = 8.5 Hz, 1H), 8.52 (d, J = 8.5 Hz, 1H), 8.42 (d, J = 8.5 Hz, 1H), 7.82 (d, J = 8.5 Hz, 1H), 7.70 (d, J = 8.5 Hz, 2H), 7.51 (d, J = 8.5 Hz, 2H), 7.02 (d, J = 8.5 Hz, 1H), 6.95 (dd, J = 13.3, 2.4 Hz, 2H), 6.87 (d, J = 9.0 Hz, 1H), 6.82 (s, 1H), 3.83 (s, 2H), 3.81 (s, 3H), 3.13 (d, J = 8.5 Hz, 2H), 2.66 (d, J = 8.5 Hz, 2H), 2.47 (s, 3H), 1.51 (m, 4H). ^{13}C NMR (100 MHz, $CDCl_3$) δ: 171.30, 167.87, 154.53, 150.06, 142.76, 140.40, 137.53, 134.93, 134.65, 133.84, 131.25, 129.67, 128.66, 128.37, 126.56, 125.26, 123.91, 113.74, 112.16, 106.69, 100.63, 55.67, 45.22, 42.85, 31.83, 25.32, 25.05, 13.01. ESI-MS: m/z calcd for $C_{35}H_{29}ClN_4O_7$: 652.1725, found: 652.1718.

The synthesis of 3IA6

To a solution of **3B6** (0.36 mmol) in 20 mL CH_2Cl_2, indomethacin (0.42 mmol), 1-(3-dimethylaminopropyl)-3-ethylcarbodiimide hydrochloride (EDCl) (0.70 mmol), and 4-dimethylaminopyridine (DMAP) (0.70 mmol) were added. The solution was stirred at room temperature under nitrogen for 24 h and then concentrated. The residue was purified by silica gel column chromatography by using CH_2Cl_2/CH_3OH 60:1 (v/v) as eluent, affording **3IA6** as yellow solid. Yield 62 %. Mp > 300 °C. ^1H NMR (400 MHz, $CDCl_3$), δ: 9.00 (d, J = 8.5 Hz, 1H), 8.52 (d, J = 8.5 Hz, 1H), 8.42 (d, J = 8.5 Hz, 1H), 7.82 (d, J = 8.5 Hz, 1H), 7.70 (d, J = 8.5 Hz, 2H), 7.51 (d, J = 8.5 Hz, 2H), 7.02 (d, J = 8.5 Hz, 1H), 6.95 (dd, J = 13.3, 2.4 Hz, 2H), 6.87 (d, J = 9.0 Hz, 1H), 6.82 (s, 1H), 3.83 (s, 2H), 3.81 (s, 3H), 3.13 (d, J = 8.5 Hz, 2H), 2.66 (d, J = 8.5 Hz, 2H), 2.47 (s, 3H), 1.51 (m, 4H), 1.41 (m, 4H). ^{13}C NMR (100 MHz, $CDCl_3$) δ: 171.57, 167.70, 154.15, 149.44, 142.63, 140.21, 137.70, 135.07, 134.22, 133.82, 131.30, 131.06, 129.20, 128.75, 128.25, 126.77, 125.32, 123.72, 113.45, 112.19, 106.41, 100.76, 55.77, 45.44, 43.24, 31.77, 25.30, 24.96, 20.88, 20.50, 12.79. ESI-MS: m/z calcd for $C_{37}H_{33}ClN_4O_7$: 680.2038, found: 680.2030.

The synthesis of 4IA6

To a solution of **4B4** (0.36 mmol) in 20 mL CH_2Cl_2, indomethacin (0.42 mmol), 1-(3-dimethylaminopropyl)-3-ethylcarbodiimide hydrochloride (EDCl) (0.70 mmol), and 4-dimethylaminopyridine (DMAP) (0.70 mmol) were added. The solution was stirred at room temperature under nitrogen for 24 h and then concentrated. The residue was purified by silica gel column chromatography by using CH_2Cl_2/CH_3OH 60:1 (v/v) as eluent, affording **4IA6** as yellow solid. Yield 74 %. Mp > 300 °C. ^1H NMR (400 MHz, $CDCl_3$), δ: 9.04 (d, J = 8.5 Hz, 1H), 8.56 (d, J = 8.5 Hz, 1H), 8.46 (d, J = 8.5 Hz, 1H), 7.82 (d, J = 8.5 Hz, 1H), 7.70 (d, J = 8.5 Hz, 2H), 7.51 (d, J = 8.5 Hz, 2H), 7.02 (d, J = 8.5 Hz, 1H), 6.95

(dd, J = 13.3, 2.4 Hz, 2H), 6.87 (d, J = 9.0 Hz, 1H), 6.82 (s, 1H), 3.83 (s, 2H), 3.81 (s, 3H), 3.13 (d, J = 8.5 Hz, 2H), 2.66 (d, J = 8.5 Hz, 2H), 2.47 (s, 3H), 1.51 (m, 4H), 1.40 (m, 4H). ^{13}C NMR (100 MHz, $CDCl_3$) δ: Unknown NMR (400 MHz,) δ 171.59, 167.82, 154.18, 149.49, 142.65, 140.15, 137.57, 135.12, 134.24, 133.68, 131.31, 130.98, 129.57, 128.95, 128.27, 126.73, 125.24, 123.61, 113.43, 112.24, 106.39, 100.71, 55.91, 45.29, 43.12, 31.72, 25.36, 24.96, 20.91, 20.50, 12.74. ESI-MS: m/z calcd for $C_{37}H_{33}ClN_4O_7$: 680.2038, found: 680.2031.

The synthesis of 3IAN2

To a solution of **3IA2** (1.0 mmol) in ethanol (20 mL), excessive Zn powders and crystallization of calcium chloride were added and the mixture was refluxed for 3.0 h. After then, the solvent was removed. The solid was purified by silica gel column chromatography by using CH_3OH/CH_2Cl_2=1:10 as eluent, affording **3IAN2** as yellow solid. Yield 85 %. Mp > 300°C. ^{1}H NMR (400 MHz, $CDCl_3$), δ: 9.00 (d, J = 8.5 Hz, 1H), 8.52 (d, J = 8.5 Hz, 1H), 8.42 (d, J = 8.5 Hz, 1H), 7.82 (d, J = 8.5 Hz, 1H), 7.70 (d, J = 8.5 Hz, 2H), 7.51 (d, J = 8.5 Hz, 2H), 7.02 (d, J = 8.5 Hz, 1H), 6.97 (d, J = 2.4 Hz, 2H), 6.87 (d, J = 9.0 Hz, 1H), 6.82 (s, 1H), 3.83 (s, 2H), 3.81 (s, 3H), 3.13 (d, J = 8.5 Hz, 2H), 2.66 (d, J = 8.5 Hz, 2H), 2.57 (s, 2H), 2.47 (s, 3H). ^{13}C NMR (100 MHz, $CDCl_3$) δ: 171.61, 167.74, 156.03, 151.36, 144.46, 142.06, 139.50, 137.07, 136.06, 135.68, 133.19, 132.91, 131.20, 130.70, 130.34, 128.67, 126.98, 125.50, 115.32, 114.09, 108.24, 102.53, 55.81, 45.49, 43.33, 31.66. ESI-MS: m/z calcd for $C_{33}H_{27}ClN_4O_5$: 594.1670, found: 594.1675.

The synthesis of 4IAN2

To a solution of **4IA2** (1.0 mmol) in ethanol (20 mL), excessive Zn powders and crystallization of calcium chloride were added and the mixture was refluxed for 3.0 h. After then, the solvent was removed. The solid was purified by silica gel column chromatography by using CH_3OH/CH_2Cl_2=1:10 as eluent, affording **4IAN2** as yellow solid. Yield 72 %. Mp > 300 °C. ^{1}H NMR (400 MHz, $CDCl_3$), δ: 9.00 (d, J = 8.5 Hz, 1H), 8.52 (d, J = 8.5 Hz, 1H), 8.42 (d, J = 8.5 Hz, 1H), 7.82 (d, J = 8.5 Hz, 1H), 7.70 (d, J = 8.5 Hz, 2H), 7.51 (d, J = 8.5 Hz, 2H), 7.02 (d, J = 8.5 Hz, 1H), 6.97 (d, J = 2.4 Hz, 2H), 6.87 (d, J = 9.0 Hz, 1H), 6.82 (s, 1H), 3.83 (s, 2H), 3.81 (s, 3H), 3.13 (d, J = 8.5 Hz, 2H), 2.66 (d, J = 8.5 Hz, 2H), 2.57 (s, 2H), 2.47 (s, 3H). ^{13}C NMR (100 MHz, $CDCl_3$) δ: 171.53, 167.85, 155.90, 151.38, 144.57, 141.88, 139.50, 136.94, 136.18, 135.53, 133.25, 132.84, 131.31, 130.68, 130.25, 128.75, 127.13, 125.50, 115.27, 114.08, 108.17, 102.67, 100.50, 55.86, 45.44, 43.33, 31.77. ESI-MS: m/z calcd for $C_{33}H_{27}ClN_4O_5$: 594.1670, found: 594.1679.

The synthesis of 3IAN4

To a solution of **3IA4** (1.0 mmol) in ethanol (20 mL), excessive Zn powders and crystallization of calcium chloride were added and the mixture was refluxed for 3.0 h. After then, the solvent was removed. The solid was purified by silica gel column chromatography by using CH_3OH/CH_2Cl_2=1:10 as eluent, affording **3IAN4** as yellow solid. Yield 75 %. Mp > 300 °C. ^{1}H NMR (400 MHz, $CDCl_3$), δ: 9.00 (d, J = 8.5 Hz, 1H), 8.52 (d, J = 8.5 Hz, 1H), 8.42 (d, J = 8.5 Hz, 1H), 7.82 (d, J = 8.5 Hz, 1H), 7.70 (d, J = 8.5 Hz, 2H), 7.51 (d, J = 8.5 Hz, 2H), 7.02 (d, J = 8.5 Hz, 1H), 6.95 (dd, J = 13.3, 2.4 Hz, 2H), 6.87 (d, J = 9.0 Hz, 1H), 6.82 (s, 1H), 3.83 (s, 2H), 3.81 (s, 3H), 3.13 (d, J = 8.5 Hz, 2H), 2.66 (d, J = 8.5 Hz, 2H), 2.57 (s, 2H), 2.47 (s, 3H), 1.51 (m, 4H). ^{13}C NMR (100 MHz, $CDCl_3$) δ: Unknown NMR (400 MHz,) δ 171.69, 167.74, 154.00, 149.44, 142.54, 140.11, 137.62, 134.97, 134.35, 133.70, 131.47, 131.23, 131.04, 129.22,

128.80, 128.29, 126.79, 125.30, 123.70, 113.27, 112.32, 106.37, 100.71, 55.38, 45.68, 43.09, 31.62, 25.72, 25.00, 12.99. ESI-MS: m/z calcd for $C_{35}H_{31}ClN_4O_5$: 622.1983, found: 622.1989.

The synthesis of 4IAN4

To a solution of **4IA4** (1.0 mmol) in ethanol (20 mL), excessive Zn powders and crystallization of calcium chloride were added and the mixture was refluxed for 3.0 h. After then, the solvent was removed. The solid was purified by silica gel column chromatography by using CH_3OH/CH_2Cl_2=1:10 as eluent, affording **4IAN4** as yellow solid. Yield 75 %. Mp > 300 ^{o}C. ^{1}H NMR (400 MHz, $CDCl_3$), δ: 9.04 (d, J = 8.5 Hz, 1H), 8.52 (d, J = 8.5 Hz, 1H), 8.42 (d, J = 8.5 Hz, 1H), 7.82 (d, J = 8.5 Hz, 1H), 7.70 (d, J = 8.5 Hz, 2H), 7.51 (d, J = 8.5 Hz, 2H), 7.02 (d, J = 8.5 Hz, 1H), 6.95 (dd, J = 13.3, 2.4 Hz, 2H), 6.87 (d, J = 9.0 Hz, 1H), 6.82 (s, 1H), 3.83 (s, 2H), 3.81 (s, 3H), 3.13 (d, J = 8.5 Hz, 2H), 2.66 (d, J = 8.5 Hz, 2H), 2.56 (s, 2H), 2.47 (s, 3H), 1.51 (m, 4H). ^{13}C NMR (100 MHz, $CDCl_3$) δ: Unknown NMR (400 MHz,) δ 171.59, 167.80, 154.55, 149.93, 142.55, 140.24, 137.71, 134.91, 134.14, 133.64, 131.12, 129.30, 128.80, 128.31, 127.04, 125.22, 123.96, 113.80, 111.98, 106.42, 100.79, 55.72, 45.27, 43.24, 31.52, 25.41, 24.65, 12.93. ESI-MS: m/z calcd for $C_{35}H_{31}ClN_4O_5$: 622.1983, found: 622.1976.

The synthesis of 3IAN6

To a solution of **3IA6** (1.0 mmol) in ethanol (20 mL), excessive Zn powders and crystallization of calcium chloride were added and the mixture was refluxed for 3.0 h. After then, the solvent was removed. The solid was purified by silica gel column chromatography by using CH_3OH/CH_2Cl_2=1:10 as eluent, affording **3IAN6** as yellow solid. Yield 77 %. Mp > 300 ^{o}C. ^{1}H NMR (400 MHz, $CDCl_3$), δ: 9.00 (d, *J* = 8.5 Hz, 1H), 8.52 (d, *J* = 8.5 Hz, 1H), 8.42 (d, *J* = 8.5 Hz, 1H), 7.82 (d, *J* = 8.5 Hz, 1H), 7.70 (d, *J* = 8.5 Hz, 2H), 7.51 (d, *J* = 8.5 Hz, 2H), 7.02 (d, *J* = 8.5 Hz, 1H), 6.95 (dd, *J* = 13.3, 2.4 Hz, 2H), 6.91 (d, J = 9.0 Hz, 1H), 6.88 (s, 1H), 3.83 (s, 2H), 3.81 (s, 3H), 3.13 (d, *J* = 8.5 Hz, 2H), 2.66 (d, *J* = 8.5 Hz, 2H), 2.57 (s, 2H), 2.47 (s, 3H), 1.51 (m, 4H), 1.41 (m, 4H). ^{13}C NMR (100 MHz, $CDCl_3$) δ: 171.69, 167.91, 153.45, 148.70, 142.43, 139.95, 137.21, 135.20, 134.18, 133.41, 131.42, 129.42, 128.67, 127.91, 126.93, 125.43, 123.70, 113.45, 112.69, 106.71, 100.44, 55.70, 45.22, 43.45, 31.72, 25.46, 24.45, 20.93, 20.45, 12.95. ESI-MS: m/z calcd for $C_{37}H_{35}ClN_4O_5$: 650.2296, found: 650.2299.

The synthesis of 4IAN6

To a solution of **4IA4** (1.0 mmol) in ethanol (20 mL), excessive Zn powders and crystallization of calcium chloride were added and the mixture was refluxed for 3.0 h. After then, the solvent was removed. The solid was purified by silica gel column chromatography by using CH_3OH/CH_2Cl_2=1:10 as eluent, affording **4IAN6** as yellow solid. Yield 71%. Mp > 300 ^{o}C.

^{1}H NMR (400 MHz, $CDCl_3$), δ: 9.04 (d, J = 8.5 Hz, 1H), 8.56 (d, J = 8.5 Hz, 1H), 8.46 (d, J = 8.5 Hz, 1H), 7.82 (d, J = 8.5 Hz, 1H), 7.70 (d, J = 10.6 Hz, 2H), 7.51 (d, J = 8.5 Hz, 2H), 7.02 (d, J = 8.5 Hz, 1H), 6.95 (dd, J = 13.3, 2.4 Hz, 2H), 6.87 (d, J = 9.0 Hz, 1H), 6.82 (s, 1H), 3.83 (s, 2H), 3.81 (s, 3H), 3.13 (d, J = 8.5 Hz, 2H), 2.66 (d, J = 8.5 Hz, 2H), 2.57 (s, 2H), 2.47 (s, 3H), 1.51 (m, 4H), 1.41 (m, 4H). ^{13}C NMR (100 MHz, $CDCl_3$) δ: 171.41, 167.47, 154.18, 149.49, 141.15, 138.92, 136.21, 133.76, 133.02, 132.28, 130.03, 128.07, 127.57, 126.86, 125.37, 124.41, 122.43, 113.55,

112.33, 106.17, 100.71, 55.91, 45.33, 43.16, 31.56, 25.66, 24.44, 21.18, 20.24, 12.57. ESI-MS: m/z calcd for $C_{37}H_{35}ClN_4O_5$: 650.2296, found: 650.2287.

4. The basic optical data and the response to COX-2

Table S1. The basic optical data

	Fomula	λ_{ex}/nm	ε / M^{-1} cm^{-1}	λ_{em}/nm	Φ
**3IAN2**	$C_{33}H_{27}ClN_4O_5$	430	2.21×10^4	530	0.61
**4IAN2**	$C_{33}H_{27}ClN_4O_5$	431	1.81×10^4	527	0.59
**3IAN4**	$C_{35}H_{31}ClN_4O_5$	428	2.25×10^4	-	-
**4IAN4**	$C_{35}H_{31}ClN_4O_5$	430	2.17×10^4	-	-
**3IAN6**	$C_{37}H_{35}ClN_4O_5$	428	1.98×10^4	-	-
**4IAN6**	$C_{37}H_{35}ClN_4O_5$	431	1.97×10^4	-	-

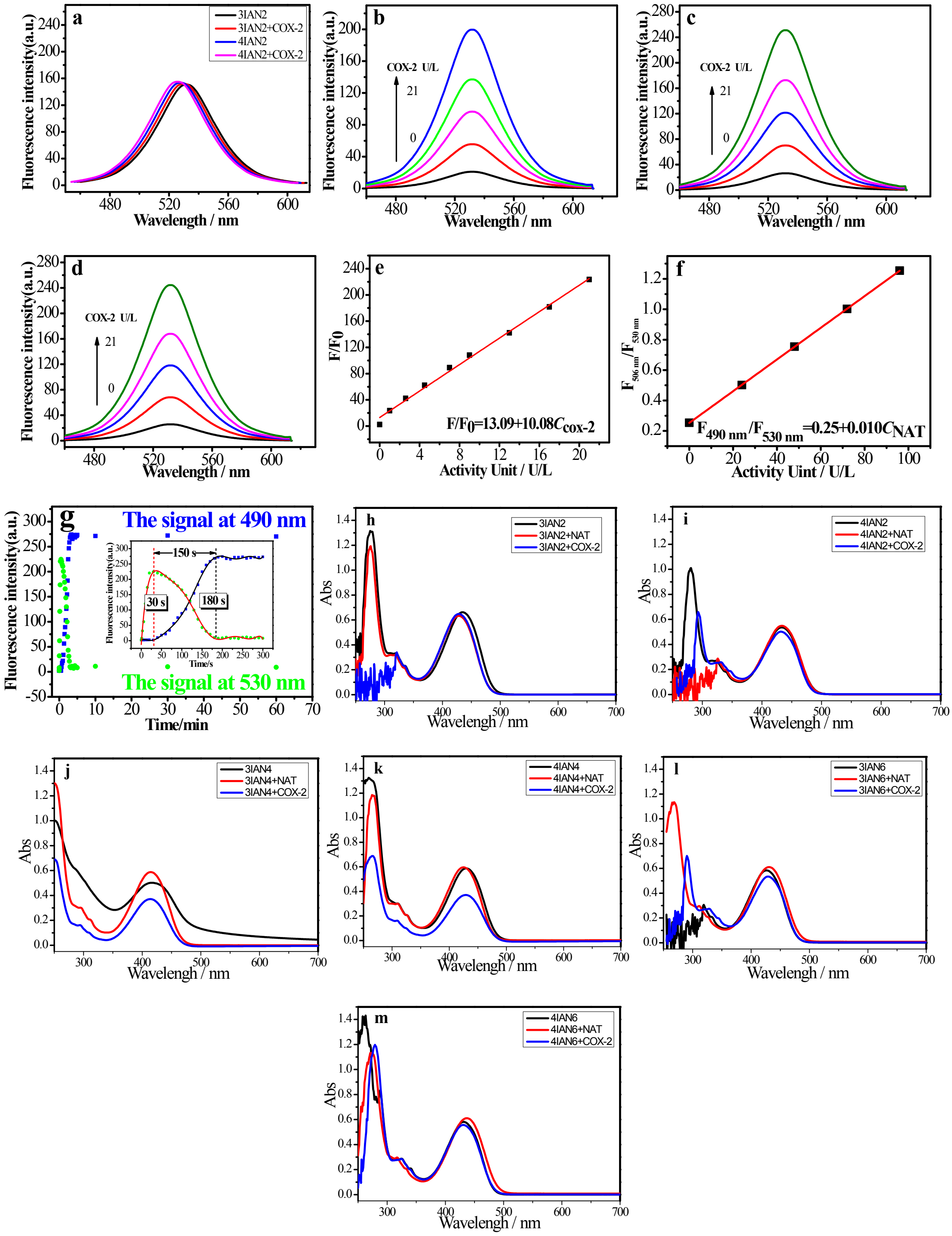

Fig. S1 a. Emission spectra of **3IAN2** (3.0 μM) and **4IAN2** (3.0 μM) with COX-2 (21U/L) in buffer at 25°C. Emission spectra of **3IAN6** (b, 3.0 μM), **4IAN4** (c, 3.0 μM) and **4IAN6** (d, 3.0 μM) with COX-2 (0-21U/L) in buffer at 25°C. e. The linear fluorescence change between **3IAN4** (3.0 μM) and the different concentration of COX-2 (0-21U/L). f. the linear fluorescence change of **3IAN4** (3.0 μM) with the increase of concentration of NAT (0-96 U/L) after adding COX-2 (37.5 U/L) early. g. The time course plot of the fluorescence responses of **3IAN4** (3.0 μM) at 530 nm and 490 nm under 21 U/L of COX-2 and 96 U/L of NAT. The absorption spectra of **3IAN2** (h, 30 μM), **4IAN2** (i, 30 μM), **3IAN4** (j, 30 μM), **4IAN4** (k, 30 μM), **3IAN6** (l, 30 μM) and **4IAN6** (m, 30 μM) with COX-2 (30 U/L) and NAT (100 U/L) in buffer at 25°C.

5. The theoretical calculation data

Table S2 The data about the frontier molecular orbital (FMO) energies

Name	Energy/ev	ΔE (Unfolded–Folded)	Name	Energy	ΔE (Unfolded–Folded)
**3IAN2-Unfolded**	-65509.9932	–2.9 kal/mol	**3IAO2-Unfolded**	-65509.9841	–2.6 kal/mol
**3IAN2-Folded**	-65509.8672		**3IAO2-Folded**	-65509.8732	
**3IAN4-Unfolded**	-65510.8866	8.3 kal/mol	**3IAO4-Unfolded**	-65510.9274	7.1 kal/mol
**3IAN4-Folded**	-65511.2462		**3IAO4-Folded**	-65511.2347	
**3IAN6-Unfolded**	-65512.9017	10.9 kal/mol	**3IAO6-Unfolded**	-65512.8913	9.2 kal/mol
**3IAN6-Folded**	-65513.3721		**3IAO6-Folded**	-65513.2913	

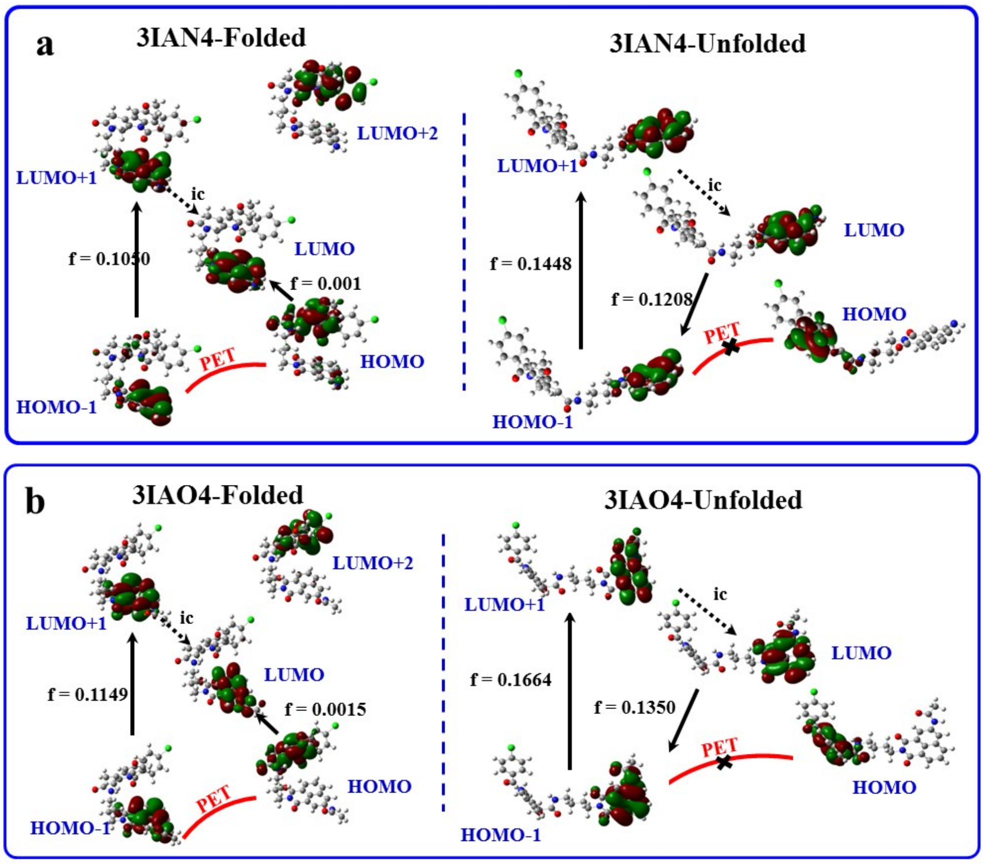

Fig. S2 (a) Structural optimization and frontier molecular orbital (FMO) of **3IAN4-Folded** and **3IAN4-Unfolded** calculated with time-dependent density functional theory using Gaussian 16. (b) Structural optimization and frontier molecular orbital (FMO) of **3IAO4-Folded** and **3IAO4-Unfolded** calculated with time-dependent density functional theory using Gaussian 16.

6. The response to NAT

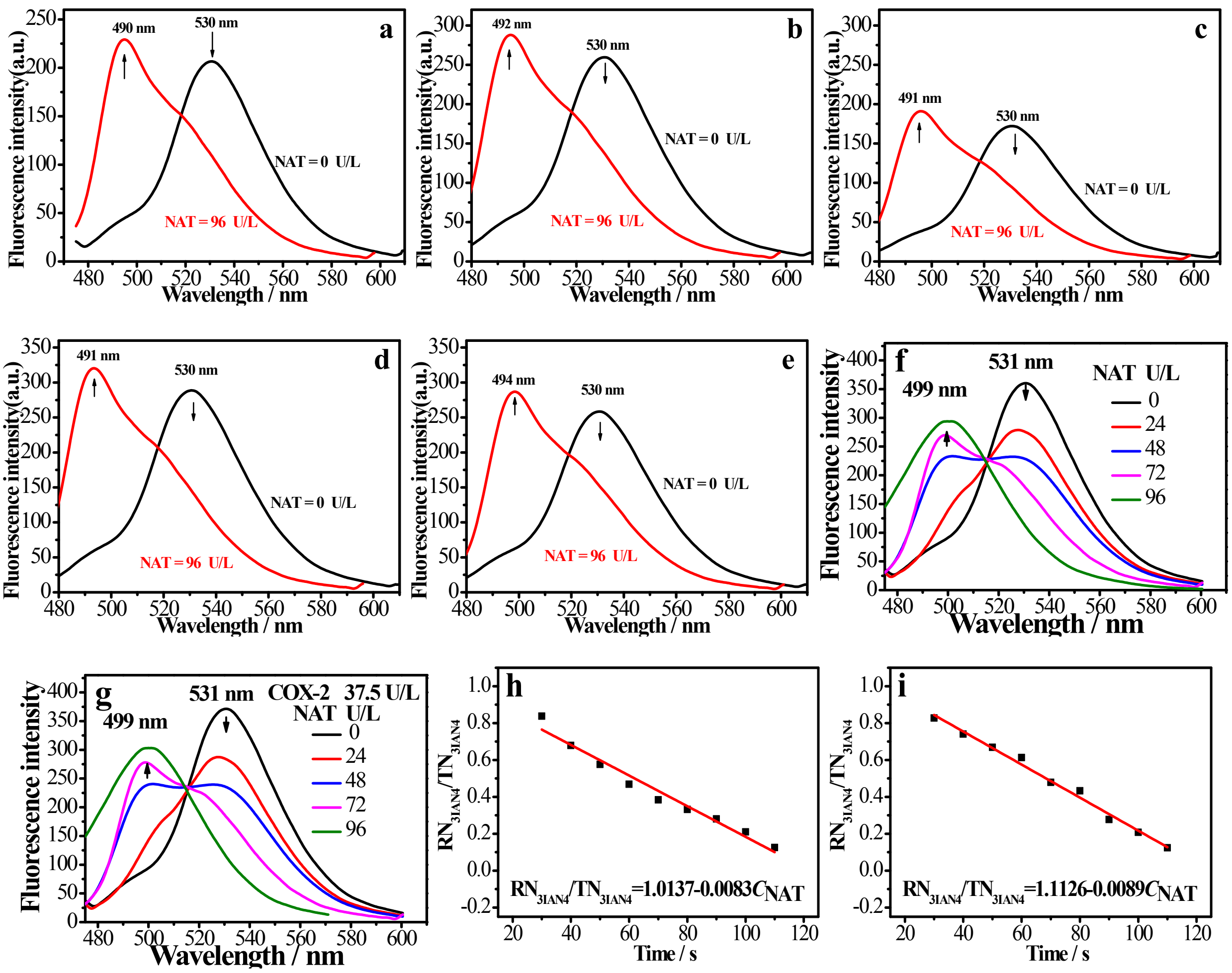

Fig. S3 Emission spectra of **3IAN2** (a, 3.0 μM), **3IAN6** (b, 3.0 μM), **4IAN2** (c, 3.0 μM), **4IAN4** (d, 3.0 μM) and **4IAN6** (e, 3.0 μM) with NAT (0-96 U/L) in the presence of acetyl-CoA after adding COX-2 (21 U/L) early. The emission spectra of the precursor of **3IAN4** (3.0 μM) with NAT (0-96 U/L) in the presence of acetyl-CoA (g and f). 37.5 U/L of COX-2 was first added into solution (f), which ensured that **3IAN4** (3.0 μM) fully reacts with NAT. The reaction kinetics of **3IAN4** (3.0 μM) and NAT in the presence (h) and absence of COX-2 (i).

7. The reproducibility and selectivity

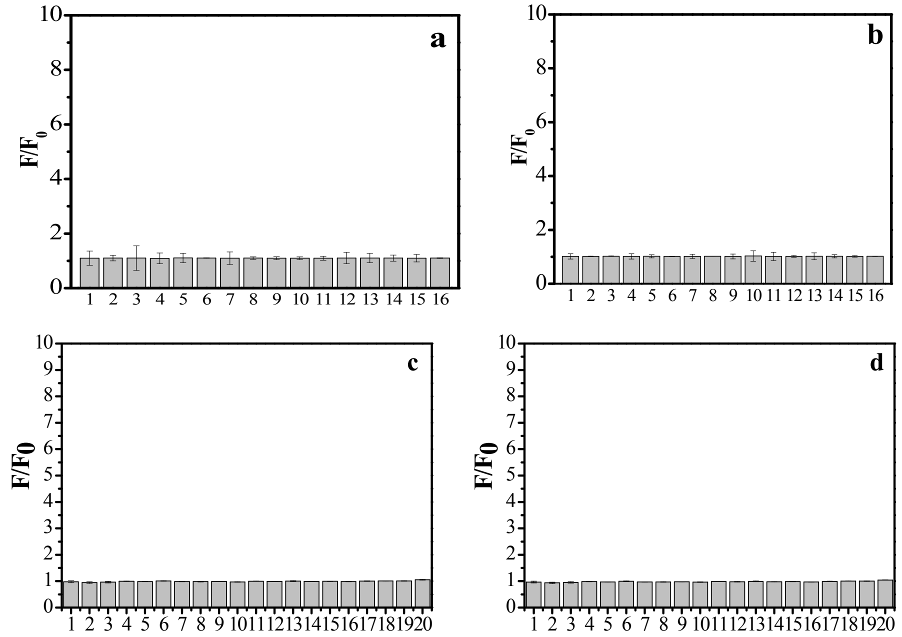

Fig. S4 Ion interferent for **3IAN4** (a, 3.0 μM) and **3IAO4** (b, 3.0 μM): 1, NaCl; 2, $Pb(NO_3)_2$; 3, Na_2CO_3; 4, $CuSO_4$; 5, $Al(NO_3)_3$; 6, $MaSO_4$; 7, $NiCl_2{\cdot}6H_2O$; 8, Li_2CO_3; 9. KH_2PO_4; 10, K_2HPO_4; 11, $CrCl_3{\cdot}6H_2O$; 12, $ZnSO_4$; 13, $FeCl_3$; 14, $HgCl_2$; 15, $AgNO_3$; 16, $CaCl_2$. Amino acids and protein interferent for **3IAN4** (c, 3.0 μM) and **3IAO4** (d, 3.0 μM): 1. Gly-DL-Phe; 2. Lys; 3. DL-Thr; 4. Gin; 5. Cys; 6. Glu; 7. Gly-L-Tyr; 8. DL-Methionine; 9. albumin; 10. Gly; 11. DL-Leu; 12. Ser; 13.L-Glu; 14. DTT; 15. Arginine; 16. Hypoxanthine; 17. L-Asn; 18. Val; 19. DL-Hcy; 20. DNA.

8. The photostability, pH-stability and water solubility of 3IAN4 and 3IAO4

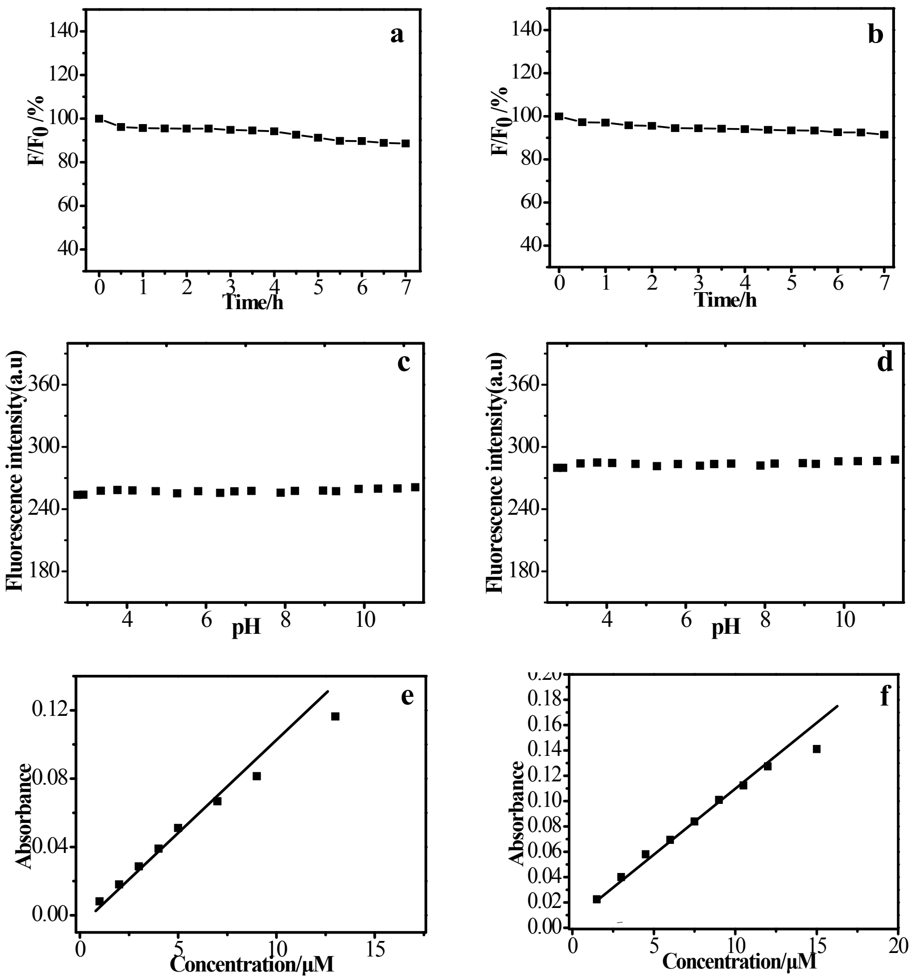

Fig. S5 The photostability (a and b, 3.0 μM), pH-stability (c and d, 3.0 μM) and water solubility (e and f) of **3IAN4** (a, c and e) and **3IAO4** (b, d and f). Photostability, irradiation time: 7.0 h.

9. The cytotoxicity of 3IAN4 and 3IAO4

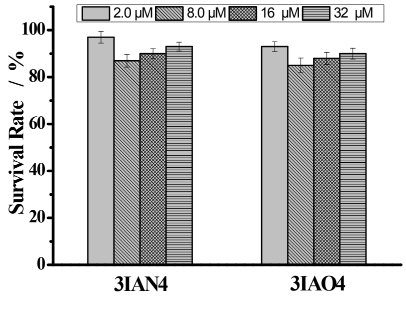

Fig. S6 Cytotoxicity of **3IAN4** and **3IAO4**

10. The solvatochromism of 3IAN4 and 3IAO4.

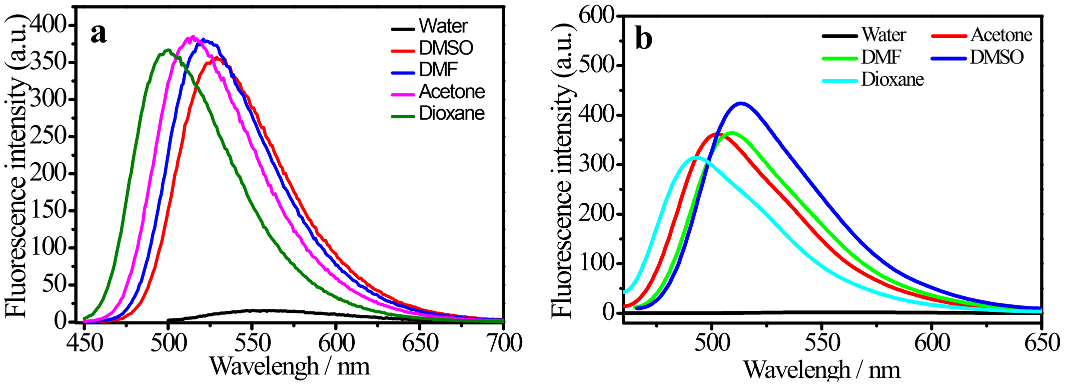

Fig. S7 The solvatochromism of **3IAN4** (a, 3.0 μM) and **3IAO4** (b, 3.0 μM).

Naphthalimide chromophores are solvatochromic dyes. The solvatochromism of 3IAN4 and 3IAO4 at least should be explored. The results indicated that there were the significant solvatochromic effects. But, they all have almost no fluorescence in water.

11. The binding affinities and binding free energies

Table S3 The binding affinities of 3IAN4 and 3IAO4 to COX-2

name	Grid Score (kcal/mol)	Grid_vdw (kcal/mol)	Grid_es (kcal/mol)
3IAN4	-56.33	-56.58	0.25
3IAO4	-53.73	-54.32	0.59

The binding affinities of 3IAN4 and 3IAO4 to COX-2 (PDB ID: 3NT1) obtained by molecular docking were added into the revised manuscript. The binding affinities of 3IAN4 to COX-2 was -56.33 kcal/mol, and the binding affinities of 3IAO4 to COX-2 was -53.73 kcal/mol.

Table S4 The binding free energies of 3IAN4 and NAT in the presence and absence of COX-2.

	Average (kcal/mol)	Std. Dev.	Std. Err. of Mean
In the presence of COX-2	-47.89	4.00	0.57
In the absence of COX-2	-42.54	4.40	0.62

The molecular dynamics showed the binding free energies (Table S4) of 3IAN4 and NAT (PDB ID: 2PQT) in the presence and absence of COX-2 (PDB ID: 3NT1) were -47.89 kcal/mol and -42.54 kcal/mol, respectively. The data indicated whether or not COX-2 exists does not affect 3IAN4 reacts with NAT.

Table S5 The binding affinities of 3IAN4 and 3IAO4 to albumin

name	Grid Score (kcal/mol)	Grid_vdw (kcal/mol)	Grid_es (kcal/mol)
3IAN4	-19.60	-17.86	-1.74
3IAO4	-18.76	-17.18	-1.58

The molecular docking showed the binding affinities (Table S4) of 3IAN4 and 3IAO4 to albumin (PDB ID: 1BM0) were -19.60 kcal/mol and -18.76 kcal/mol, respectively. The data indicated 3IAN4 and 3IAO4 can not bind with albumin.

The binding affinity of 3IAN4 to COX-2 provided as Kd: The fluorescence intensity at 530 nm of 3IAN4 (0, 1, 2, 3, 4, 5, 7, 9, 13, 17, 25, 35, 45 μM) were detected with7 U/L of COX-2. The excitation wavelength was 430 nm. The fluorescence intensity (Y-axis) and the amount of **3IAN4** (X-axis) were then evaluated using graphpad software. Using this data the Kd of 3IAN4 with COX-2 was determined.

The binding affinity of 3IAN4 to NAT provided as Kd: Because 3IAN4 is not fluorescent with NAT, the product (3IAO4-Folded) of 3IAN4 with NAT was detected using HPLC-MS, and the integral area of the HPLC peak was analyzed. 3IAN4 (0, 1, 2, 3, 4, 5, 7, 9, 13, 17, 25, 35, 45 μM) were reacted with NAT (25 U/L). The reaction time was 30 min. The protein was precipitated using acetonitrile that was stored at 4 ^{o}C and centrifuged at 20000g for 20 minutes. The supernatant was evaluated by mass spectrometry to determine if acetylated product exists and the integral area of the HPLC peak used to determine the amount of the product after acetylation. Finally, the integral area (Y-axis) and the amount of **3IAN4** (X-axis) were calculated using graphpad software. Using this data, the Kd of 3IAN4 with NAT was determined.

Figure for Kd of 3IAN4 with COX-2 and NAT:

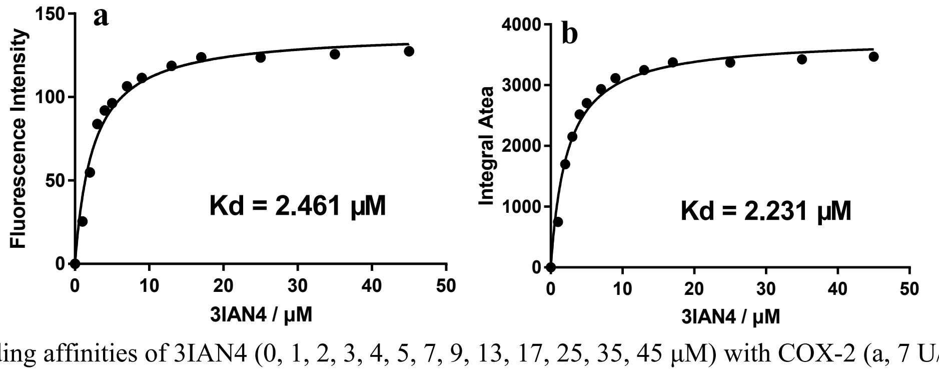

Fig. S8 The binding affinities of 3IAN4 (0, 1, 2, 3, 4, 5, 7, 9, 13, 17, 25, 35, 45 μM) with COX-2 (a, 7 U/L) and NAT (b, 25 U/L) as Kd. a. The reaction time is 10 min. b. The reaction time is 30 min.

12. Imaging and Visualization of tumour resection

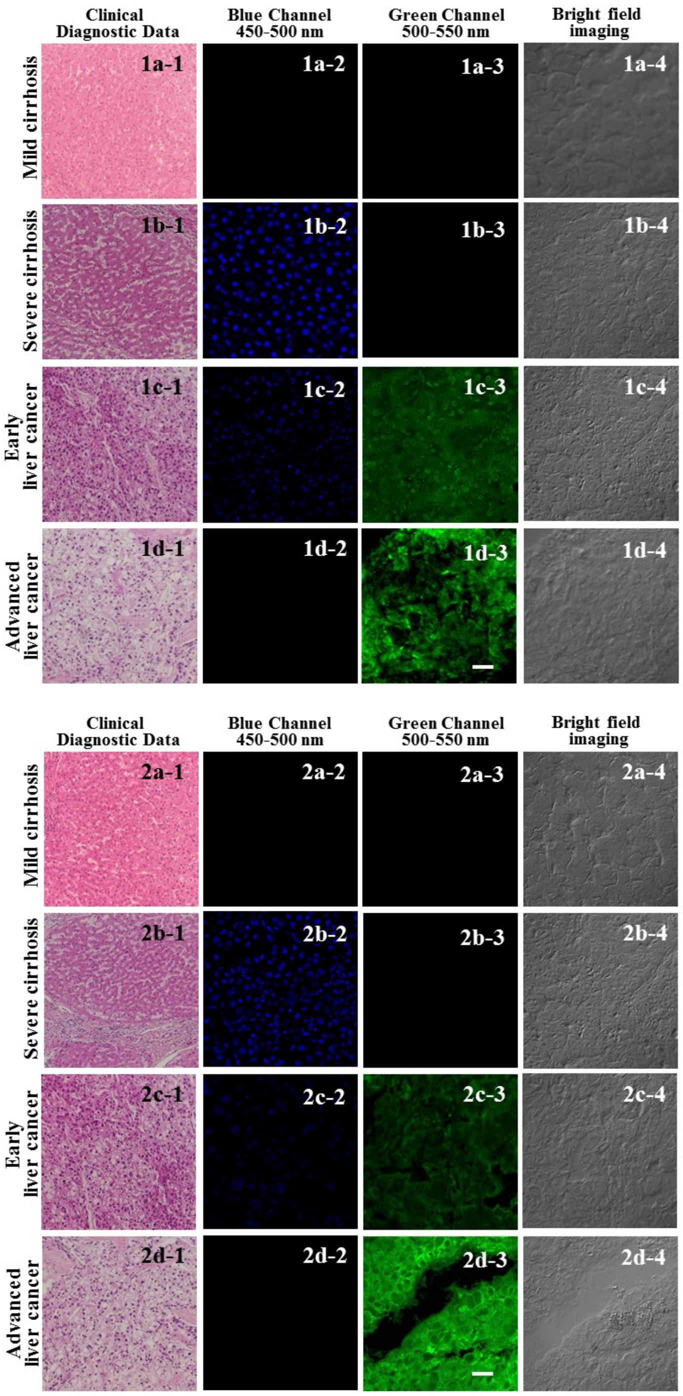

Fig. S9 The signal changes of **3IAN4** (10.0 μM) as a molecular-logic gate to differentially monitor cirrhosis and hepatoma. (1a and 2a) mild cirrhosis. (1b and 2b) severe cirrhosis. (1c and 2c) early liver cancer (1d and 2d) advanced liver cancer. (1a-1 and 2a-1) to (1d-1 and 2d-1), hematoxylin-eosin staining data. (1a-2 and 2a-2) to (1d-2 and 2d-2), (1a-3 and 2a-3) to (1d-3 and 2d-3) and (1a-4 and 2a-4) to (1d-4 and 2d-4) are microscopic imaging. (1a-2 and 2a-2) to (1d-2 and 2d-2), Blue channel: excitation wavelength = 405 nm, scan range = 450-500 nm; (1a-3 and 2a -3) to (1d-3 and 2d-3), Green channel: excitation wavelength = 488 nm, scan range = 500-550 nm; (1a-4 and 2a-4) to (1d-4 and 2d-4), Bright field imaging.

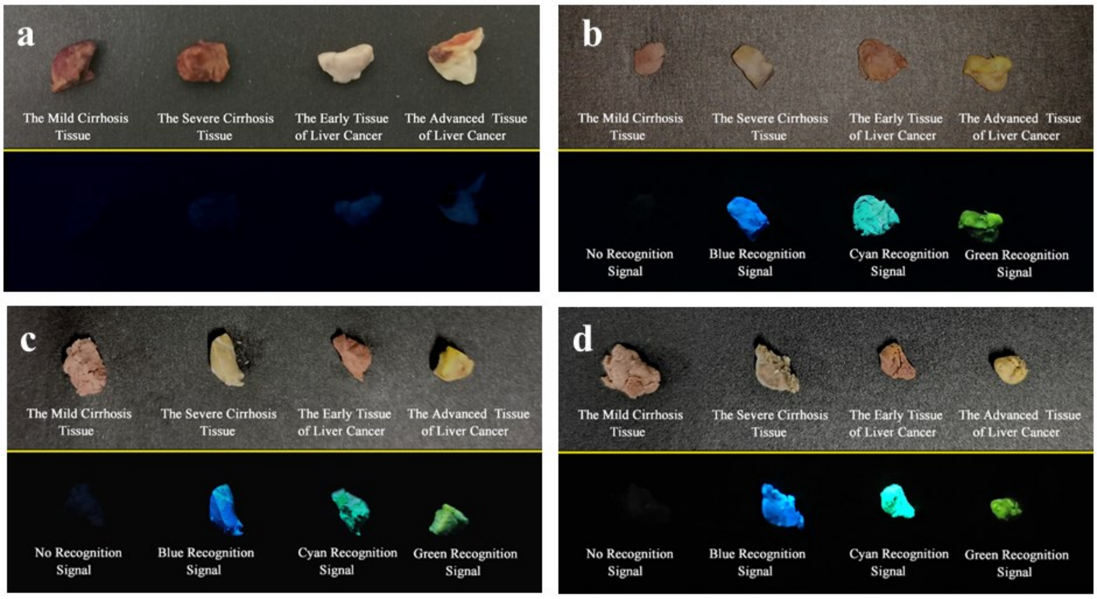

Fig.S10 Visualization of tumour resection untreated with porbe by the naked eye under ultraviolet illumination. (a) blank group; (b), (c) and (d) experimental group, three sets of parallel result.

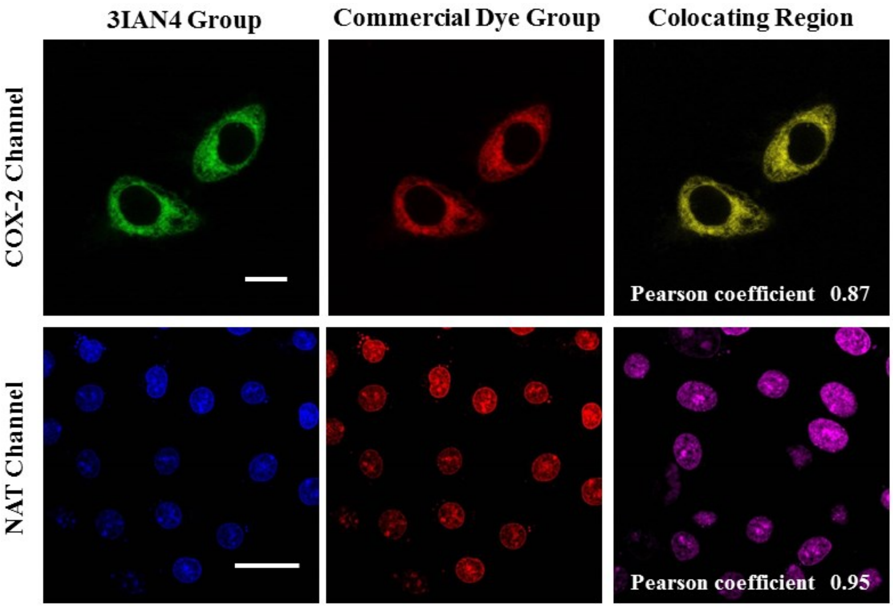

Fig. S11 The coclocalization experiments. 3IAN4 (3.0 μM, COX-2 Channel: the activity of NAT was completely inhibited; NAT Channel: the activity of COX-2 was completely inhibited.), ER-Tracker Red (10.0 μM, COX-2 Channel-Commercial dye; Excitation wavelength: 559 nm, it was collected at 600-630 nm), Propidium Iodide (10.0 μM, NAT Channel-Commercial dye; Excitation wavelength: 559 nm, it was collected at 600-630 nm). The images were obtained from replicate experiments (n = 3)

References

S1. Gaussian 16, Revision B.01, M. J. Frisch, G. W. Trucks, H. B. Schlegel, G. E. Scuseria, M. A. Robb, J. R. Cheeseman, G. Scalmani, V. Barone, G. A. Petersson, H. Nakatsuji, X. Li, M. Caricato, A. V. Marenich, J. Bloino, B. G. Janesko, R. Gomperts, B. Mennucci, H. P. Hratchian, J. V. Ortiz, A. F. Izmaylov, J. L. Sonnenberg, D. Williams-Young, F. Ding, F. Lipparini, F. Egidi, J. Goings, B. Peng, A. Petrone, T. Henderson, D. Ranasinghe, V. G. Zakrzewski, J. Gao, N. Rega, G. Zheng, W. Liang, M. Hada, M. Ehara, K. Toyota, R. Fukuda, J. Hasegawa, M. Ishida, T. Nakajima, Y. Honda, O. Kitao, H. Nakai, T. Vreven, K. Throssell, J. A. Montgomery, Jr., J. E. Peralta, F. Ogliaro, M. J. Bearpark, J. J. Heyd, E. N. Brothers, K. N. Kudin, V. N. Staroverov, T. A. Keith, R. Kobayashi, J. Normand, K. Raghavachari, A. P. Rendell, J. C. Burant, S. S. Iyengar, J. Tomasi, M. Cossi, J. M. Millam, M. Klene, C. Adamo, R. Cammi, J. W. Ochterski, R. L. Martin, K. Morokuma, O. Farkas, J. B. Foresman, and D. J. Fox, Gaussian, Inc., Wallingford CT, 2016.

S2. J. D. Chai, M. Head-Gordon, *Phys. Chem. Chem. Phys*, 2008, **10**, 6615–6620.

S3. Y. Takano and K. N. Houk, *J. Chem. Theory Comput.*, 2005, **1**, 70–77.

S4. H. Cao, D. I. Diaz, N. S. DiCesare, J. R. Lakowicz and M. D. Heagy, *Org. Lett.*, 2002, **4**, 1503-1505.

Attached spectra

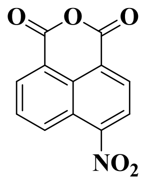

6-nitrobenzo[*de*]isochromene-1,3-dione

^{1}H NMR of A-1

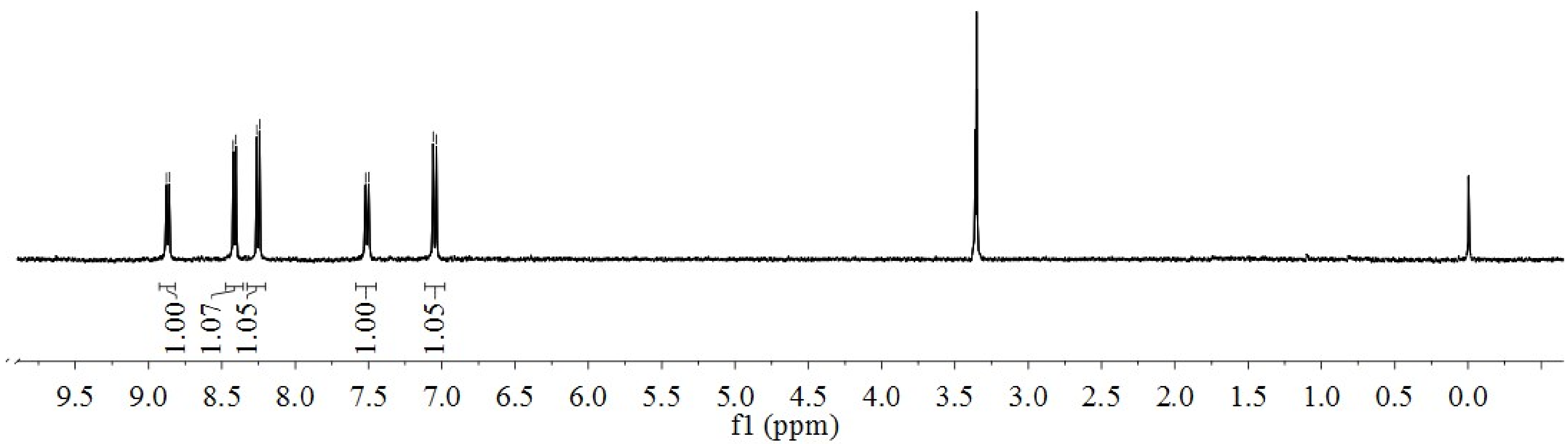

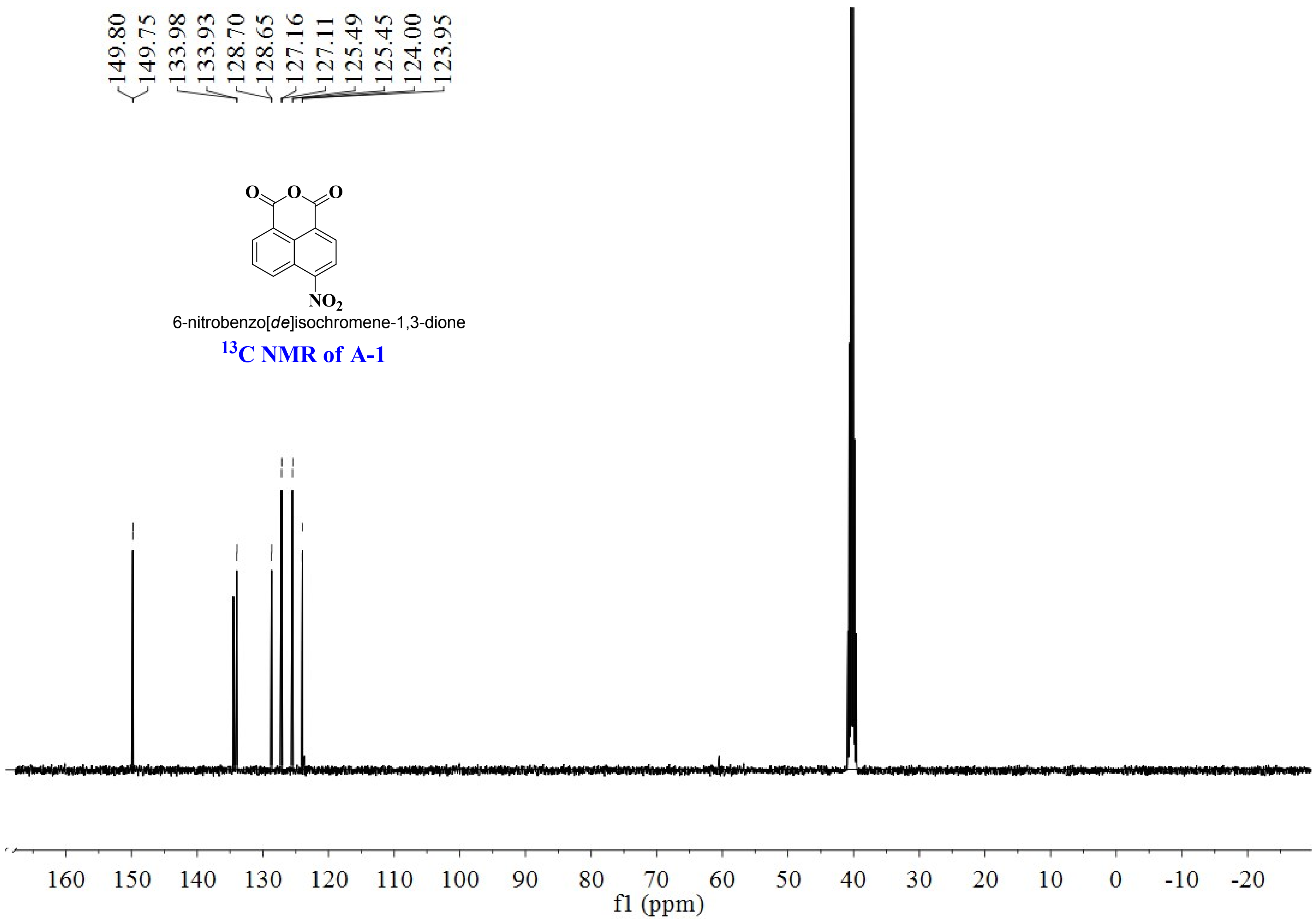
149.80
149.75
133.98
133.93
128.70
128.65
127.16
127.11
125.49
125.45
124.00
123.95
O
O
O
NO2
6-nitrobenzo[de]isochromene-1,3-dione
13C NMR of A-1
160 150 140 130 120 110 100 90 80 70 60 50 40 30 20 10 0 -10 -20
f1 (ppm)

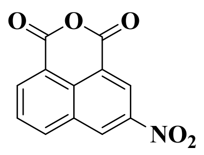

5-nitrobenzo[*de*]isochromene-1,3-dione

^{1}H NMR of A-2

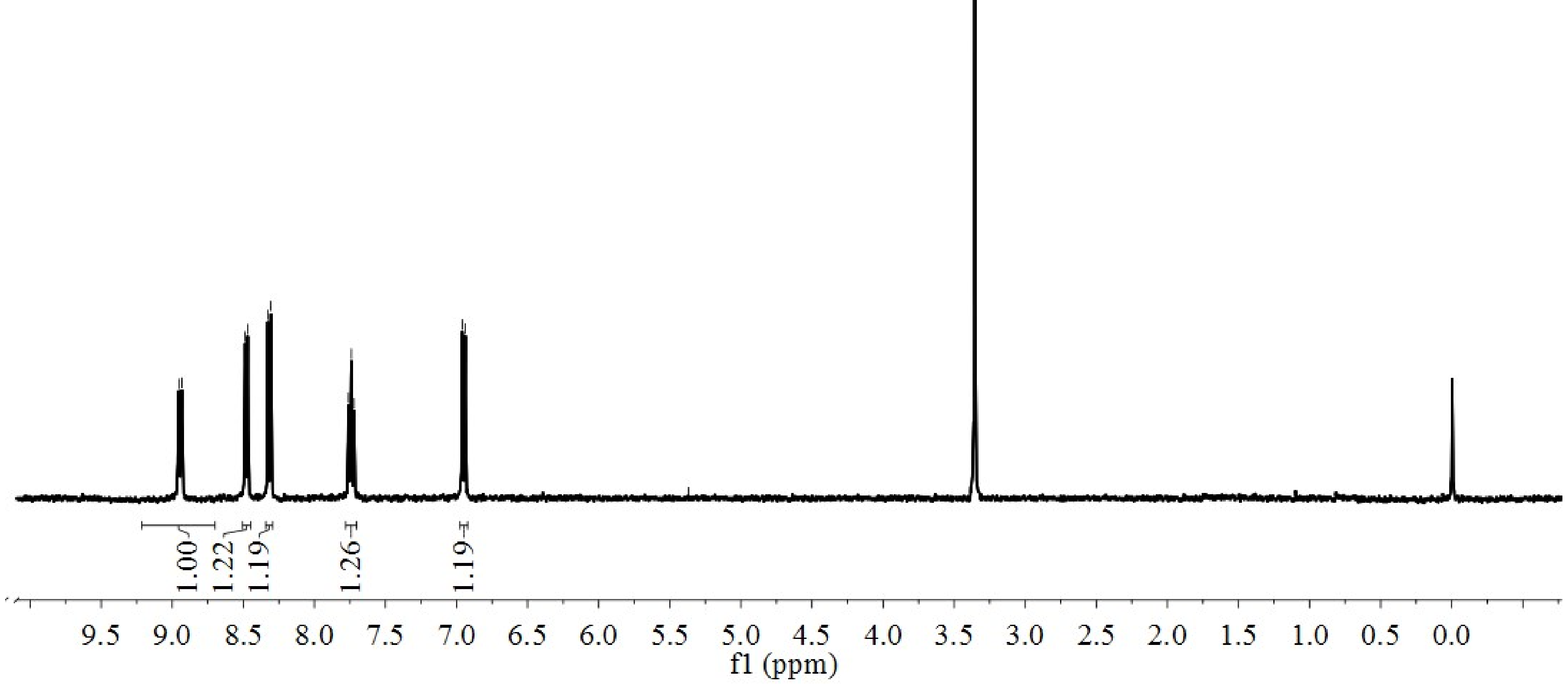

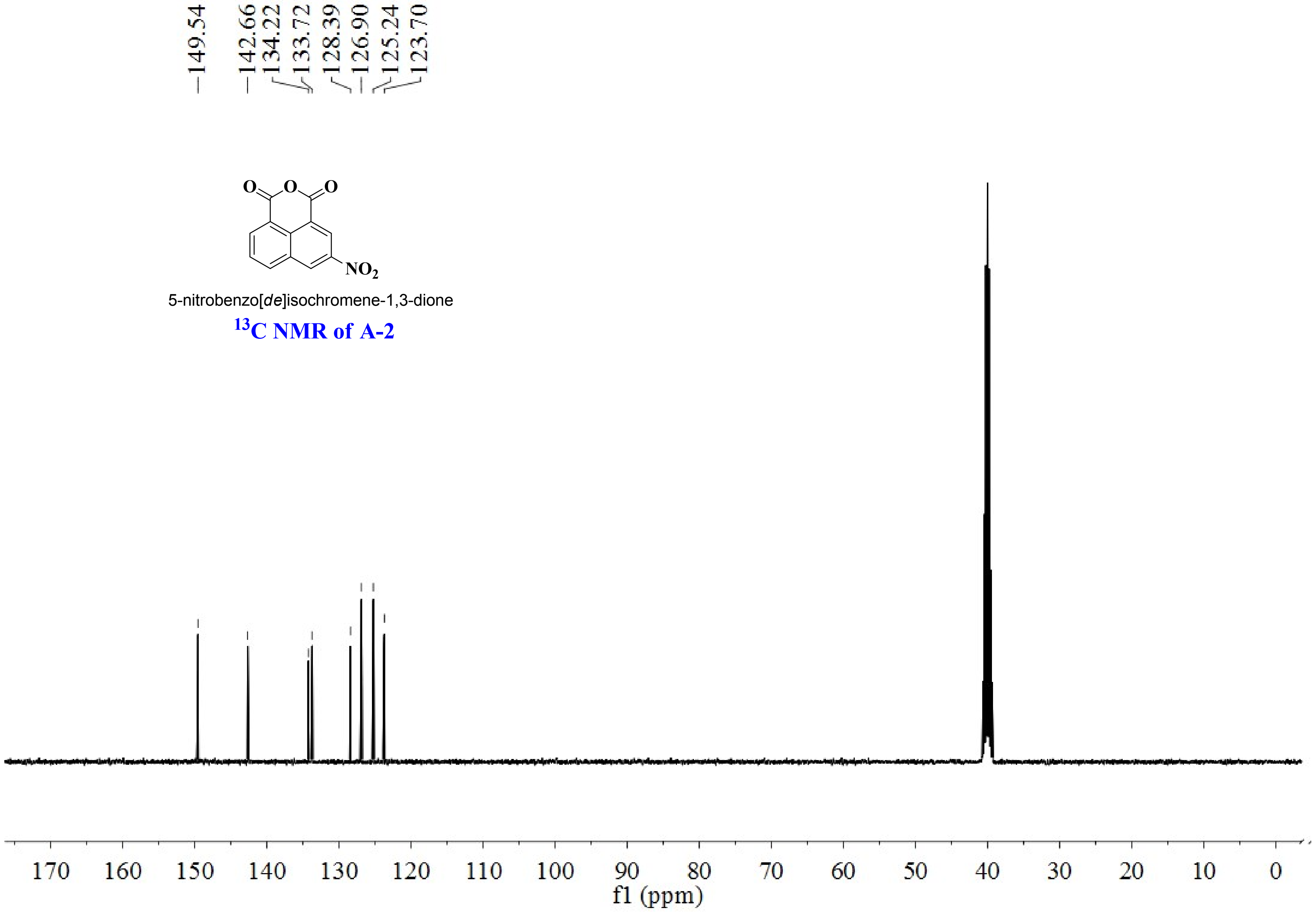

5-nitrobenzo[*de*]isochromene-1,3-dione

^{13}C NMR of A-2

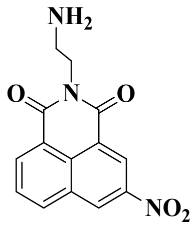

2-(2-aminoethyl)-5-nitro-1*H*-benzo[*de*]isoquinoline-1,3(2*H*)-dione

^{1}H NMR of 3B2

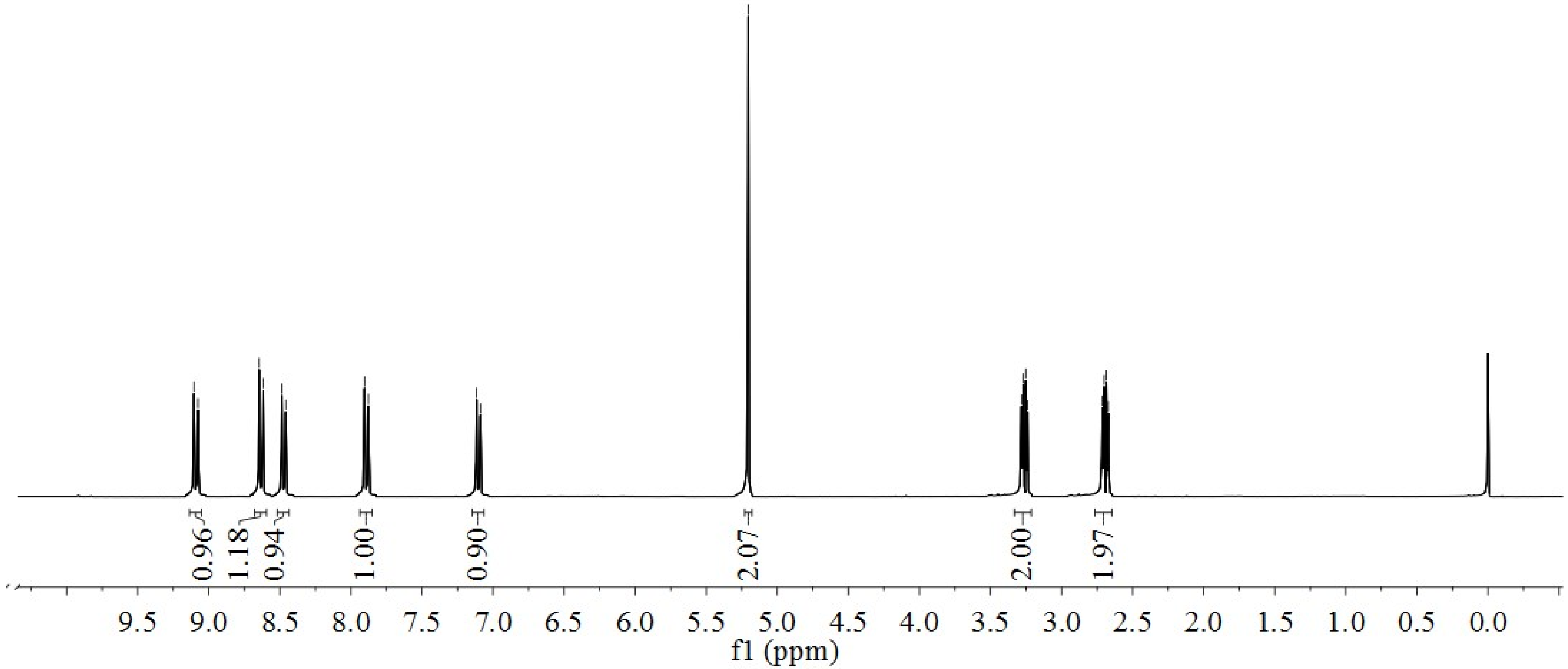

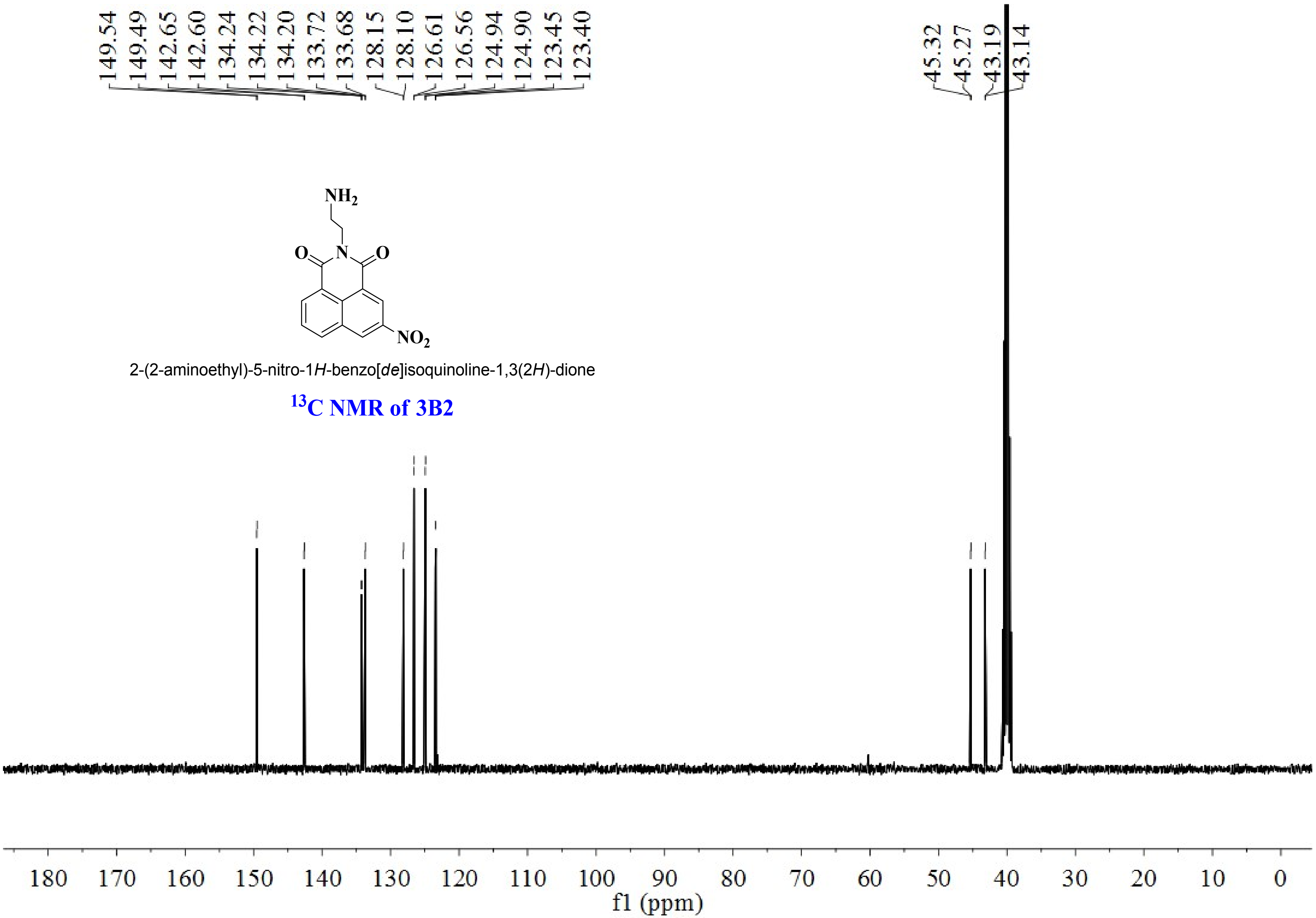

2-(2-aminoethyl)-5-nitro-1*H*-benzo[*de*]isoquinoline-1,3(2*H*)-dione

^{13}C NMR of 3B2

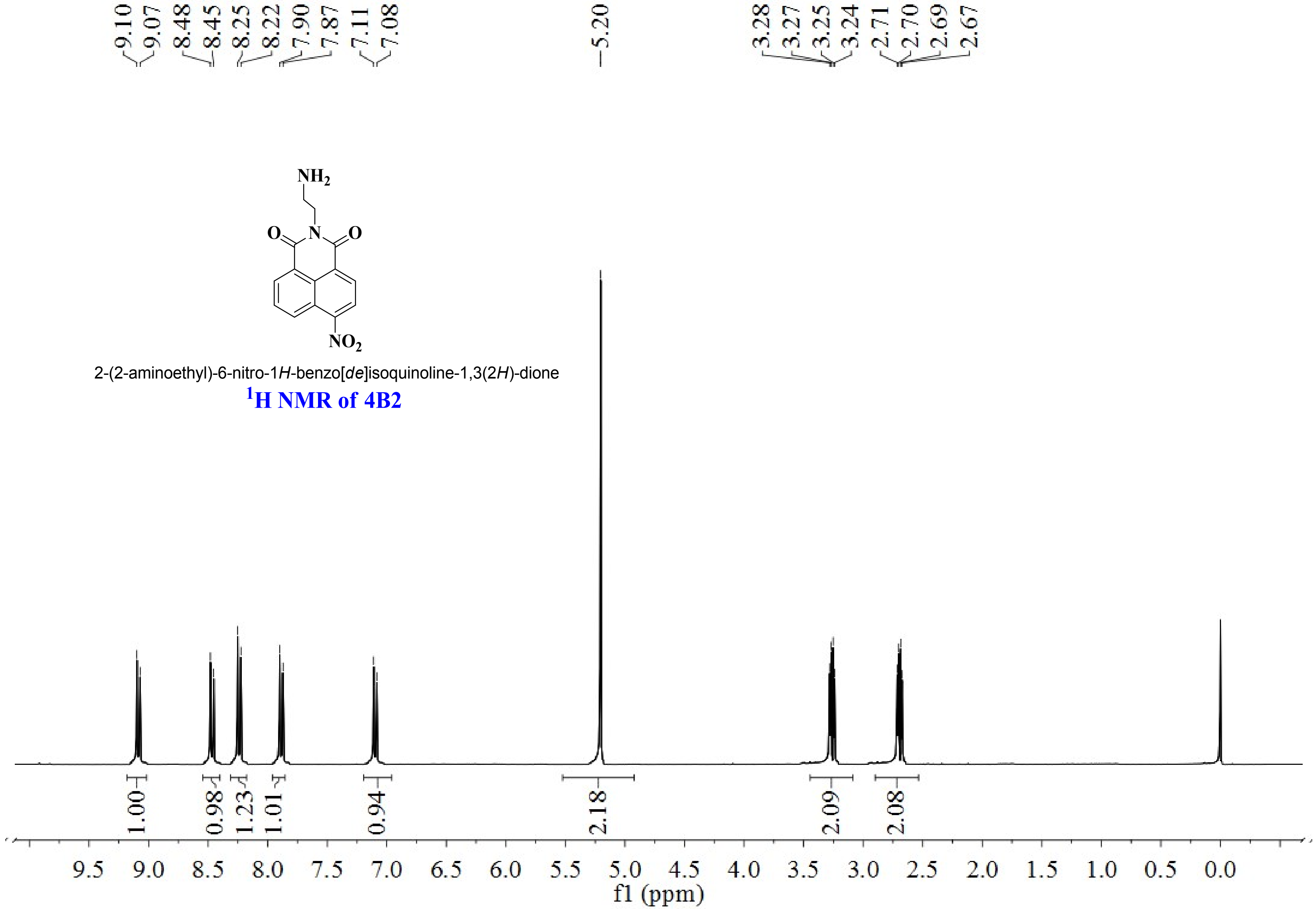
9.10
9.07
8.48
8.45
8.25
8.22
7.90
7.87
7.11
7.08
5.20
3.28
3.27
3.25
3.24
2.71
2.70
2.69
2.67
NH_2
O
N
O
NO_2
2-(2-aminoethyl)-6-nitro-1*H*-benzo[*de*]isoquinoline-1,3(2*H*)-dione
^{1}H NMR of 4B2
1.00
0.98
1.23
1.01
0.94
2.18
2.09
2.08
9.5 9.0 8.5 8.0 7.5 7.0 6.5 6.0 5.5 5.0 4.5 4.0 3.5 3.0 2.5 2.0 1.5 1.0 0.5 0.0
f1 (ppm)

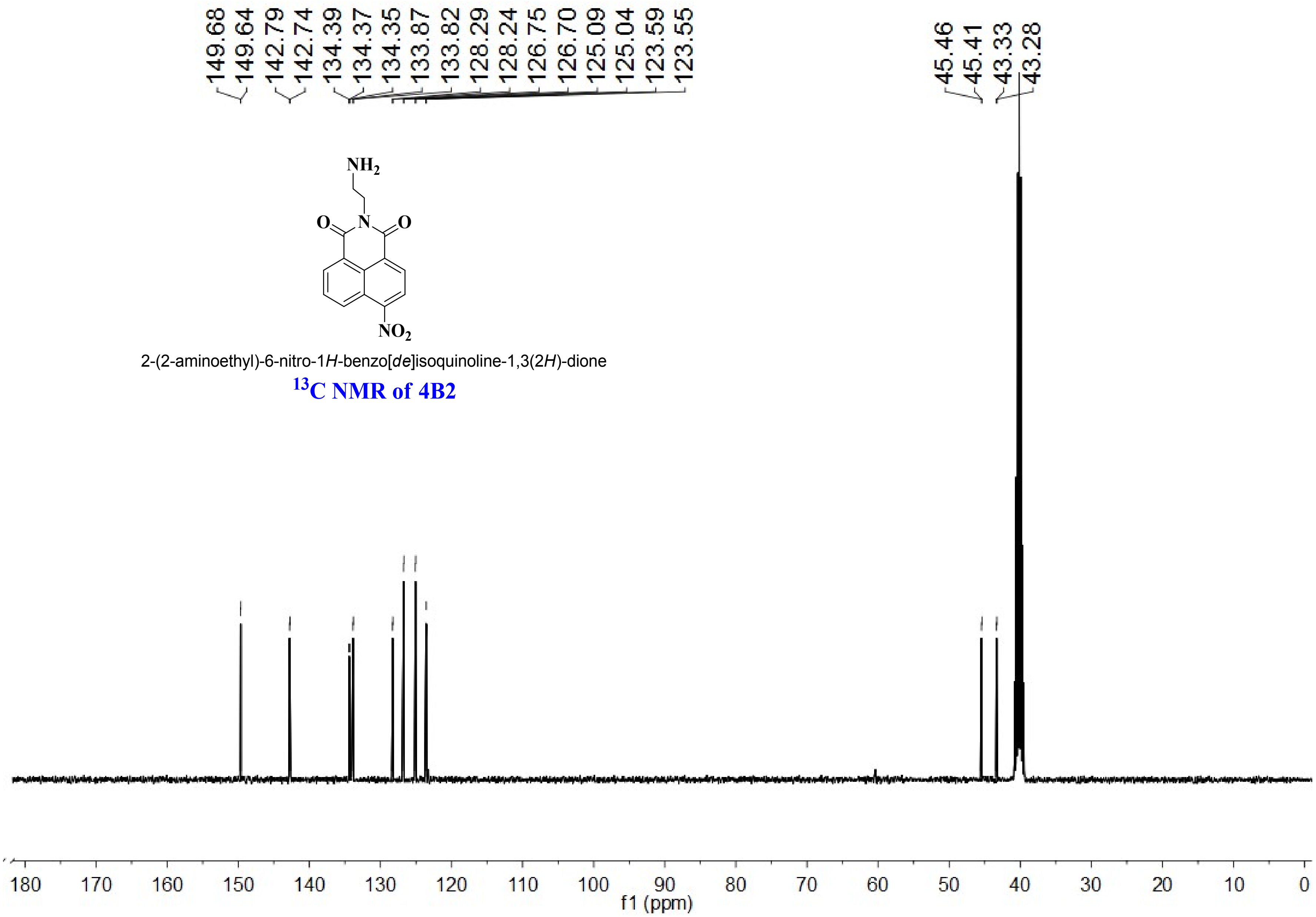

^{13}C NMR of 4B2

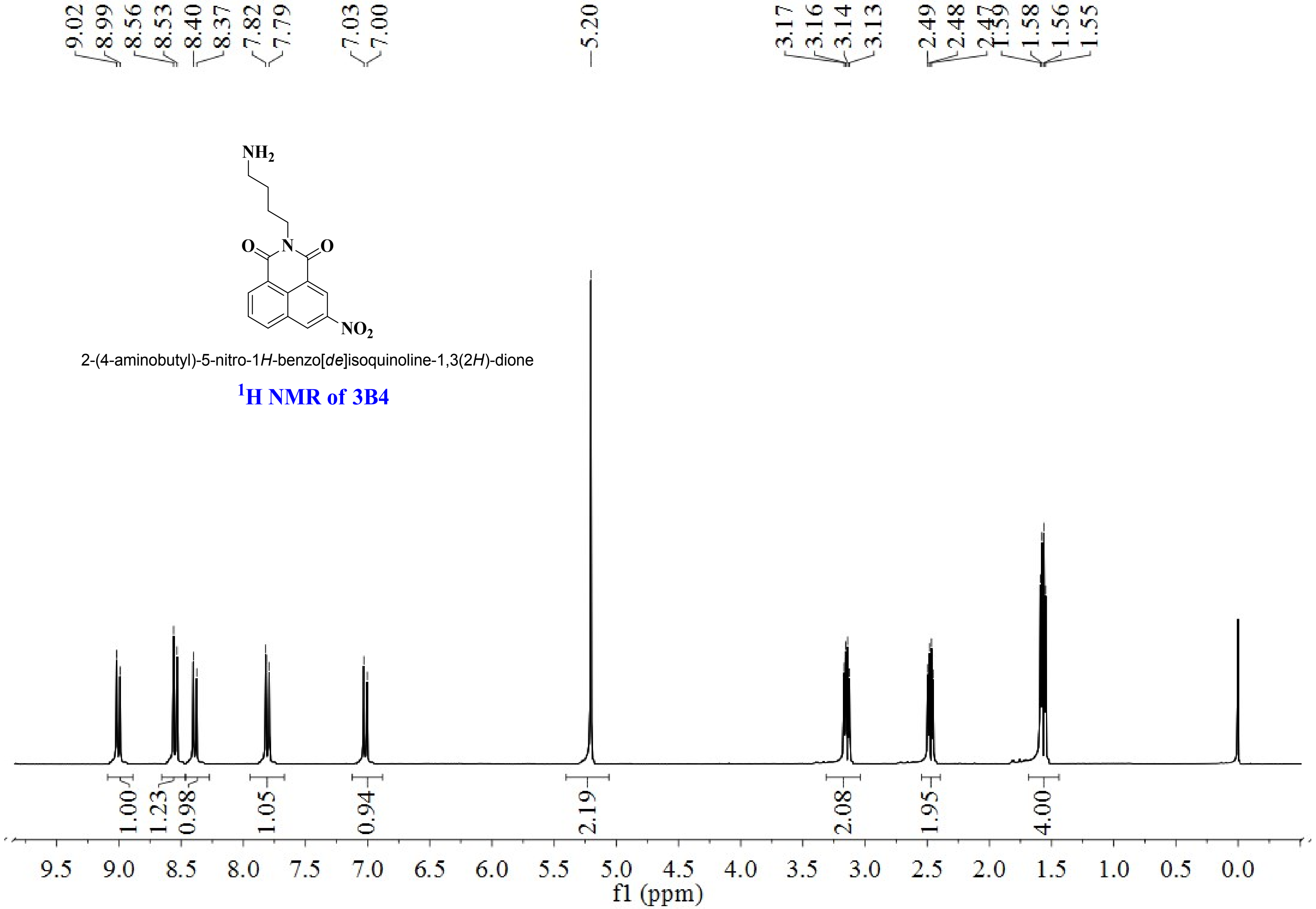

2-(4-aminobutyl)-5-nitro-1*H*-benzo[*de*]isoquinoline-1,3(2*H*)-dione

^{1}H NMR of 3B4

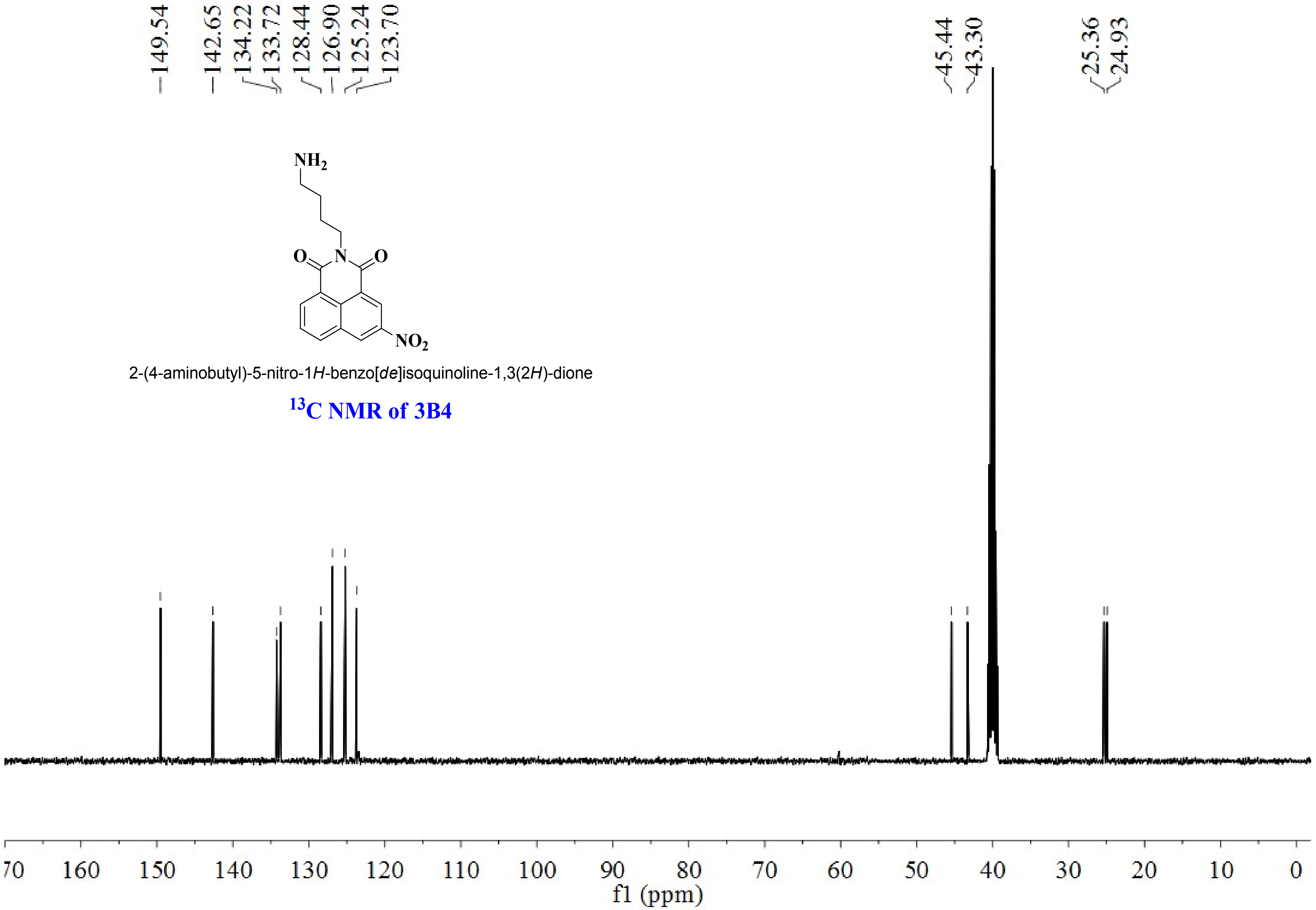

^{13}C NMR of 3B4

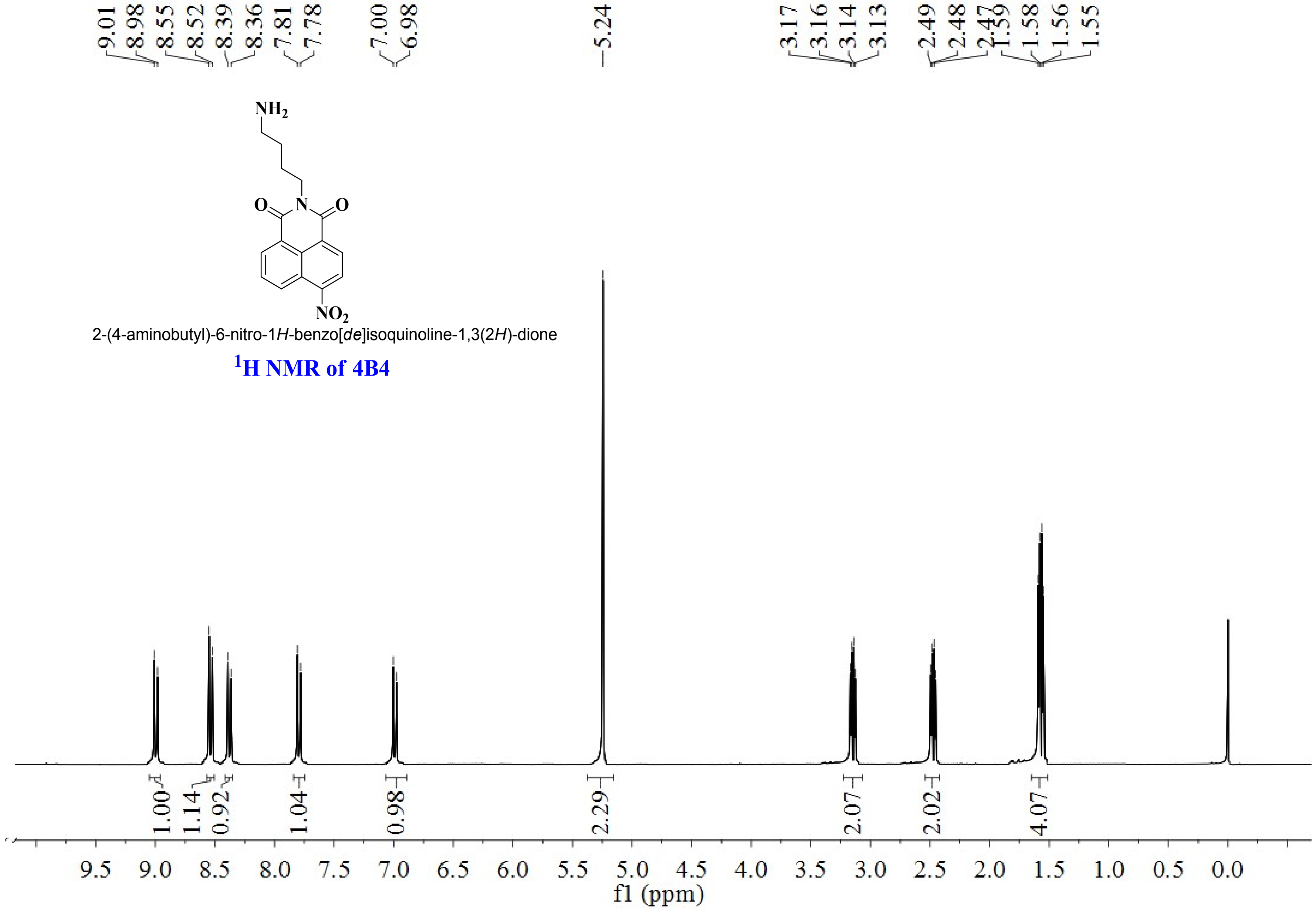
9.01 8.98 8.55 8.52 8.39 8.36 7.81 7.78 7.00 6.98
5.24
3.17 3.16 3.14 3.13 2.49 2.48 2.47 1.59 1.58 1.56 1.55
NH2
O N O
NO2
2-(4-aminobutyl)-6-nitro-1H-benzo[de]isoquinoline-1,3(2H)-dione
1H NMR of 4B4
1.00 1.14 0.92 1.04 0.98 2.29 2.07 2.02 4.07
9.5 9.0 8.5 8.0 7.5 7.0 6.5 6.0 5.5 5.0 4.5 4.0 3.5 3.0 2.5 2.0 1.5 1.0 0.5 0.0
f1 (ppm)

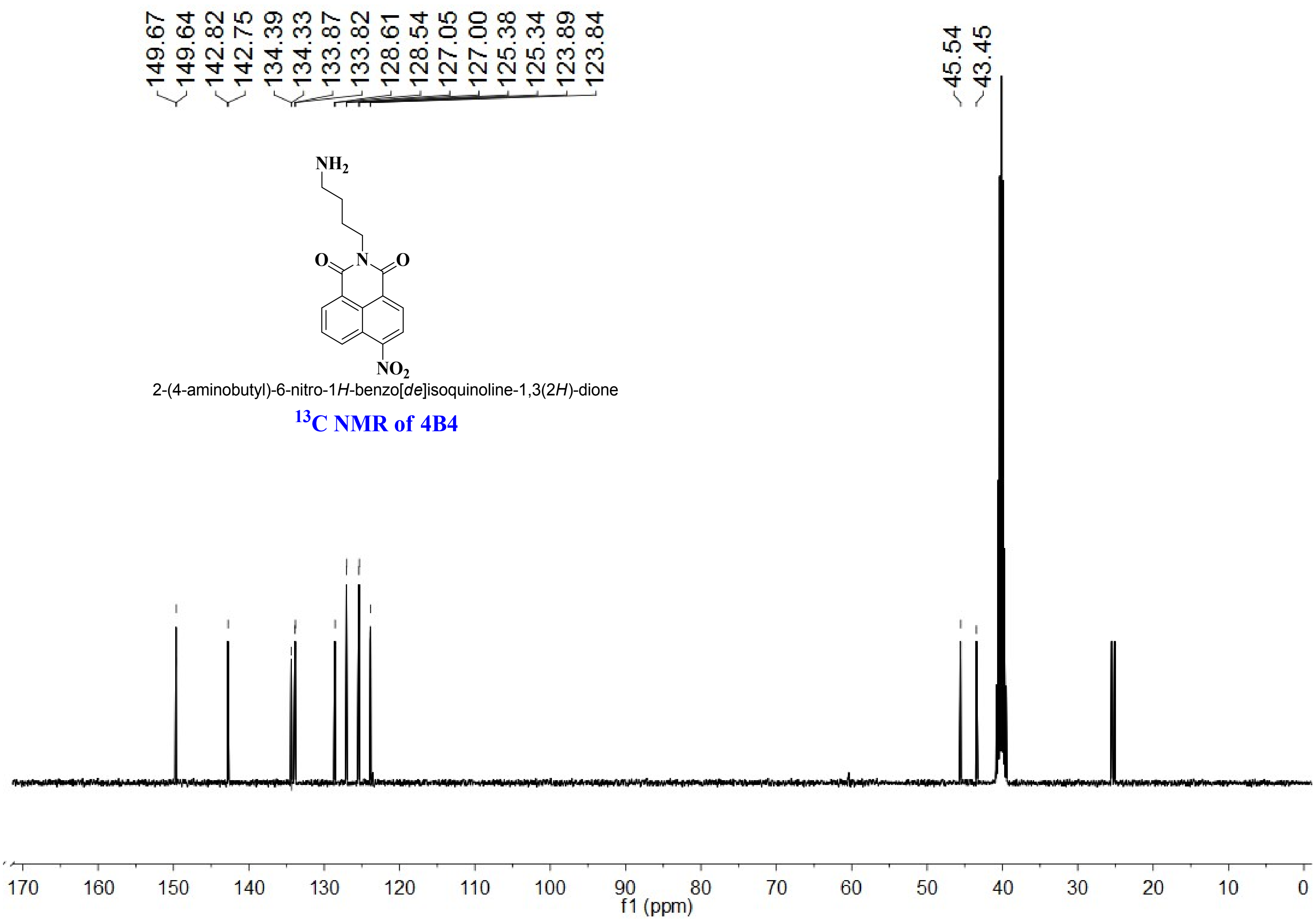

2-(4-aminobutyl)-6-nitro-1*H*-benzo[*de*]isoquinoline-1,3(2*H*)-dione

^{13}C NMR of 4B4

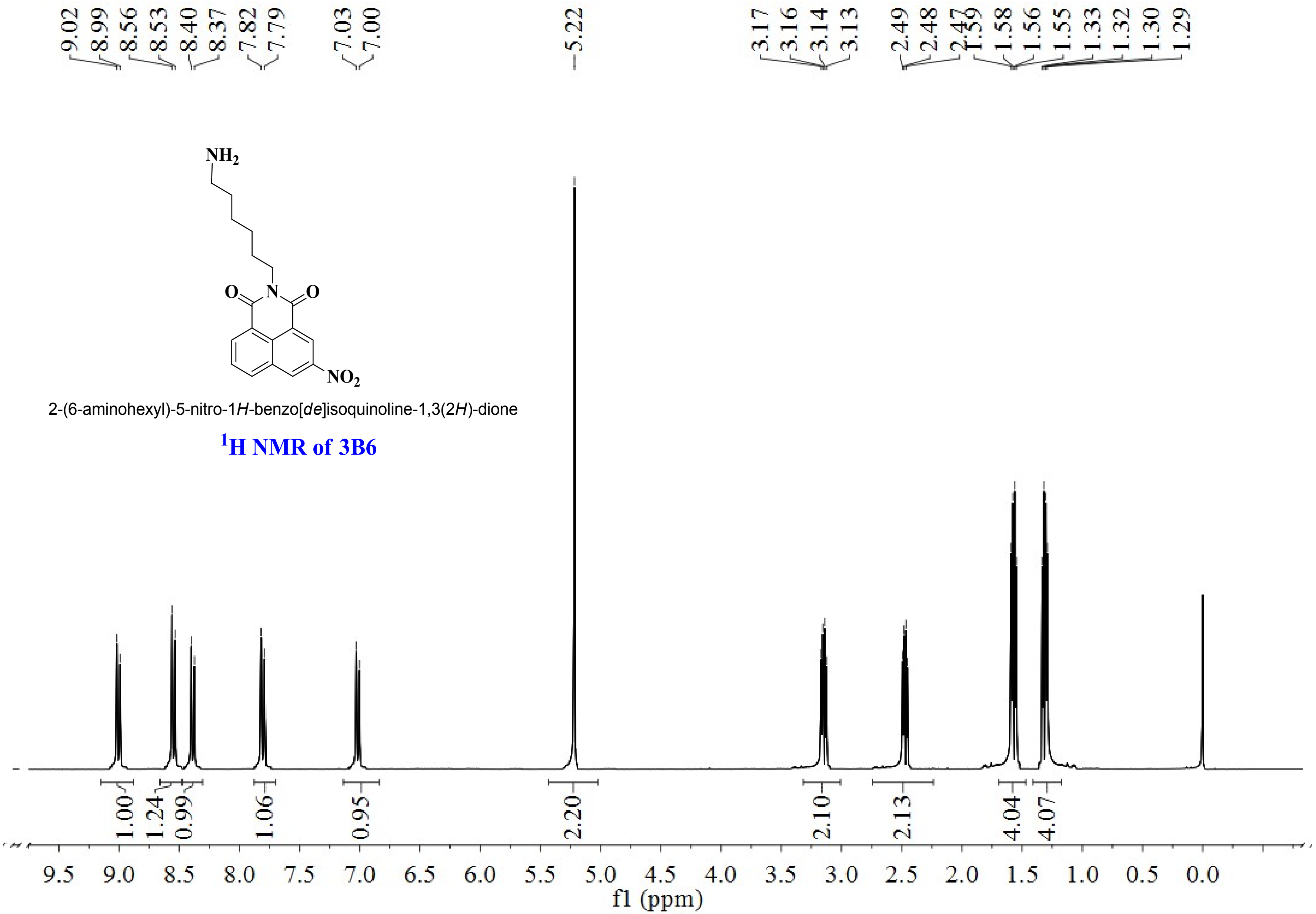

2-(6-aminohexyl)-5-nitro-1*H*-benzo[*de*]isoquinoline-1,3(2*H*)-dione

^{1}H NMR of 3B6

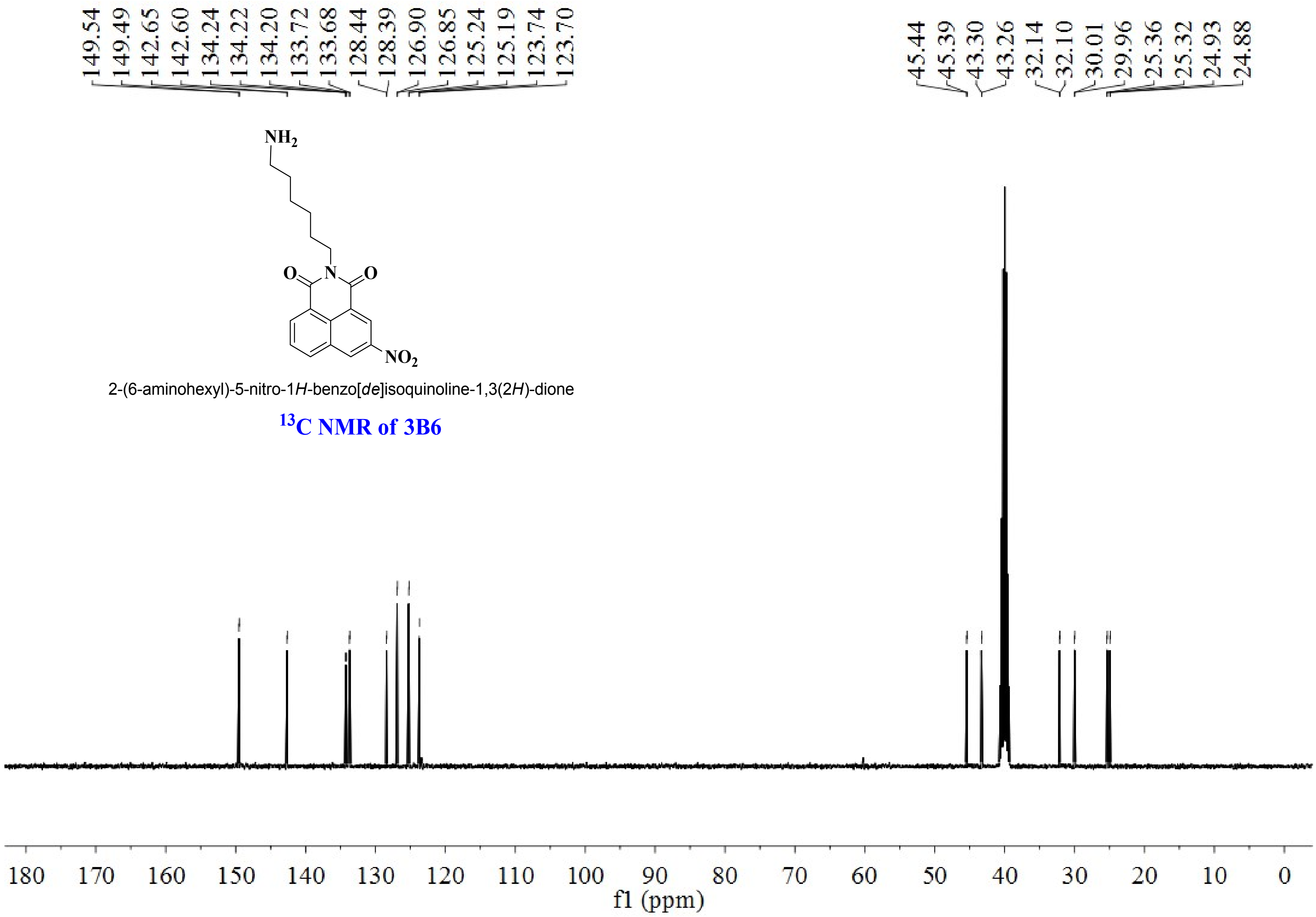

2-(6-aminohexyl)-5-nitro-1*H*-benzo[*de*]isoquinoline-1,3(2*H*)-dione

^{13}C NMR of 3B6

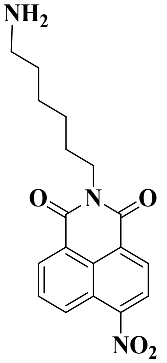

2-(6-aminohexyl)-6-nitro-1*H*-benzo[*de*]isoquinoline-1,3(2*H*)-dione

^{1}H NMR of 4B6

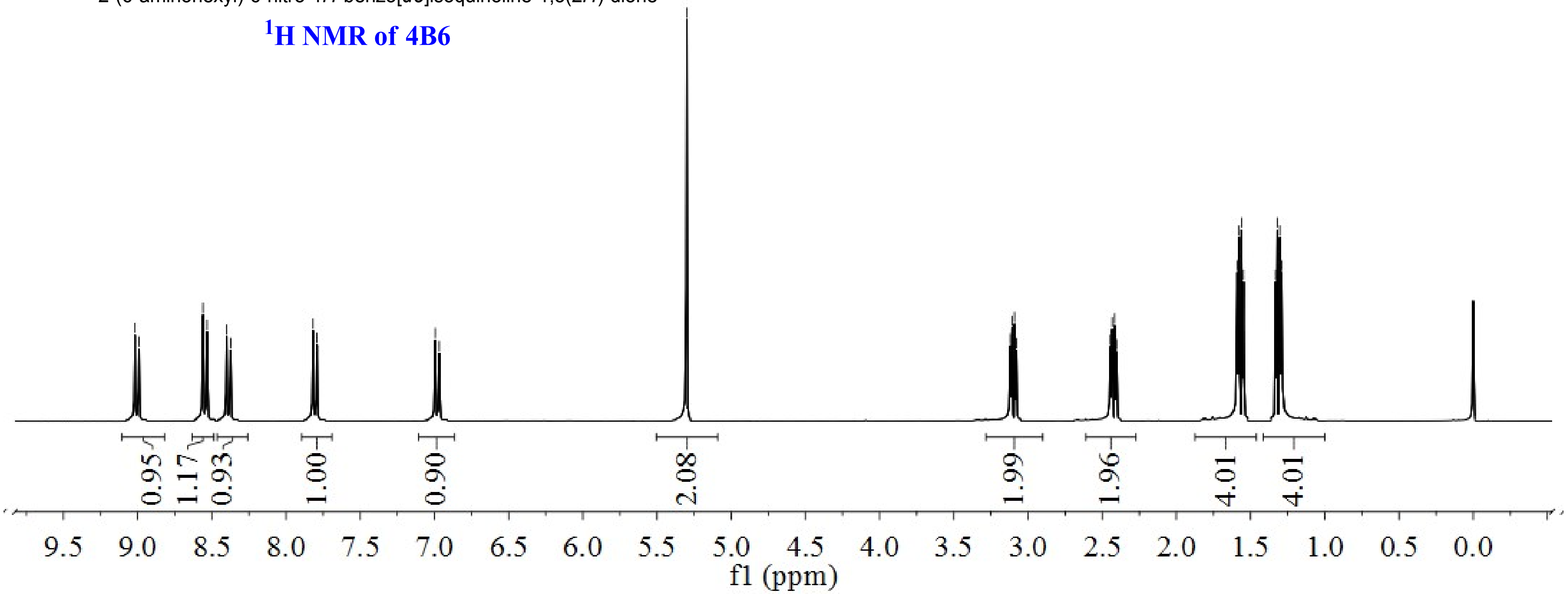

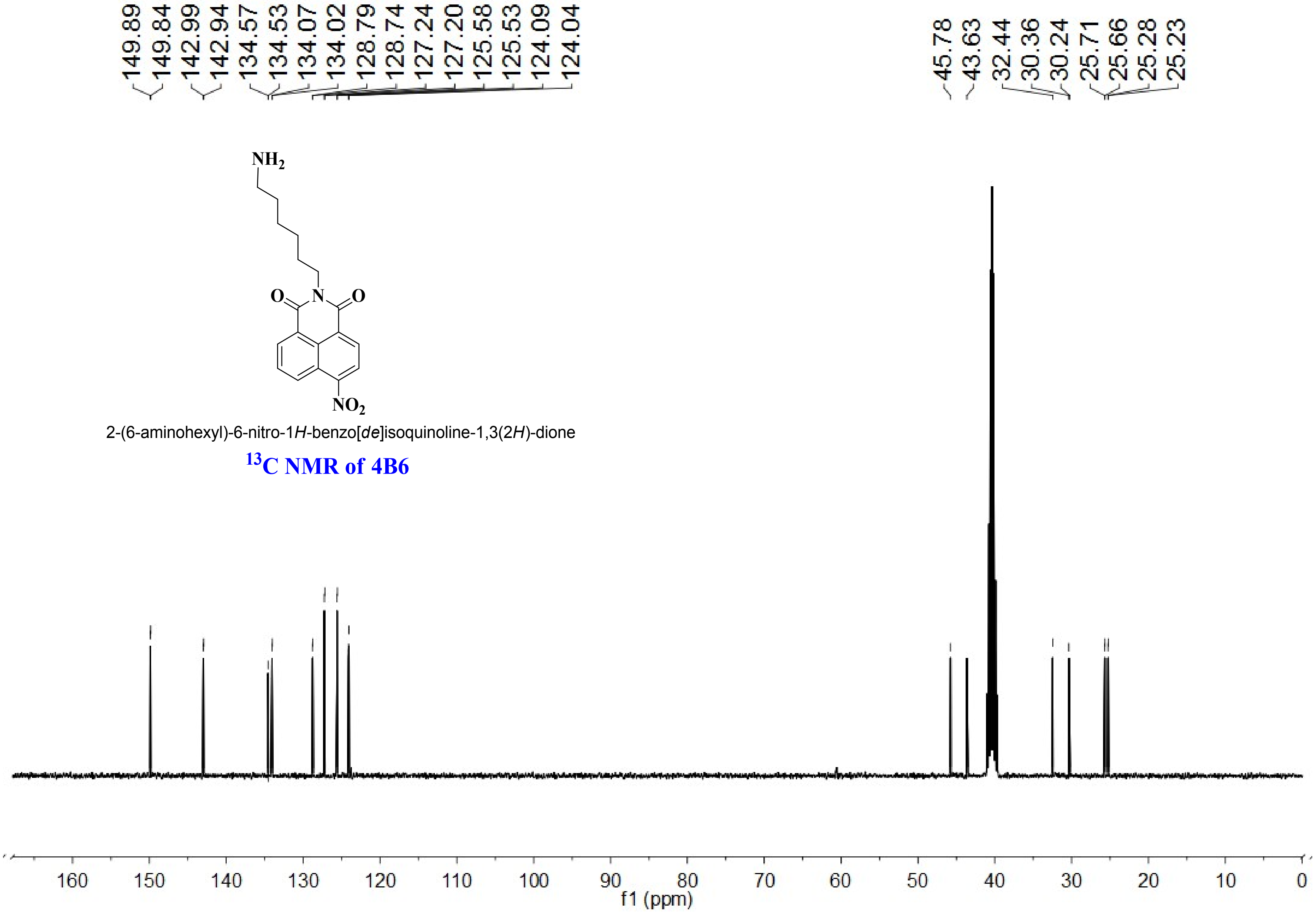

2-(6-aminohexyl)-6-nitro-1*H*-benzo[*de*]isoquinoline-1,3(2*H*)-dione

^{13}C NMR of 4B6

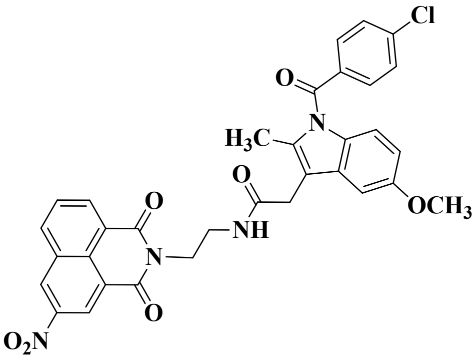

2-(1-(4-chlorobenzoyl)-5-methoxy-2-methyl-1*H*-indol-3-yl)-*N*-(2-(5-nitro-1,3-dioxo-1*H*-benzo[*de*]isoquinolin-2(3*H*)-yl)ethyl)acetamide

^{1}H NMR of 3IA2

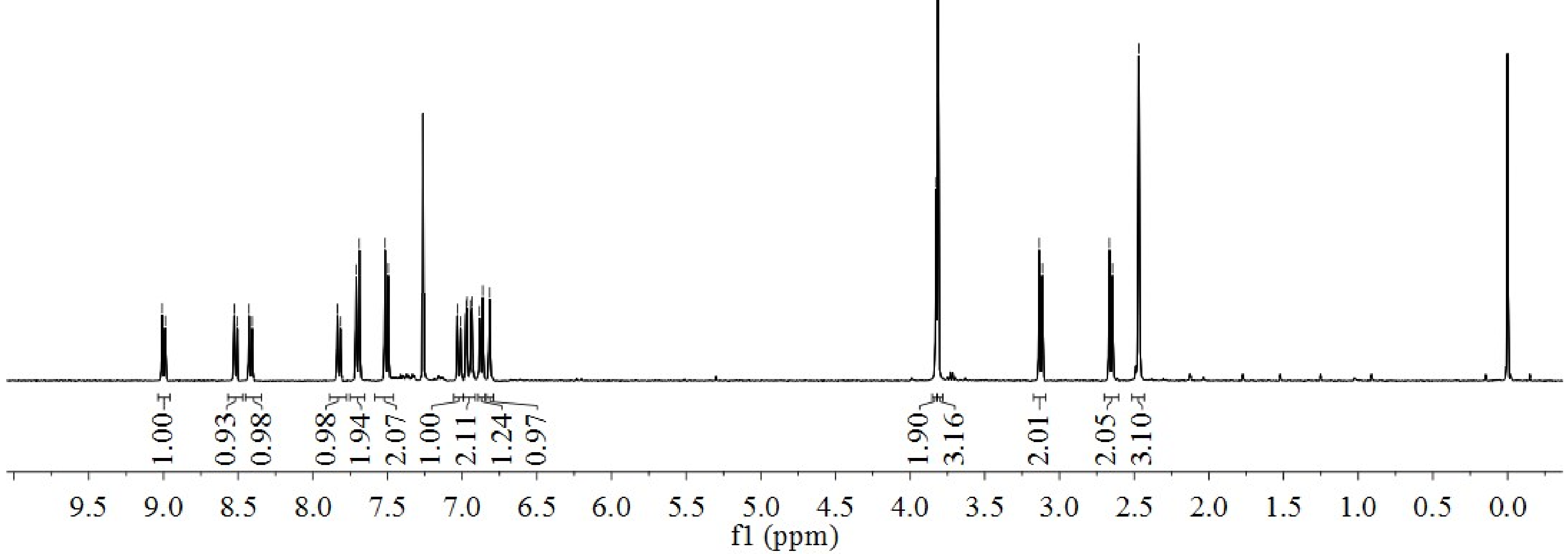

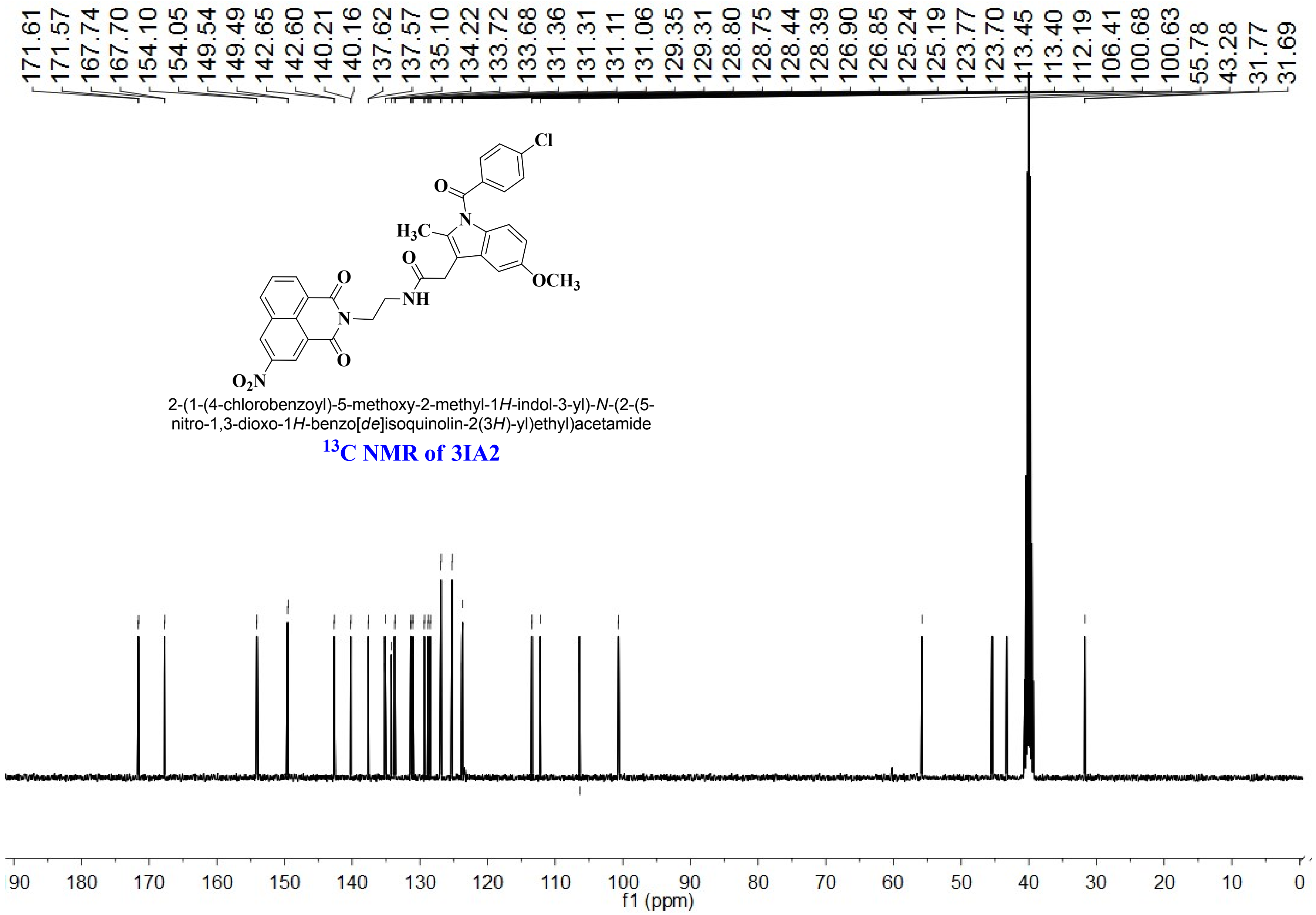

2-(1-(4-chlorobenzoyl)-5-methoxy-2-methyl-1*H*-indol-3-yl)-*N*-(2-(5-nitro-1,3-dioxo-1*H*-benzo[*de*]isoquinolin-2(3*H*)-yl)ethyl)acetamide

^{13}C NMR of 3IA2

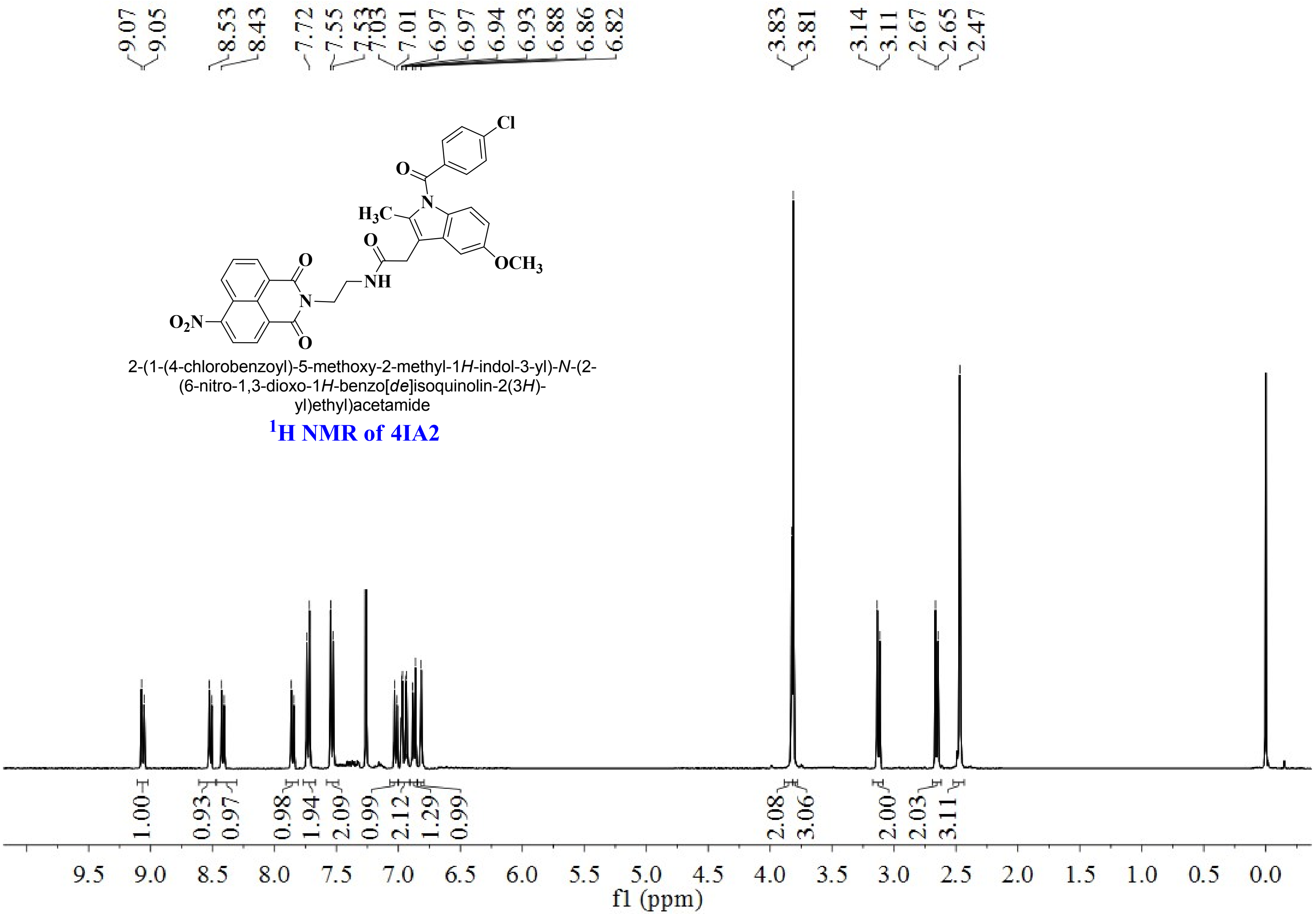

2-(1-(4-chlorobenzoyl)-5-methoxy-2-methyl-1*H*-indol-3-yl)-*N*-(2-(6-nitro-1,3-dioxo-1*H*-benzo[*de*]isoquinolin-2(3*H*)-yl)ethyl)acetamide

^{1}H NMR of 4IA2

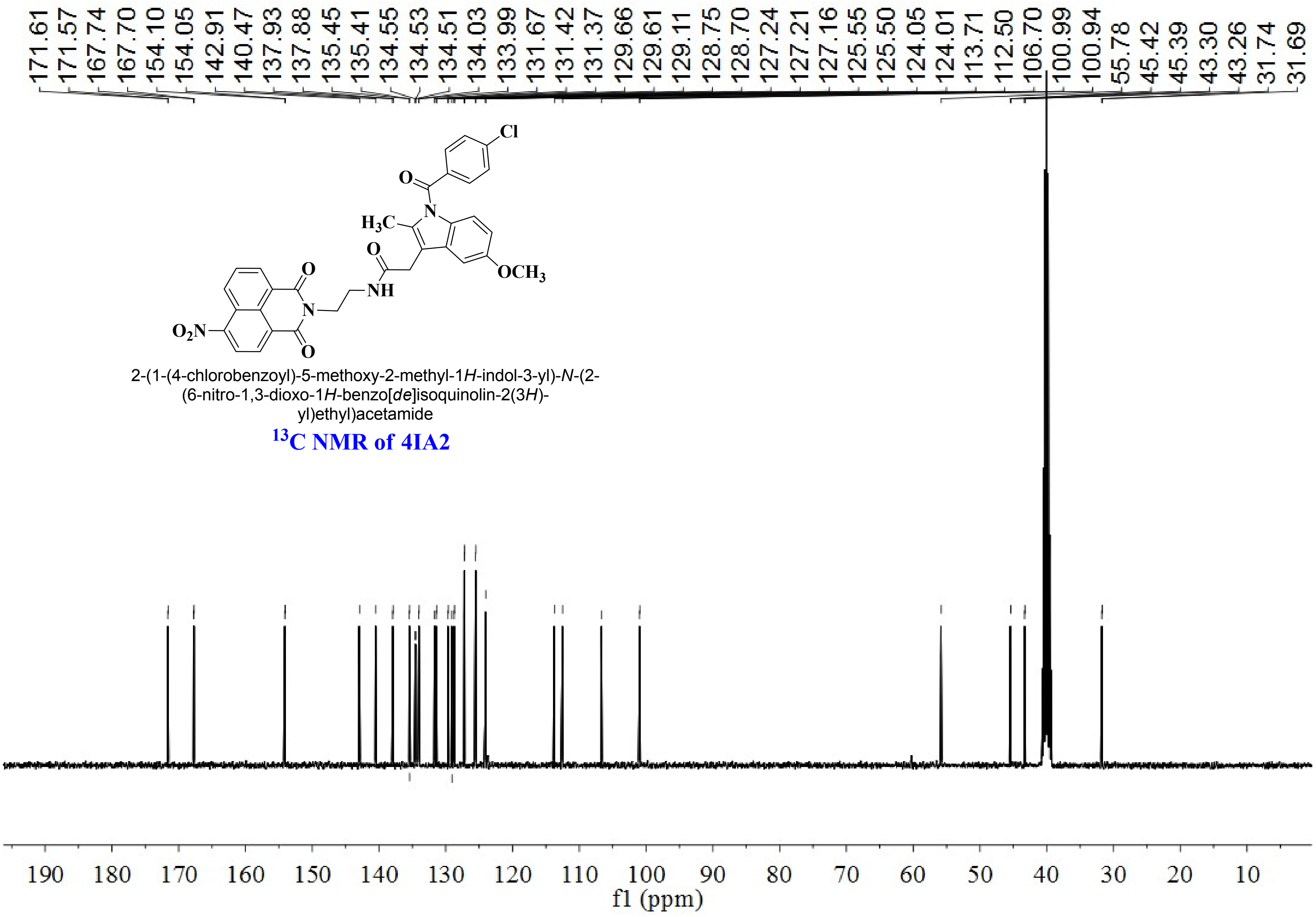
171.61
171.57
167.74
167.70
154.10
154.05
142.91
140.47
137.93
137.88
135.45
135.41
134.55
134.53
134.51
134.03
133.99
131.67
131.42
131.37
129.66
129.61
129.11
128.75
128.70
127.24
127.21
127.16
125.55
125.50
124.05
124.01
113.71
112.50
106.70
100.99
100.94
55.78
45.42
45.39
43.30
43.26
31.74
31.69
Cl
O
N
H_3C
O
O
N
NH
OCH_3
O_2N
O
2-(1-(4-chlorobenzoyl)-5-methoxy-2-methyl-1*H*-indol-3-yl)-*N*-(2-(6-nitro-1,3-dioxo-1*H*-benzo[*de*]isoquinolin-2(3*H*)-yl)ethyl)acetamide
^{13}C NMR of 4IA2
190 180 170 160 150 140 130 120 110 100 90 80 70 60 50 40 30 20 10
f1 (ppm)

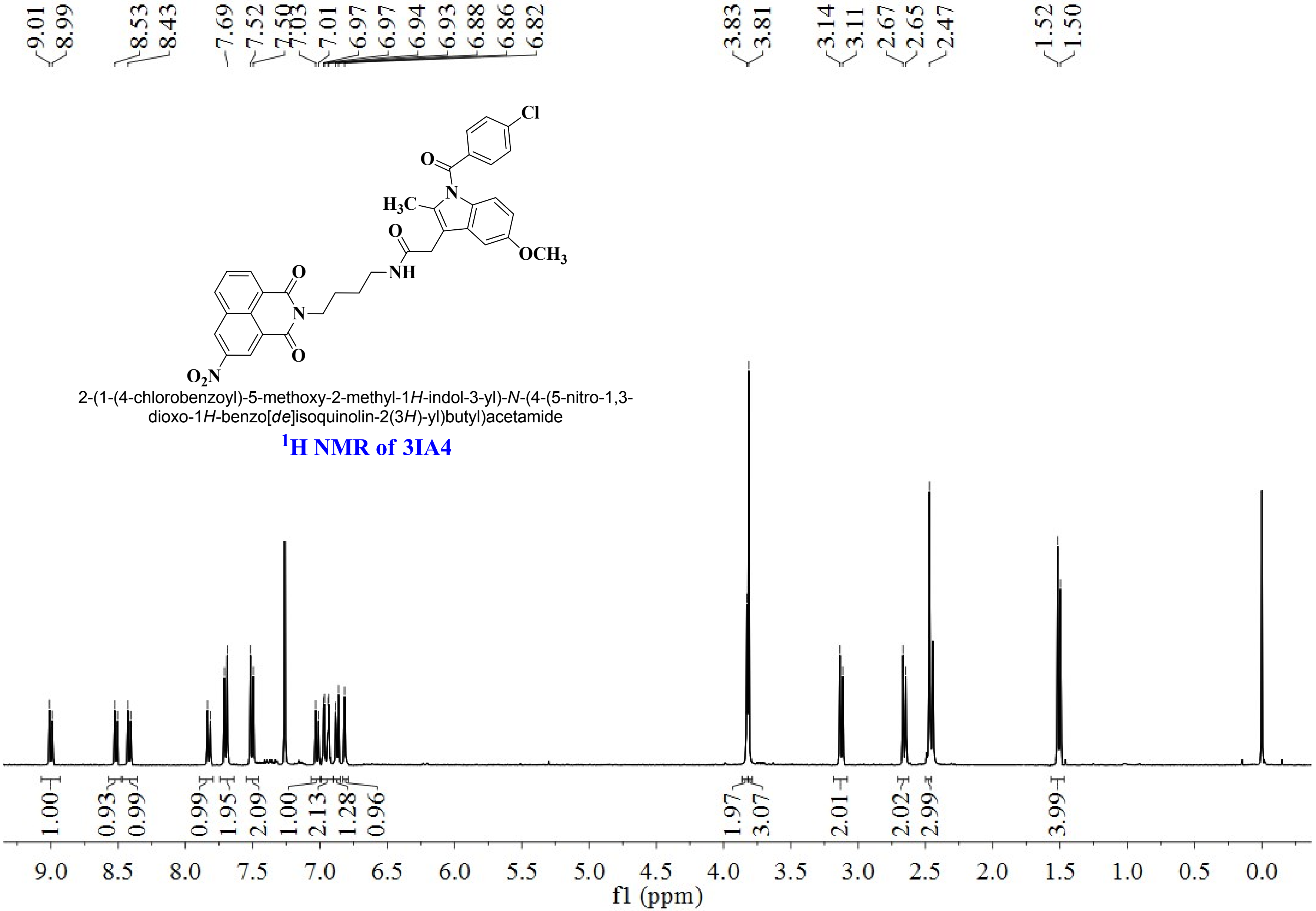

2-(1-(4-chlorobenzoyl)-5-methoxy-2-methyl-1*H*-indol-3-yl)-*N*-(4-(5-nitro-1,3-dioxo-1*H*-benzo[*de*]isoquinolin-2(3*H*)-yl)butyl)acetamide

^{1}H NMR of 3IA4

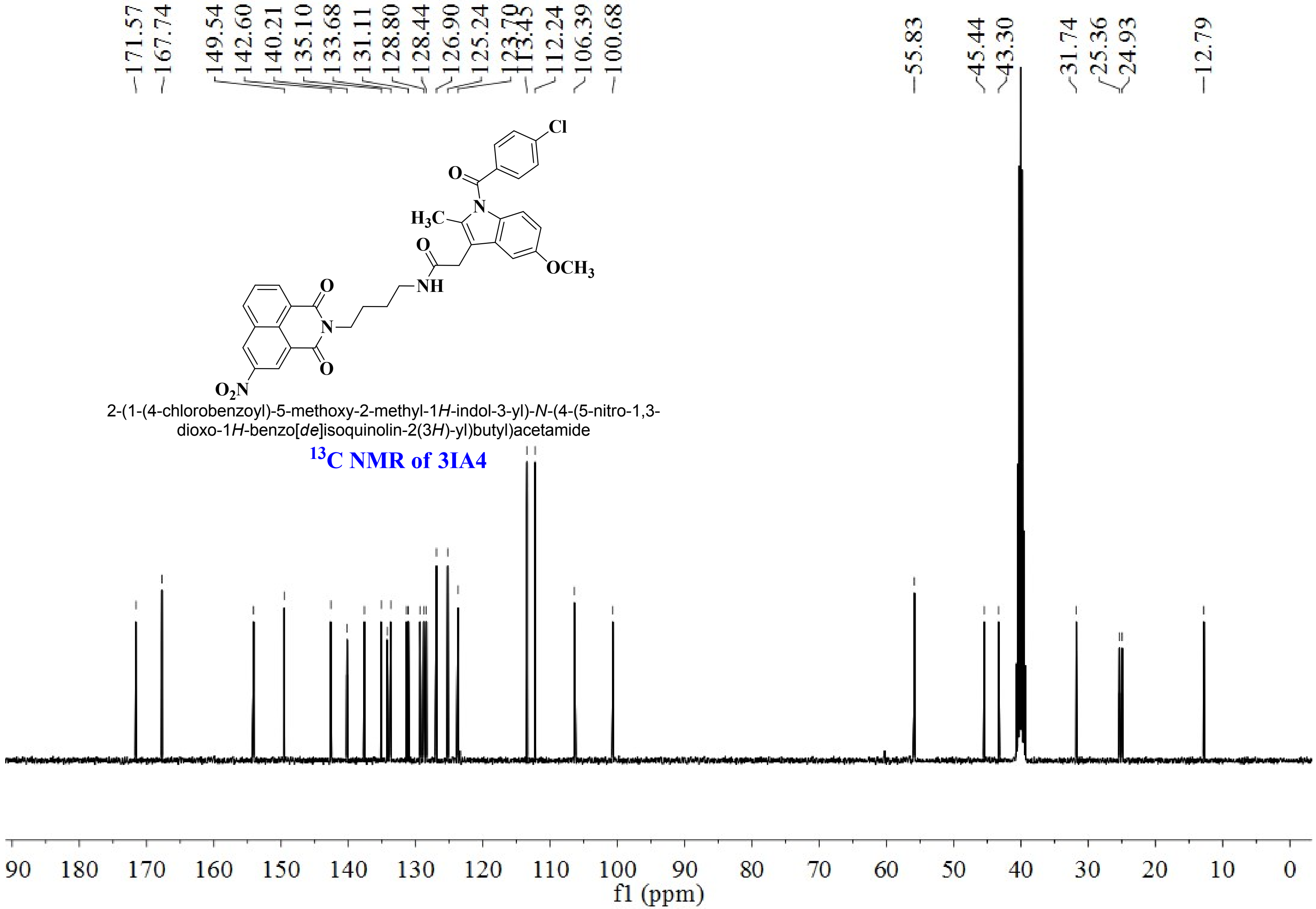
171.57
167.74
149.54
142.60
140.21
135.10
133.68
131.11
128.80
128.44
126.90
125.24
123.70
113.45
112.24
106.39
100.68
55.83
45.44
43.30
31.74
25.36
24.93
12.79
Cl
O
N
H_3C
O
OCH_3
NH
O
N
O
O_2N
2-(1-(4-chlorobenzoyl)-5-methoxy-2-methyl-1*H*-indol-3-yl)-*N*-(4-(5-nitro-1,3-dioxo-1*H*-benzo[*de*]isoquinolin-2(3*H*)-yl)butyl)acetamide
^{13}C NMR of 3IA4
90 180 170 160 150 140 130 120 110 100 90 80 70 60 50 40 30 20 10 0
f1 (ppm)

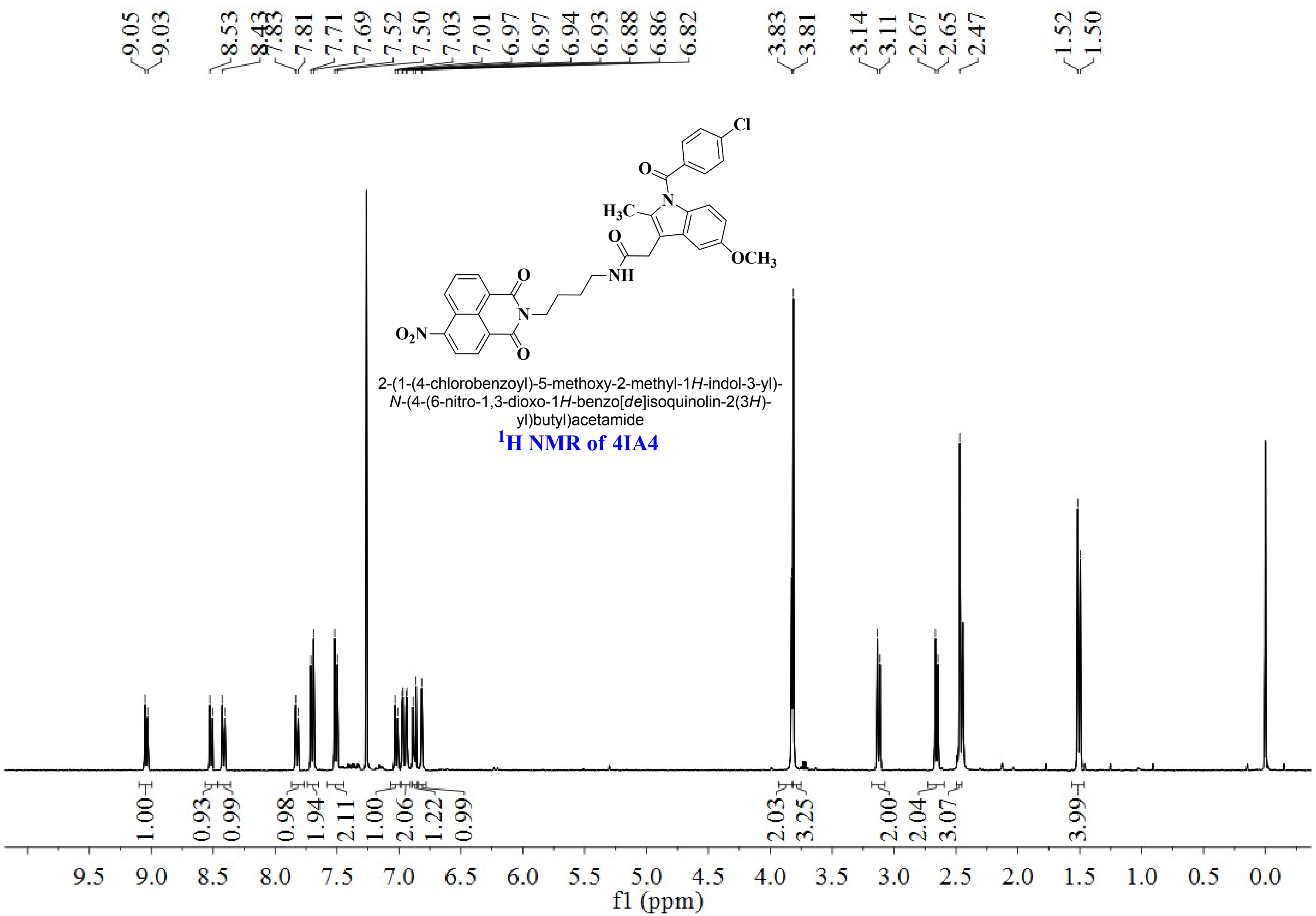

2-(1-(4-chlorobenzoyl)-5-methoxy-2-methyl-1*H*-indol-3-yl)-*N*-(4-(6-nitro-1,3-dioxo-1*H*-benzo[*de*]isoquinolin-2(3*H*)-yl)butyl)acetamide

^{1}H NMR of 4IA4

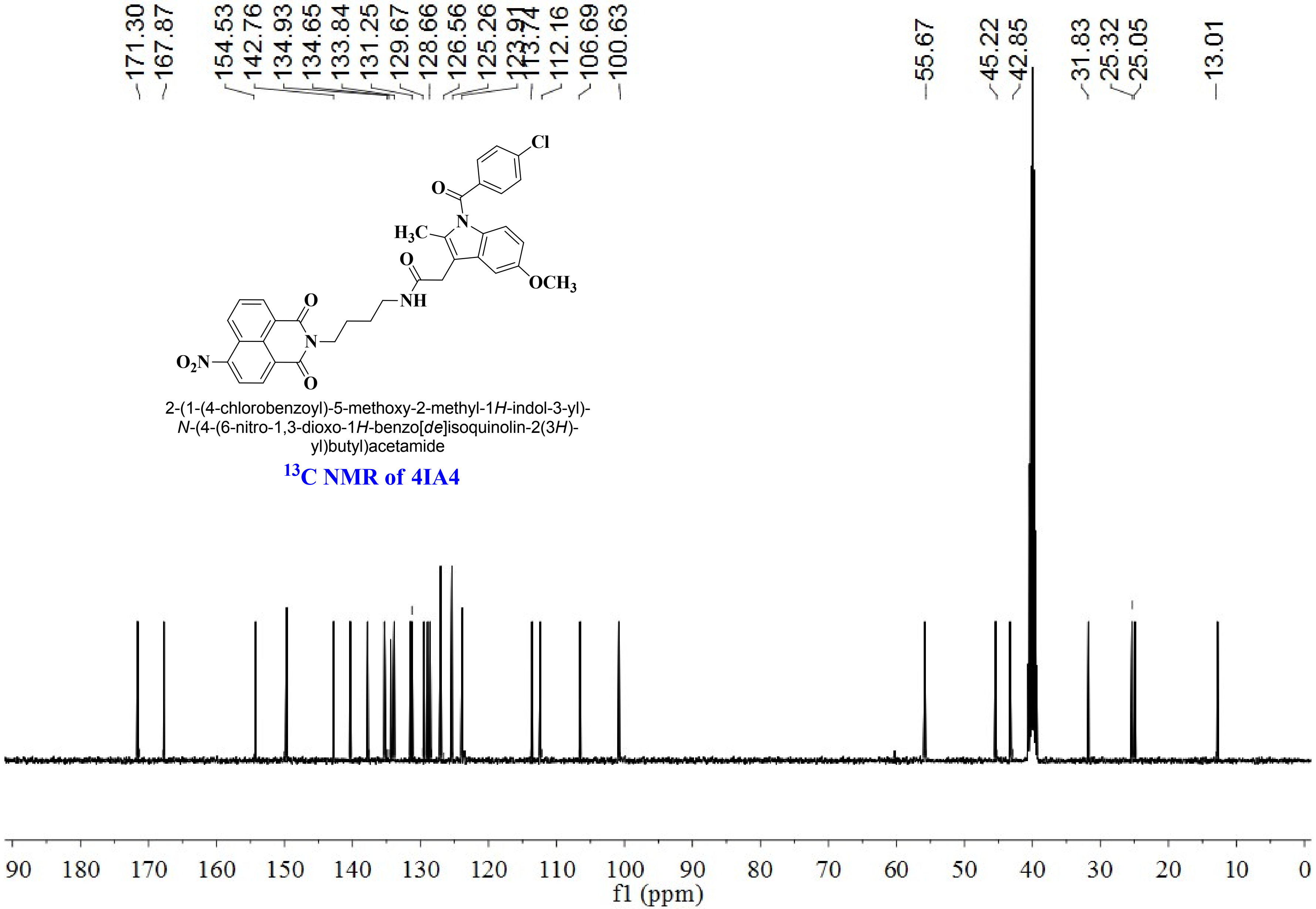
171.30
167.87
154.53
142.76
134.93
134.65
133.84
131.25
129.67
128.66
126.56
125.26
123.91
113.74
112.16
106.69
100.63
55.67
45.22
42.85
31.83
25.32
25.05
13.01
Cl
O
N
H_3C
O
OCH_3
NH
O
N
O_2N
O
2-(1-(4-chlorobenzoyl)-5-methoxy-2-methyl-1*H*-indol-3-yl)-
N-(4-(6-nitro-1,3-dioxo-1*H*-benzo[*de*]isoquinolin-2(3*H*)-
yl)butyl)acetamide
^{13}C NMR of 4IA4
90 180 170 160 150 140 130 120 110 100 90 80 70 60 50 40 30 20 10 0
f1 (ppm)

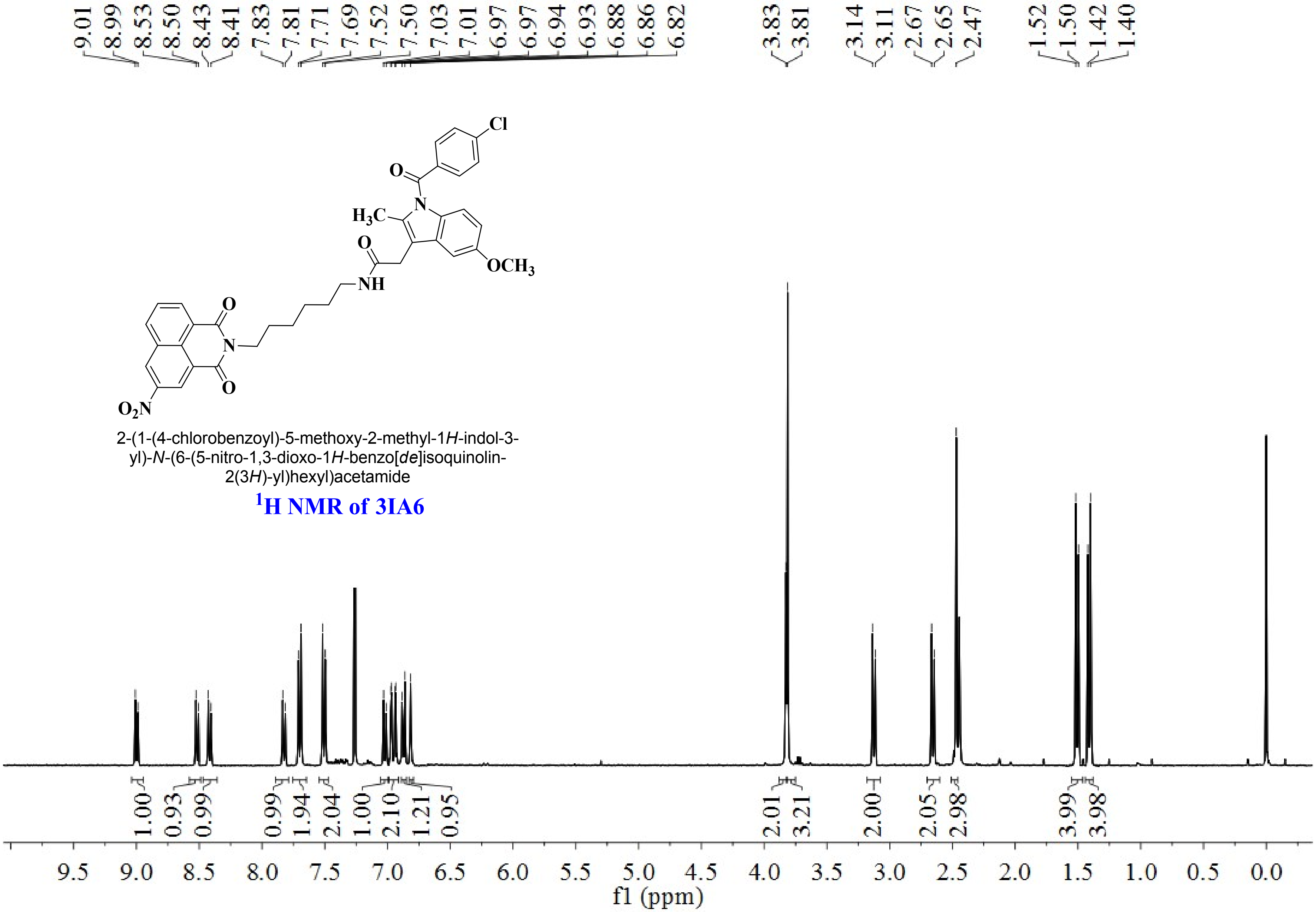

2-(1-(4-chlorobenzoyl)-5-methoxy-2-methyl-1*H*-indol-3-yl)-*N*-(6-(5-nitro-1,3-dioxo-1*H*-benzo[*de*]isoquinolin-2(3*H*)-yl)hexyl)acetamide

^{1}H NMR of 3IA6

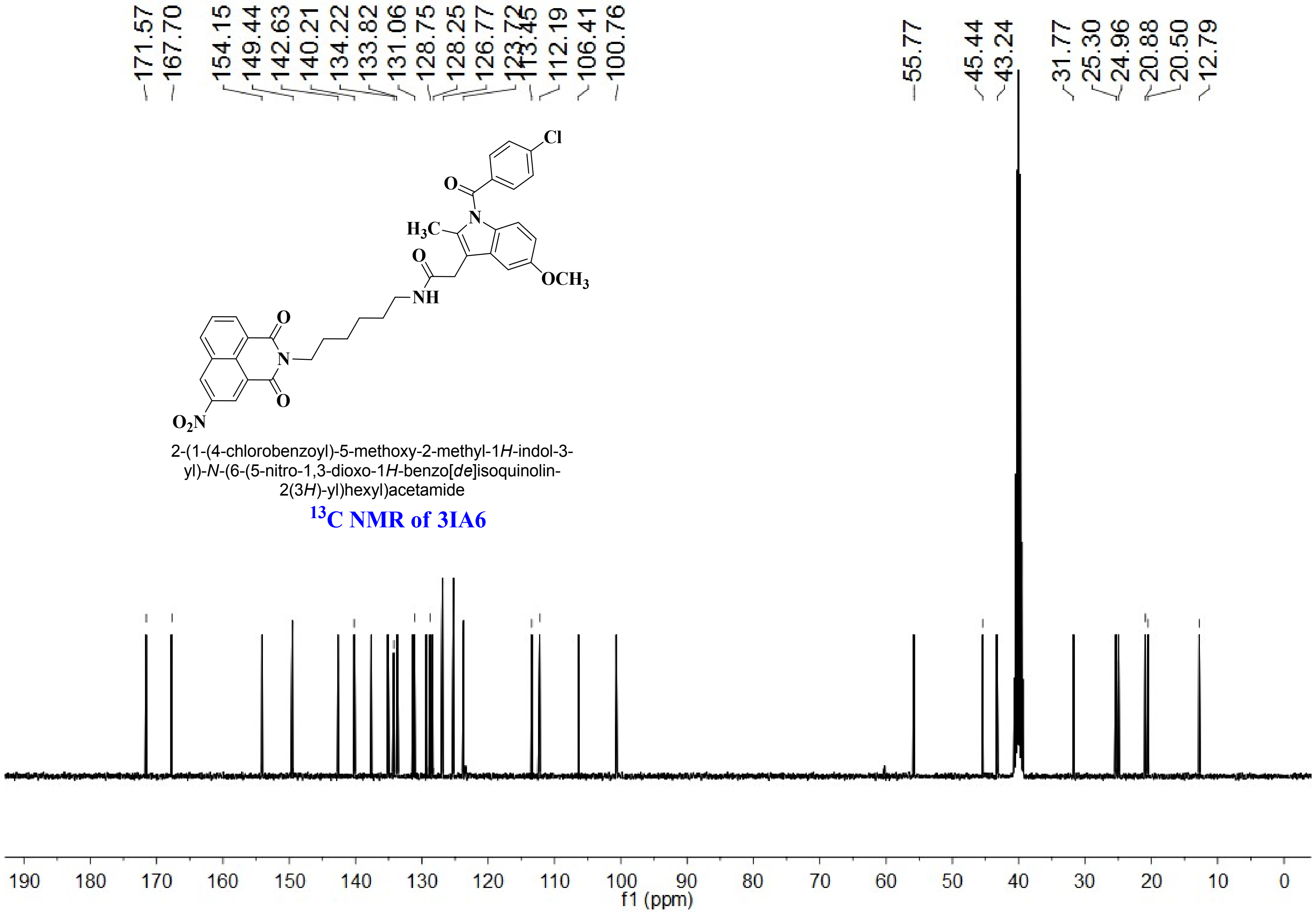
171.57
167.70
154.15
149.44
142.63
140.21
134.22
133.82
131.06
128.75
128.25
126.77
123.72
113.45
112.19
106.41
100.76
55.77
45.44
43.24
31.77
25.30
24.96
20.88
20.50
12.79
Cl
O
N
H_3C
O
OCH_3
NH
O
N
O
O_2N
2-(1-(4-chlorobenzoyl)-5-methoxy-2-methyl-1*H*-indol-3-yl)-*N*-(6-(5-nitro-1,3-dioxo-1*H*-benzo[*de*]isoquinolin-2(3*H*)-yl)hexyl)acetamide
^{13}C NMR of 3IA6
190 180 170 160 150 140 130 120 110 100 90 80 70 60 50 40 30 20 10 0
f1 (ppm)

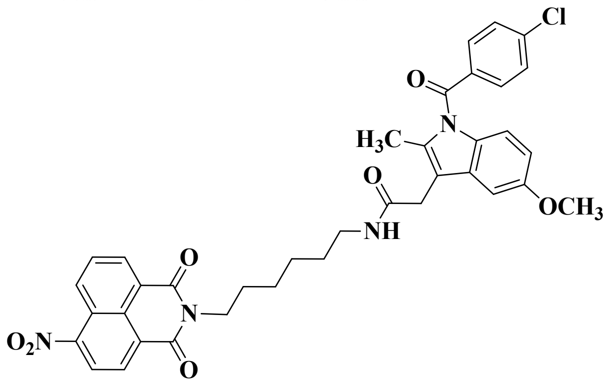

2-(1-(4-chlorobenzoyl)-5-methoxy-2-methyl-1*H*-indol-3-yl)-*N*-(6-(6-nitro-1,3-dioxo-1*H*-benzo[*de*]isoquinolin-2(3*H*)-yl)hexyl)acetamide

^{1}H NMR of 4IA6

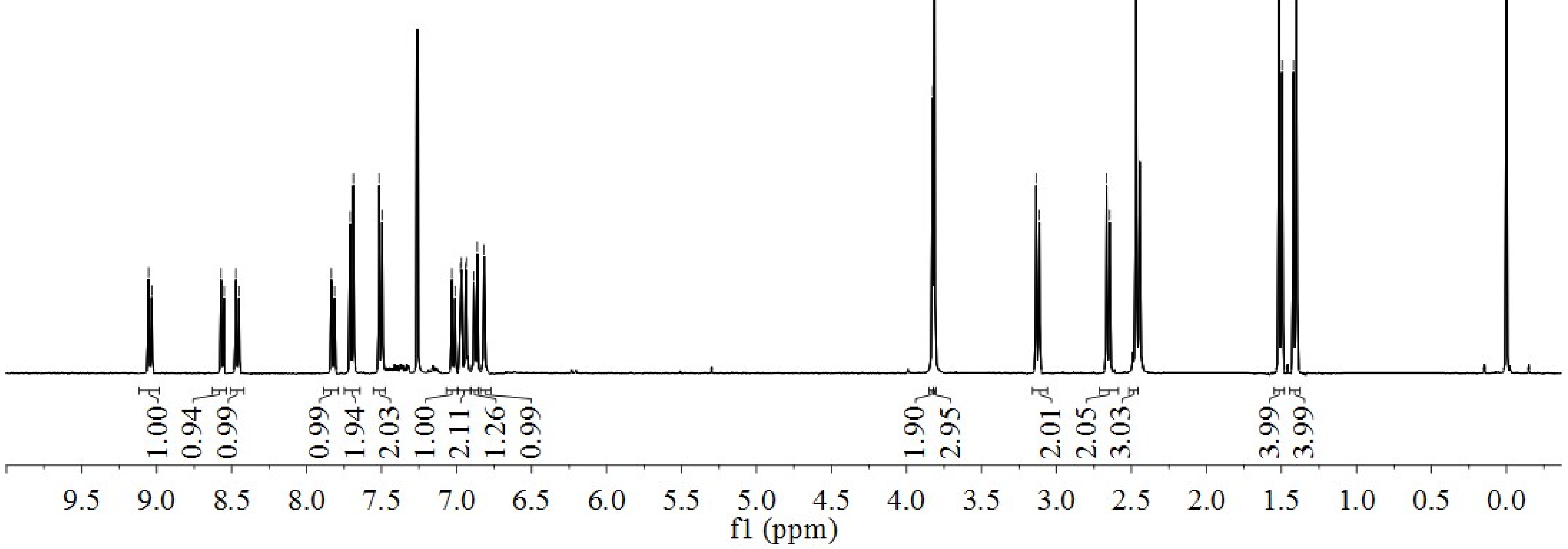

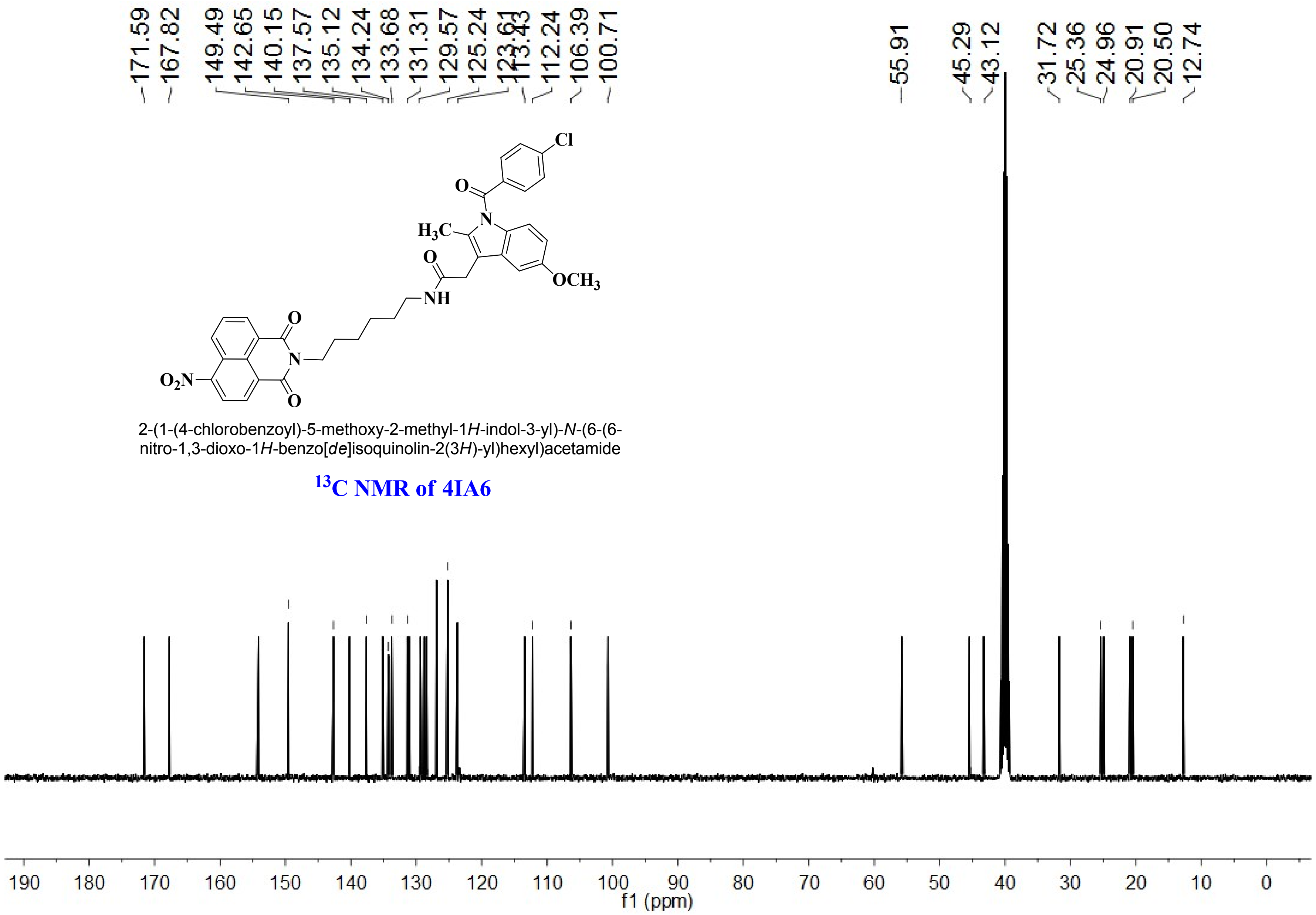

2-(1-(4-chlorobenzoyl)-5-methoxy-2-methyl-1*H*-indol-3-yl)-*N*-(6-(6-nitro-1,3-dioxo-1*H*-benzo[*de*]isoquinolin-2(3*H*)-yl)hexyl)acetamide

^{13}C NMR of 4IA6

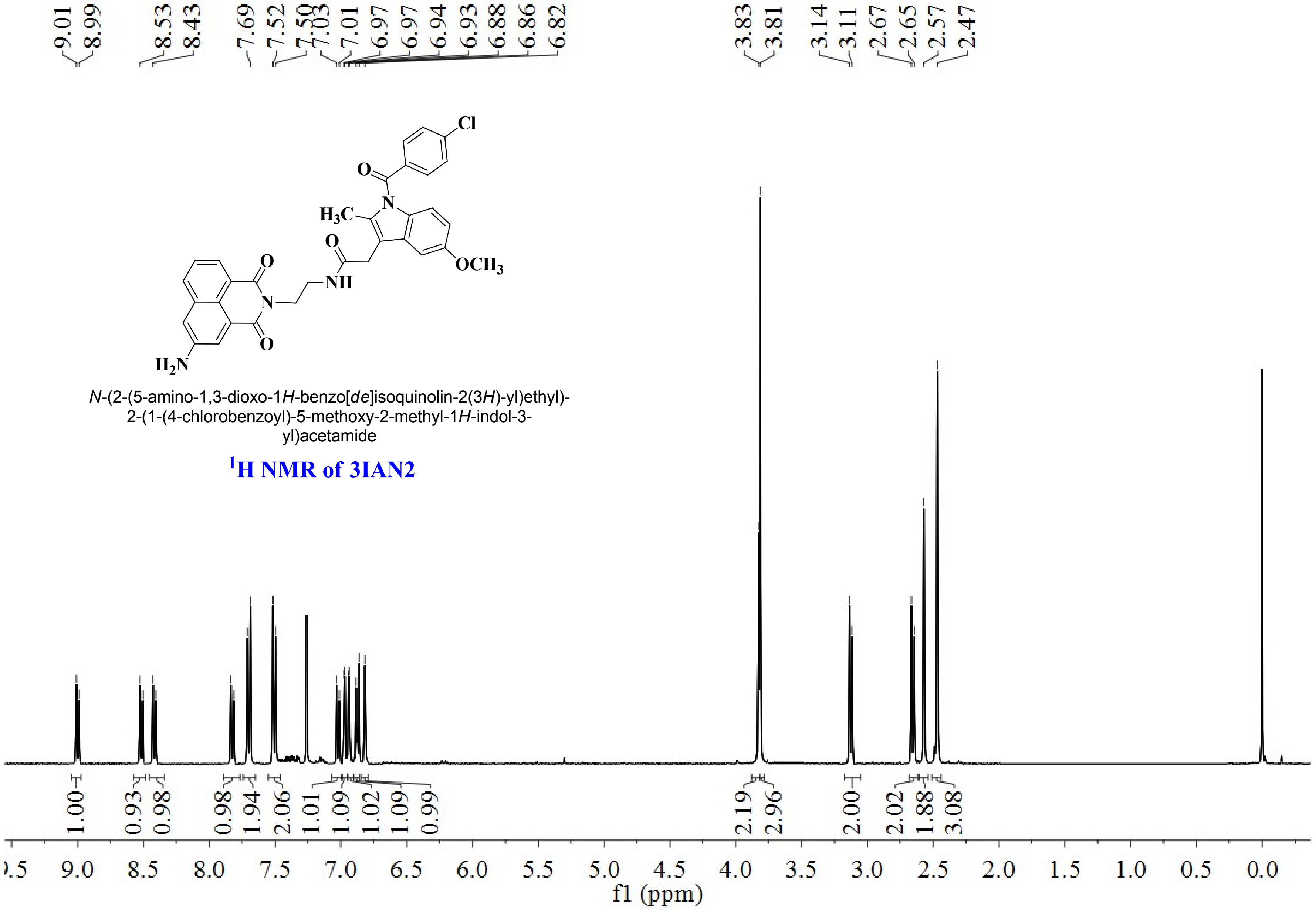

N-(2-(5-amino-1,3-dioxo-1*H*-benzo[*de*]isoquinolin-2(3*H*)-yl)ethyl)-2-(1-(4-chlorobenzoyl)-5-methoxy-2-methyl-1*H*-indol-3-yl)acetamide

^{1}H NMR of 3IAN2

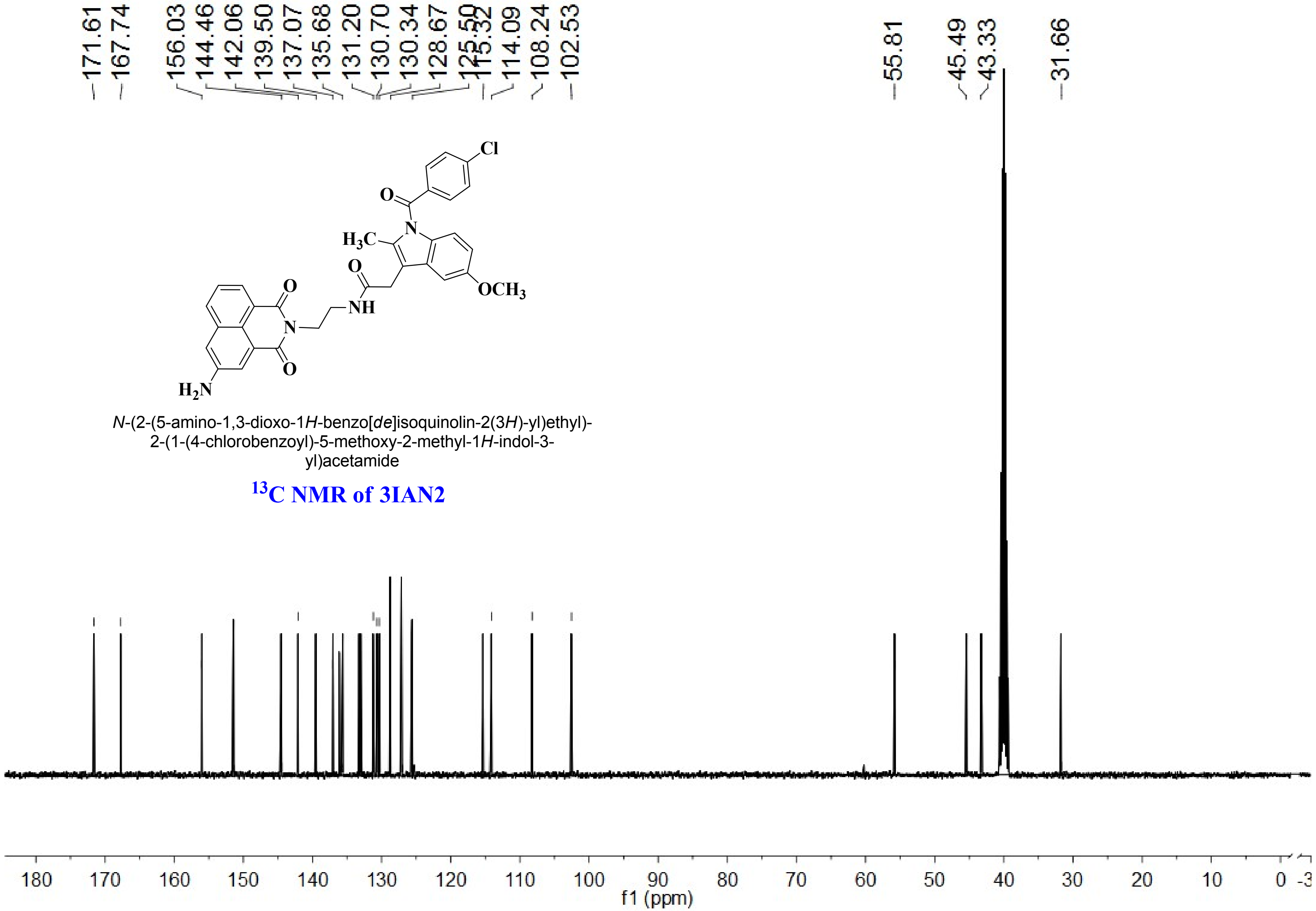

N-(2-(5-amino-1,3-dioxo-1*H*-benzo[*de*]isoquinolin-2(3*H*)-yl)ethyl)-2-(1-(4-chlorobenzoyl)-5-methoxy-2-methyl-1*H*-indol-3-yl)acetamide

^{13}C NMR of 3IAN2

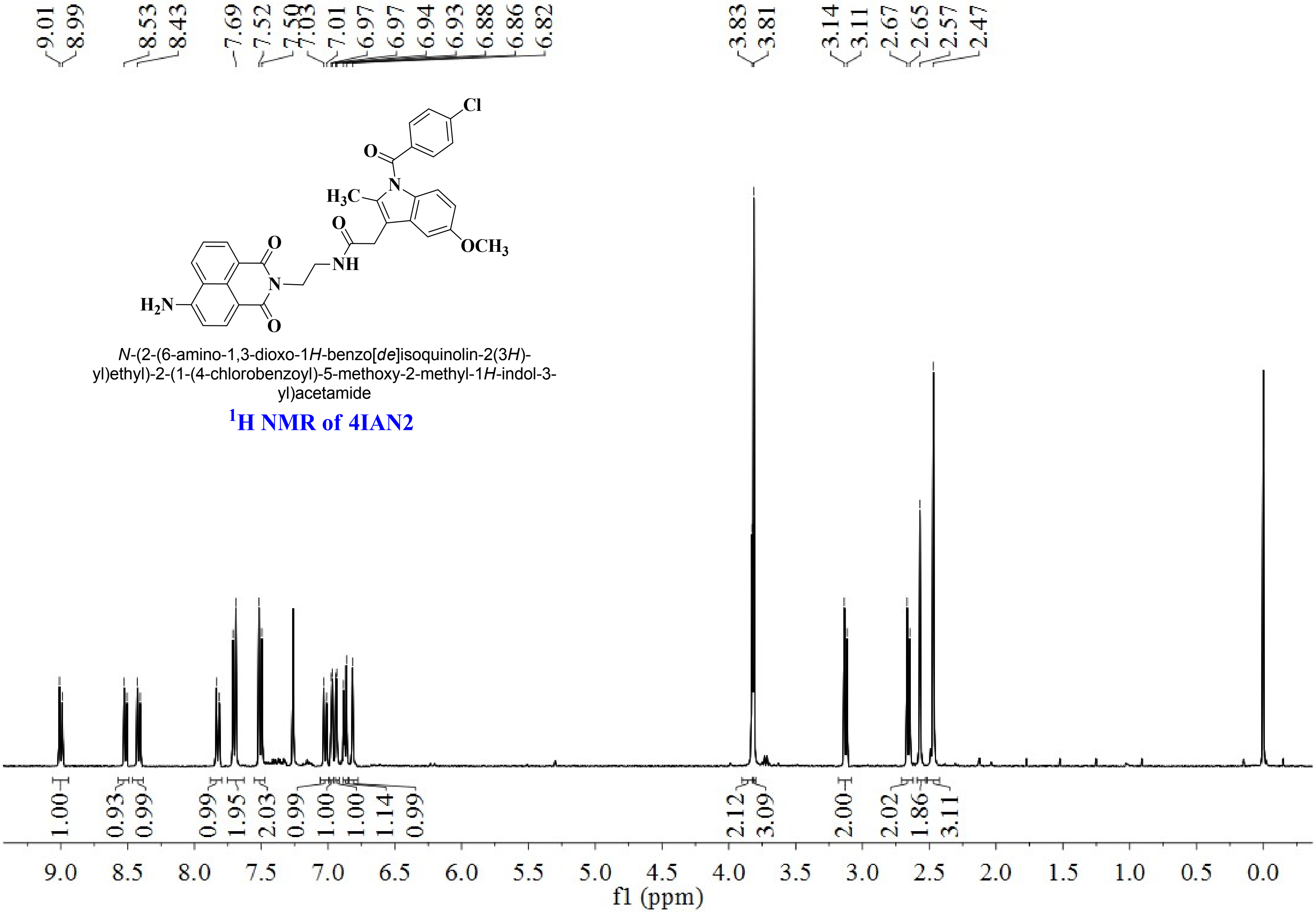

N-(2-(6-amino-1,3-dioxo-1*H*-benzo[*de*]isoquinolin-2(3*H*)-yl)ethyl)-2-(1-(4-chlorobenzoyl)-5-methoxy-2-methyl-1*H*-indol-3-yl)acetamide

^{1}H NMR of 4IAN2

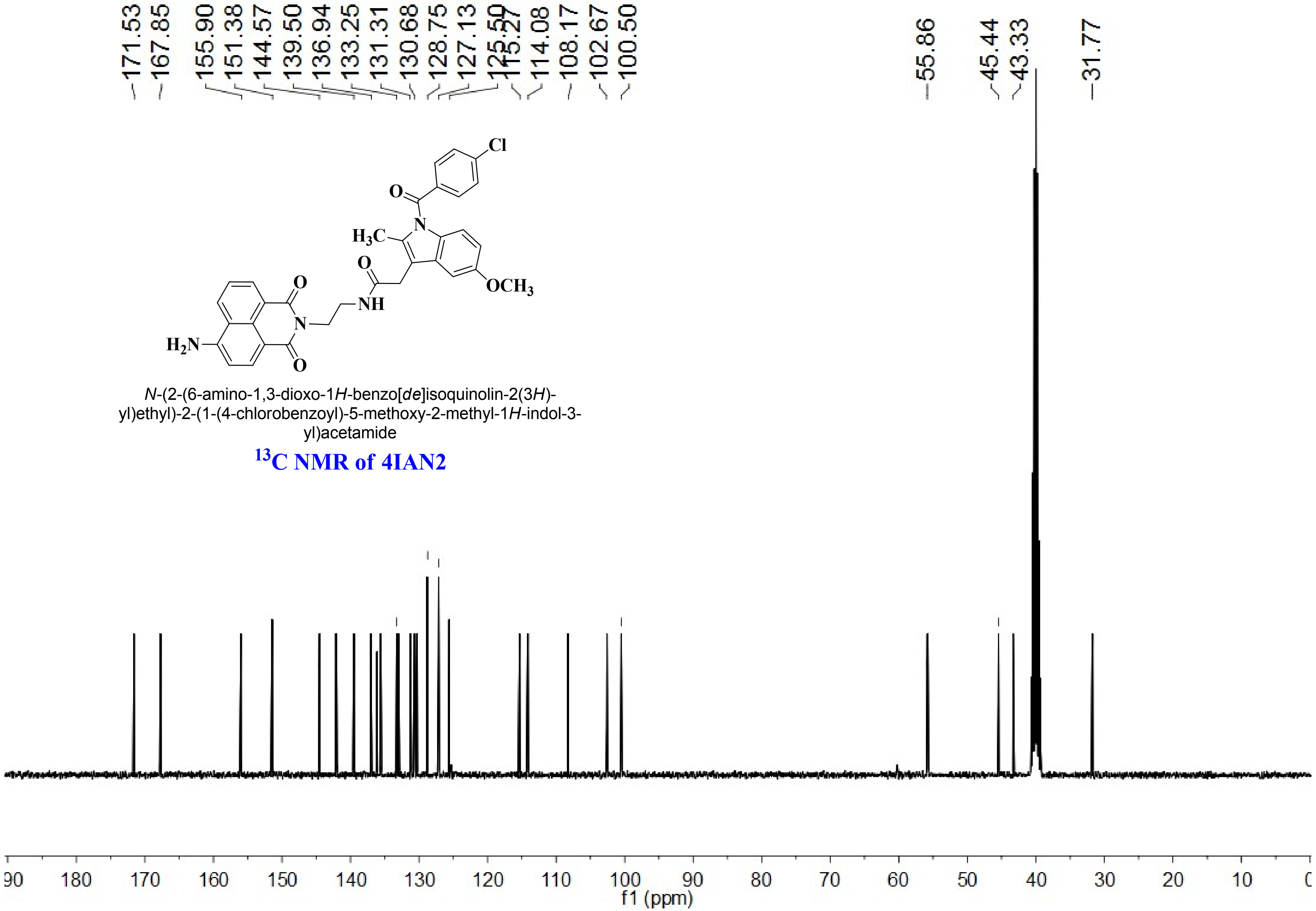

N-(2-(6-amino-1,3-dioxo-1*H*-benzo[*de*]isoquinolin-2(3*H*)-yl)ethyl)-2-(1-(4-chlorobenzoyl)-5-methoxy-2-methyl-1*H*-indol-3-yl)acetamide

^{13}C NMR of 4IAN2

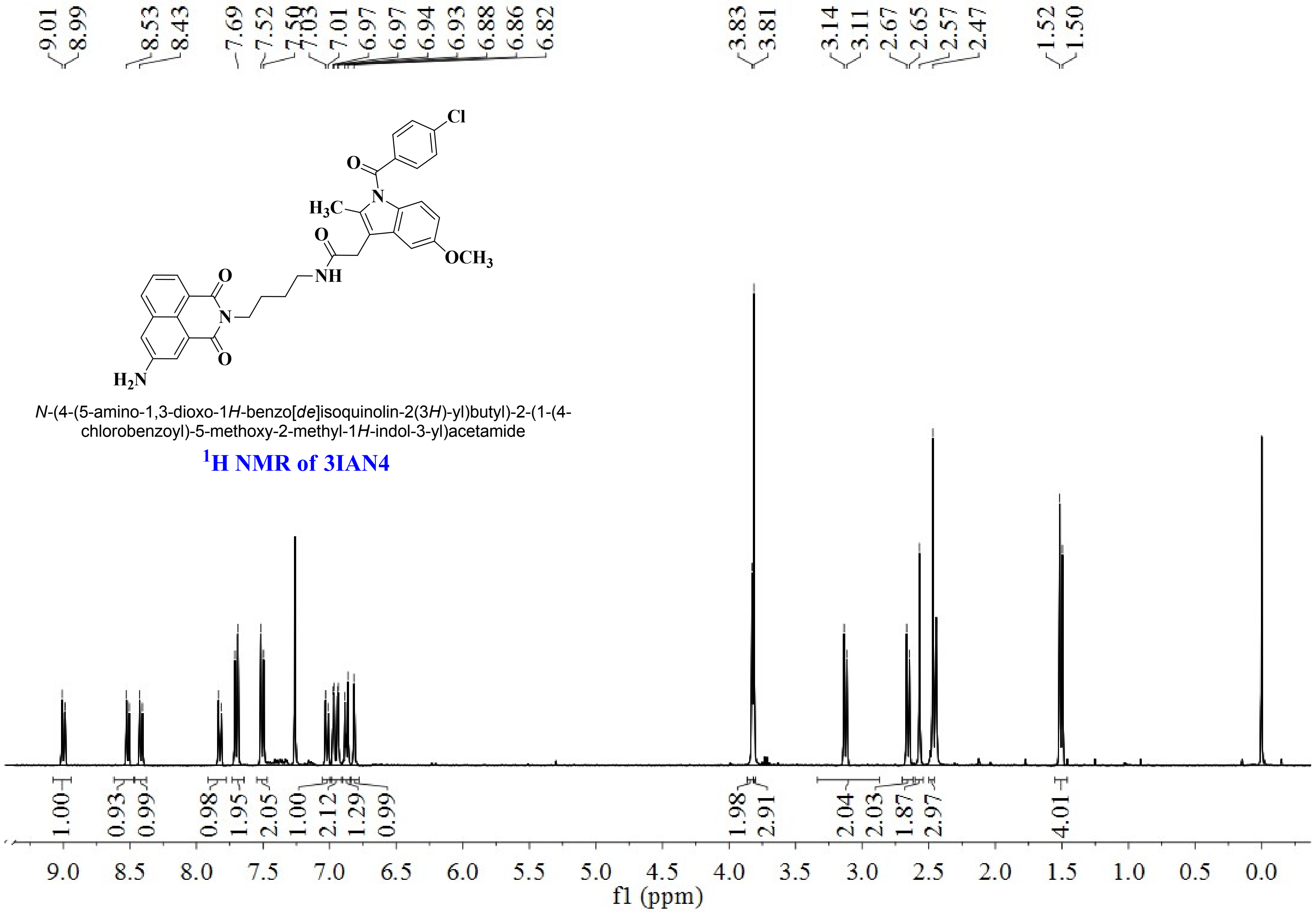

N-(4-(5-amino-1,3-dioxo-1*H*-benzo[*de*]isoquinolin-2(3*H*)-yl)butyl)-2-(1-(4-chlorobenzoyl)-5-methoxy-2-methyl-1*H*-indol-3-yl)acetamide

^{1}H NMR of 3IAN4

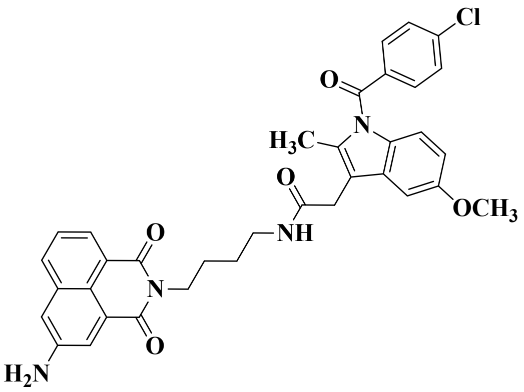

N-(4-(5-amino-1,3-dioxo-1*H*-benzo[*de*]isoquinolin-2(3*H*)-yl)butyl)-2-(1-(4-chlorobenzoyl)-5-methoxy-2-methyl-1*H*-indol-3-yl)acetamide

^{13}C NMR of 3IAN4

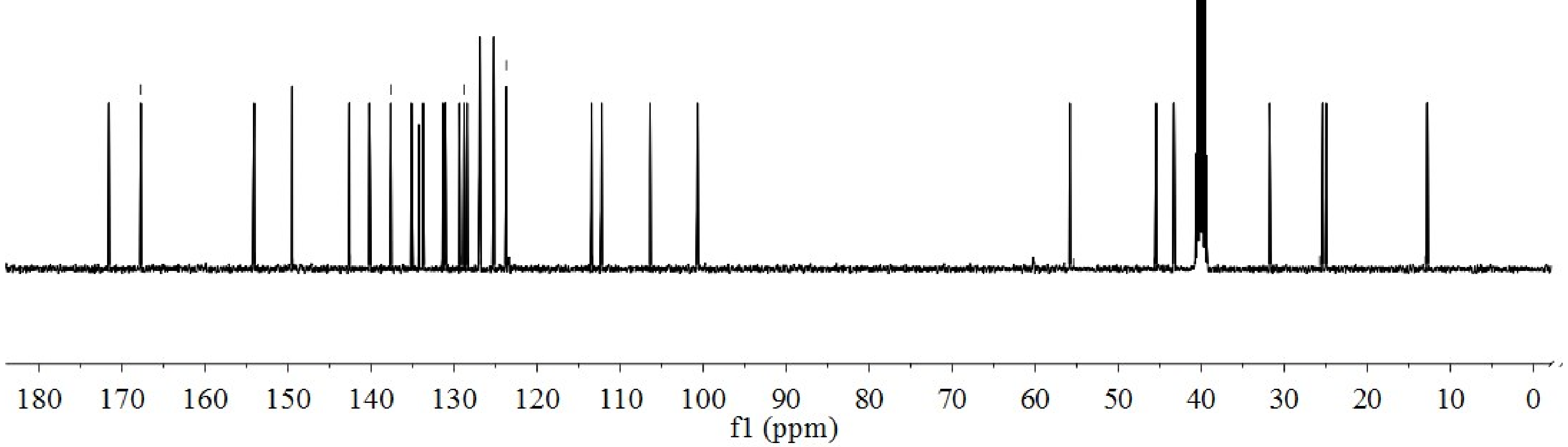

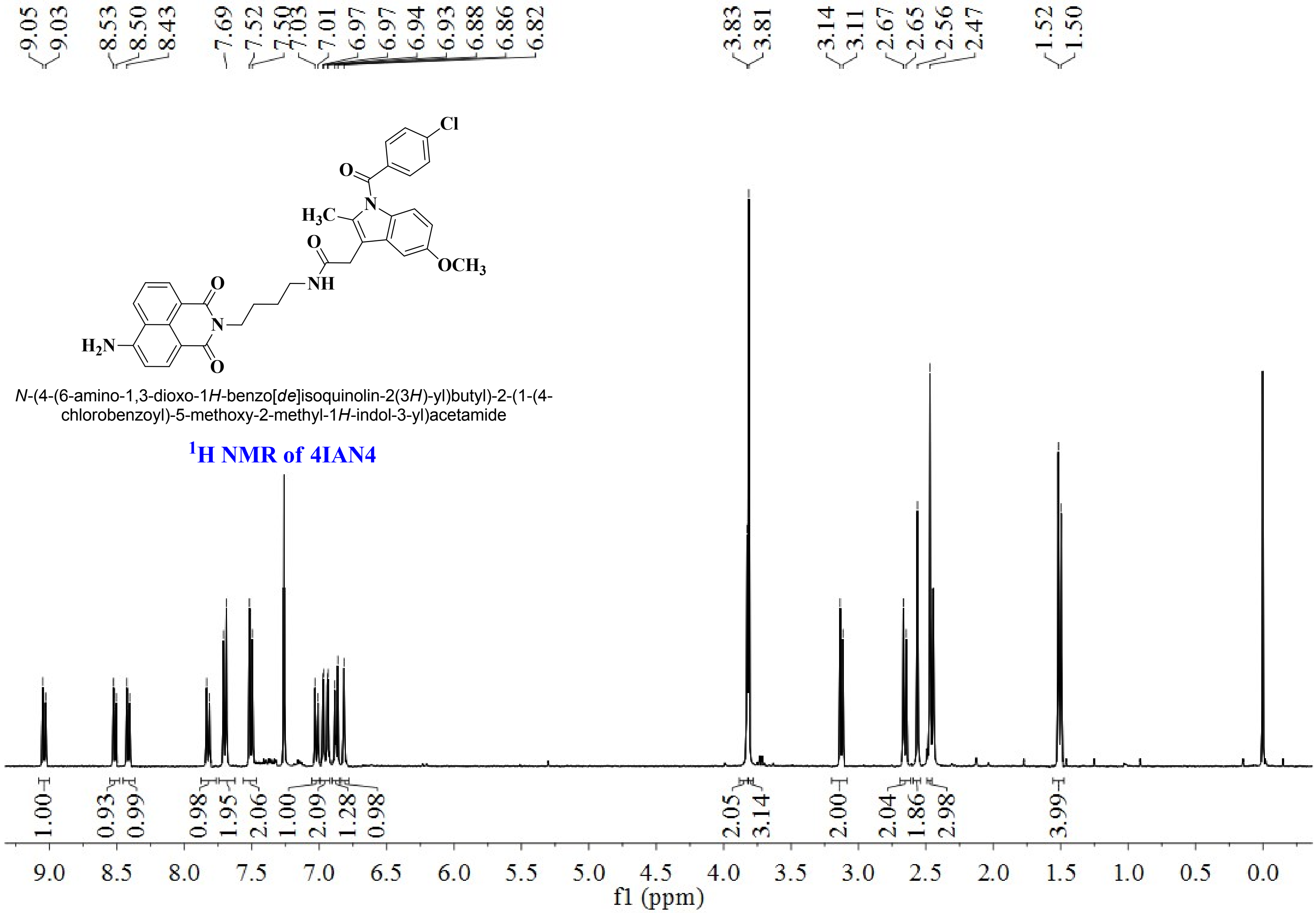

N-(4-(6-amino-1,3-dioxo-1*H*-benzo[*de*]isoquinolin-2(3*H*)-yl)butyl)-2-(1-(4-chlorobenzoyl)-5-methoxy-2-methyl-1*H*-indol-3-yl)acetamide

^{1}H NMR of 4IAN4

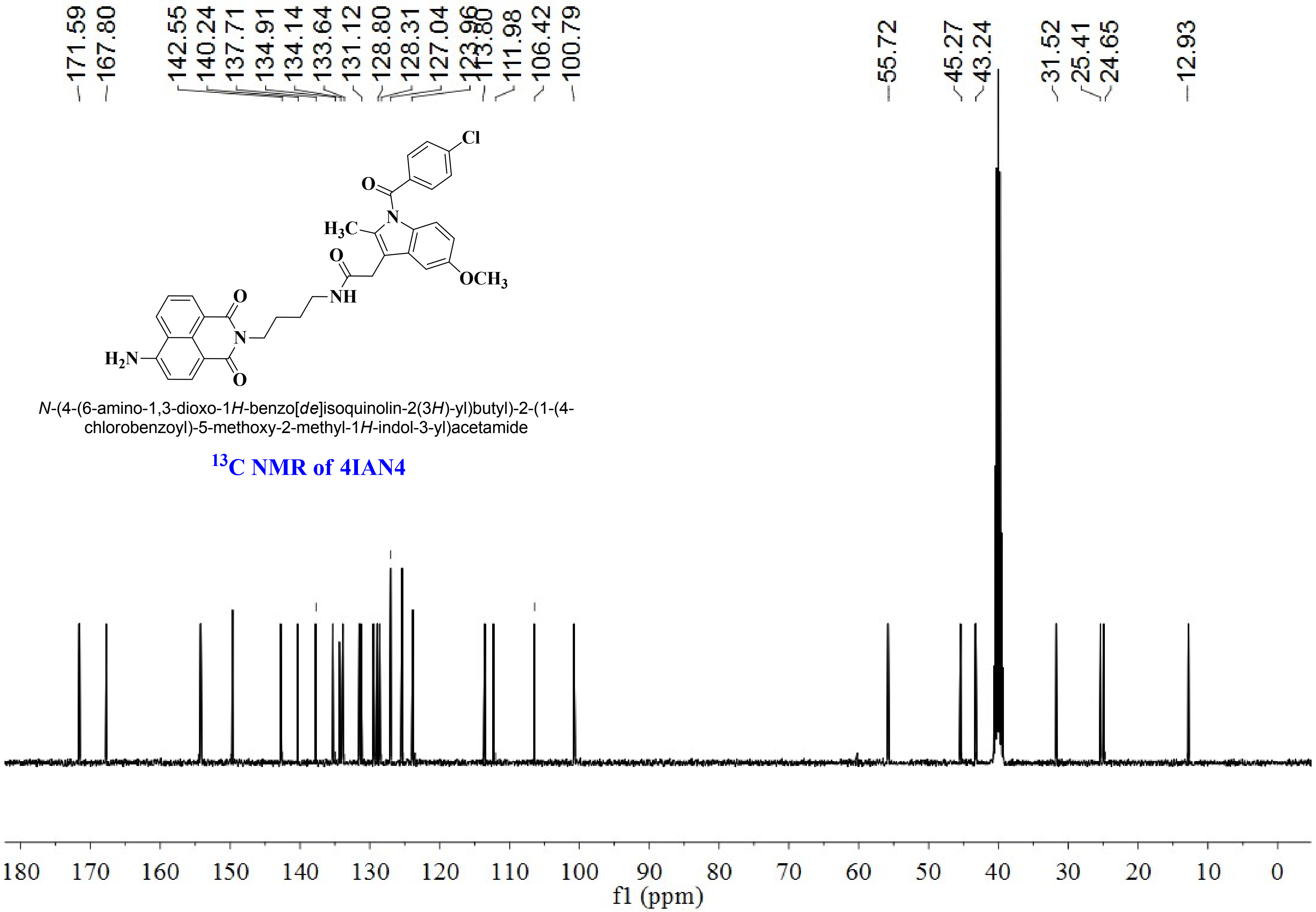

N-(4-(6-amino-1,3-dioxo-1*H*-benzo[*de*]isoquinolin-2(3*H*)-yl)butyl)-2-(1-(4-chlorobenzoyl)-5-methoxy-2-methyl-1*H*-indol-3-yl)acetamide

^{13}C NMR of 4IAN4

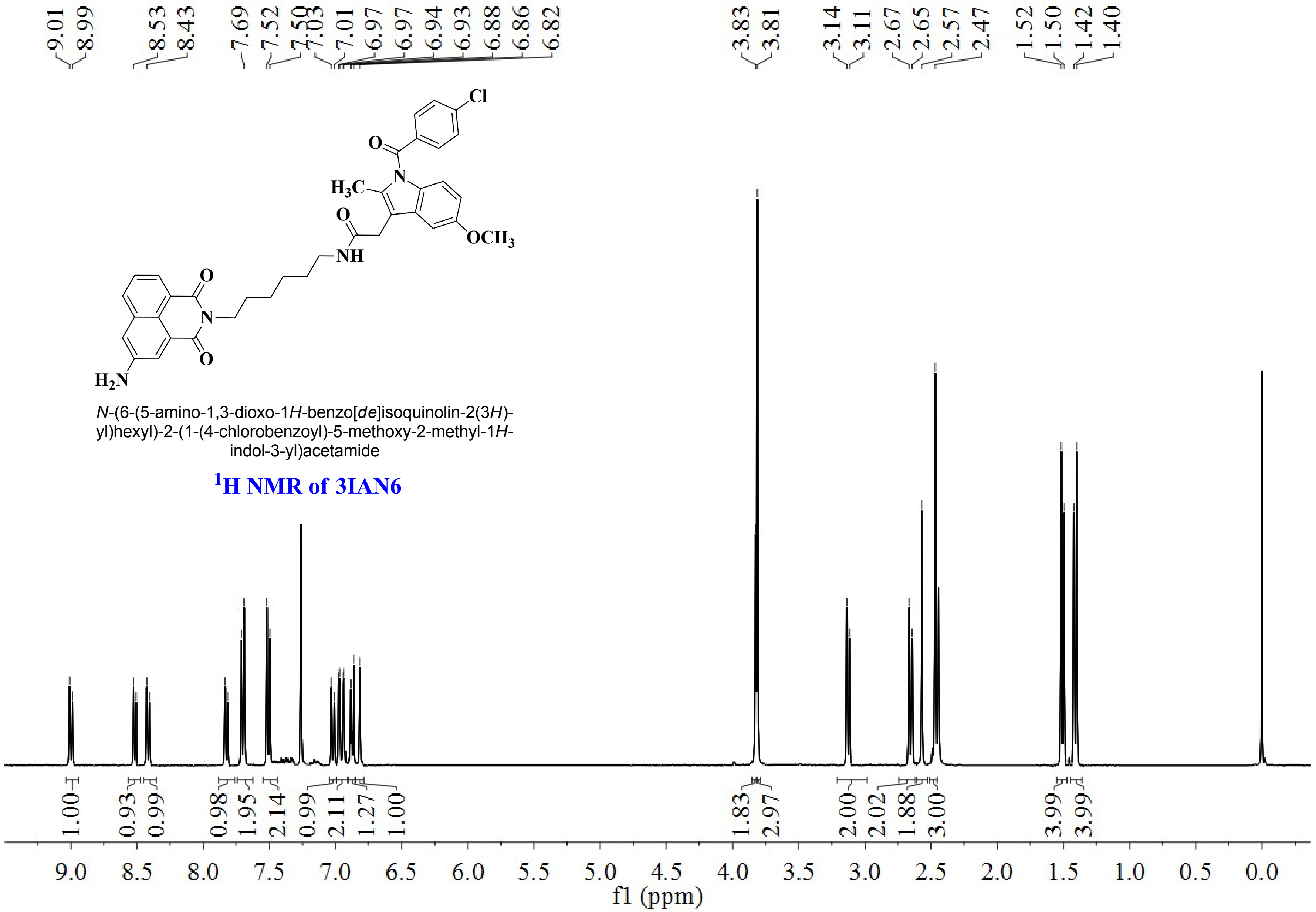
9.01
8.99
8.53
8.43
7.69
7.52
7.50
7.03
7.01
6.97
6.97
6.94
6.93
6.88
6.86
6.82
3.83
3.81
3.14
3.11
2.67
2.65
2.57
2.47
1.52
1.50
1.42
1.40
Cl
O
N
H_3C
O
OCH_3
NH
O
N
O
H_2N
N-(6-(5-amino-1,3-dioxo-1H-benzo[de]isoquinolin-2(3H)-yl)hexyl)-2-(1-(4-chlorobenzoyl)-5-methoxy-2-methyl-1H-indol-3-yl)acetamide
^{1}H NMR of 3IAN6
1.00
0.93
0.99
0.98
1.95
2.14
0.99
2.11
1.27
1.00
1.83
2.97
2.00
2.02
1.88
3.00
3.99
3.99
9.0
8.5
8.0
7.5
7.0
6.5
6.0
5.5
5.0
4.5
4.0
3.5
3.0
2.5
2.0
1.5
1.0
0.5
0.0
f1 (ppm)

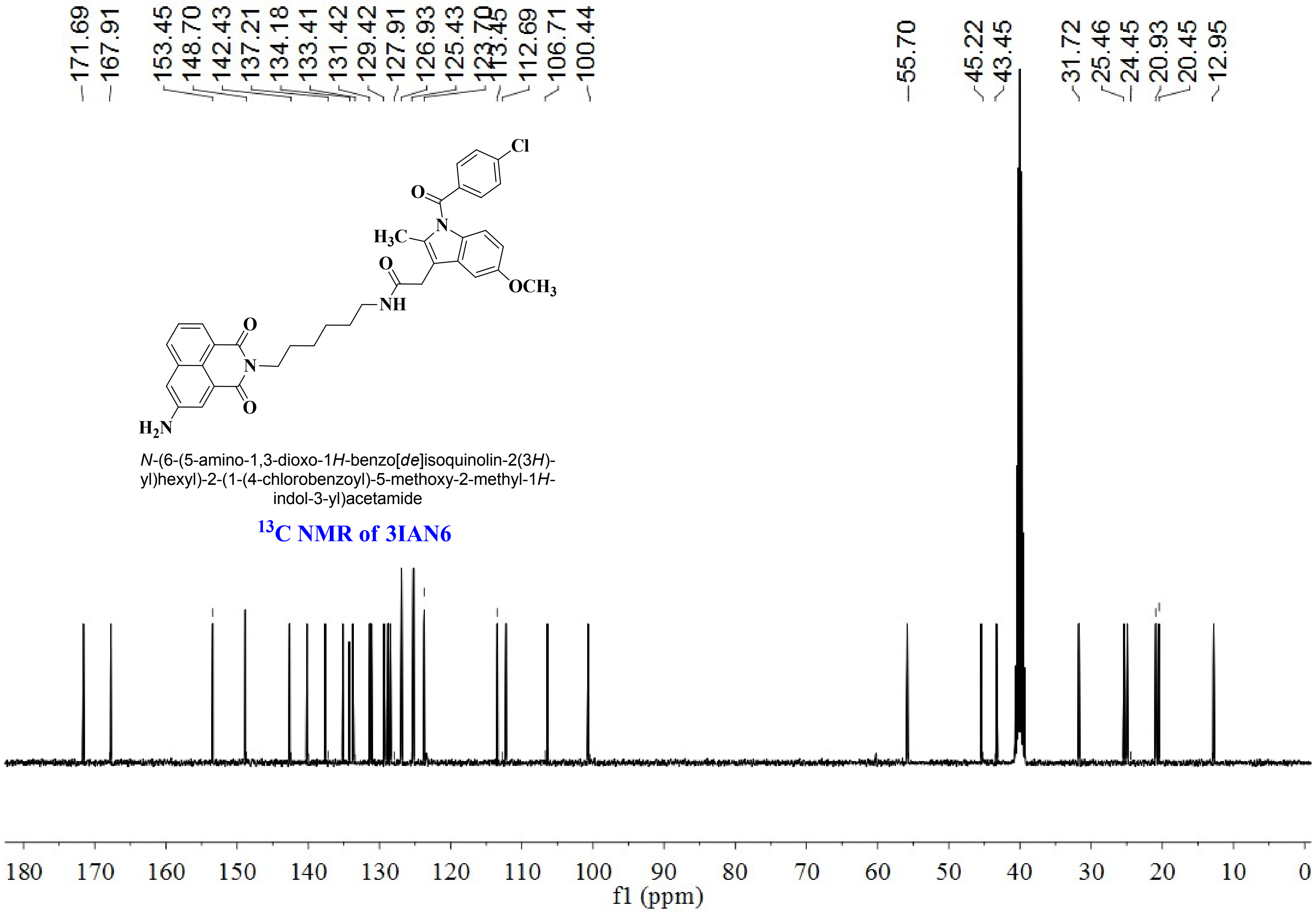

N-(6-(5-amino-1,3-dioxo-1*H*-benzo[*de*]isoquinolin-2(3*H*)-yl)hexyl)-2-(1-(4-chlorobenzoyl)-5-methoxy-2-methyl-1*H*-indol-3-yl)acetamide

^{13}C NMR of 3IAN6

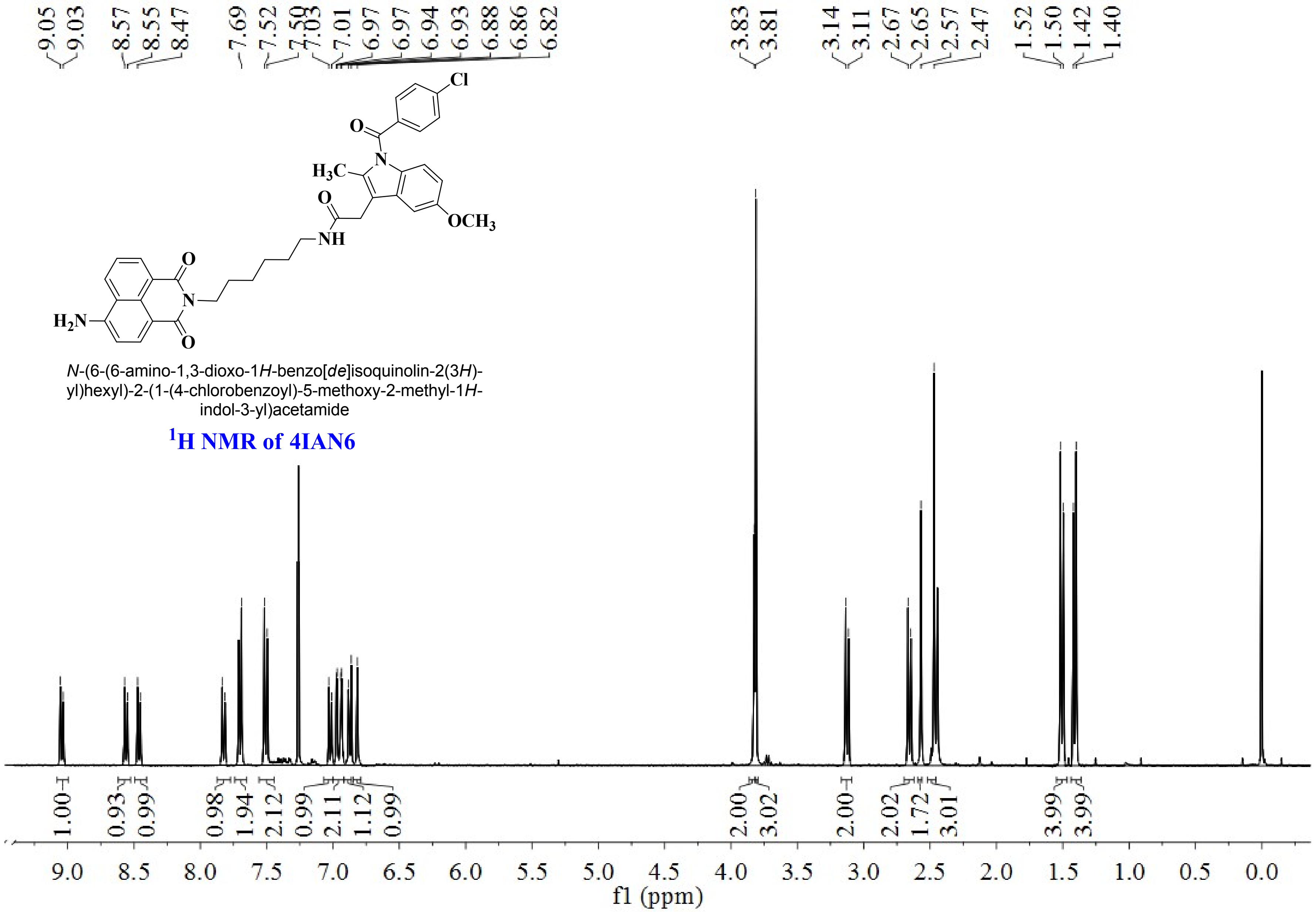

9.05
9.03
8.57
8.55
8.47
7.69
7.52
7.50
7.03
7.01
6.97
6.97
6.94
6.93
6.88
6.86
6.82
3.83
3.81
3.14
3.11
2.67
2.65
2.57
2.47
1.52
1.50
1.42
1.40
Cl
O
N
H3C
O
OCH3
NH
O
N
H2N
O
N-(6-(6-amino-1,3-dioxo-1H-benzo[de]isoquinolin-2(3H)-yl)hexyl)-2-(1-(4-chlorobenzoyl)-5-methoxy-2-methyl-1H-indol-3-yl)acetamide
1H NMR of 4IAN6
1.00
0.93
0.99
0.98
1.94
2.12
0.99
2.11
1.12
0.99
2.00
3.02
2.00
2.02
1.72
3.01
3.99
3.99
9.0
8.5
8.0
7.5
7.0
6.5
6.0
5.5
5.0
4.5
4.0
3.5
3.0
2.5
2.0
1.5
1.0
0.5
0.0
f1 (ppm)

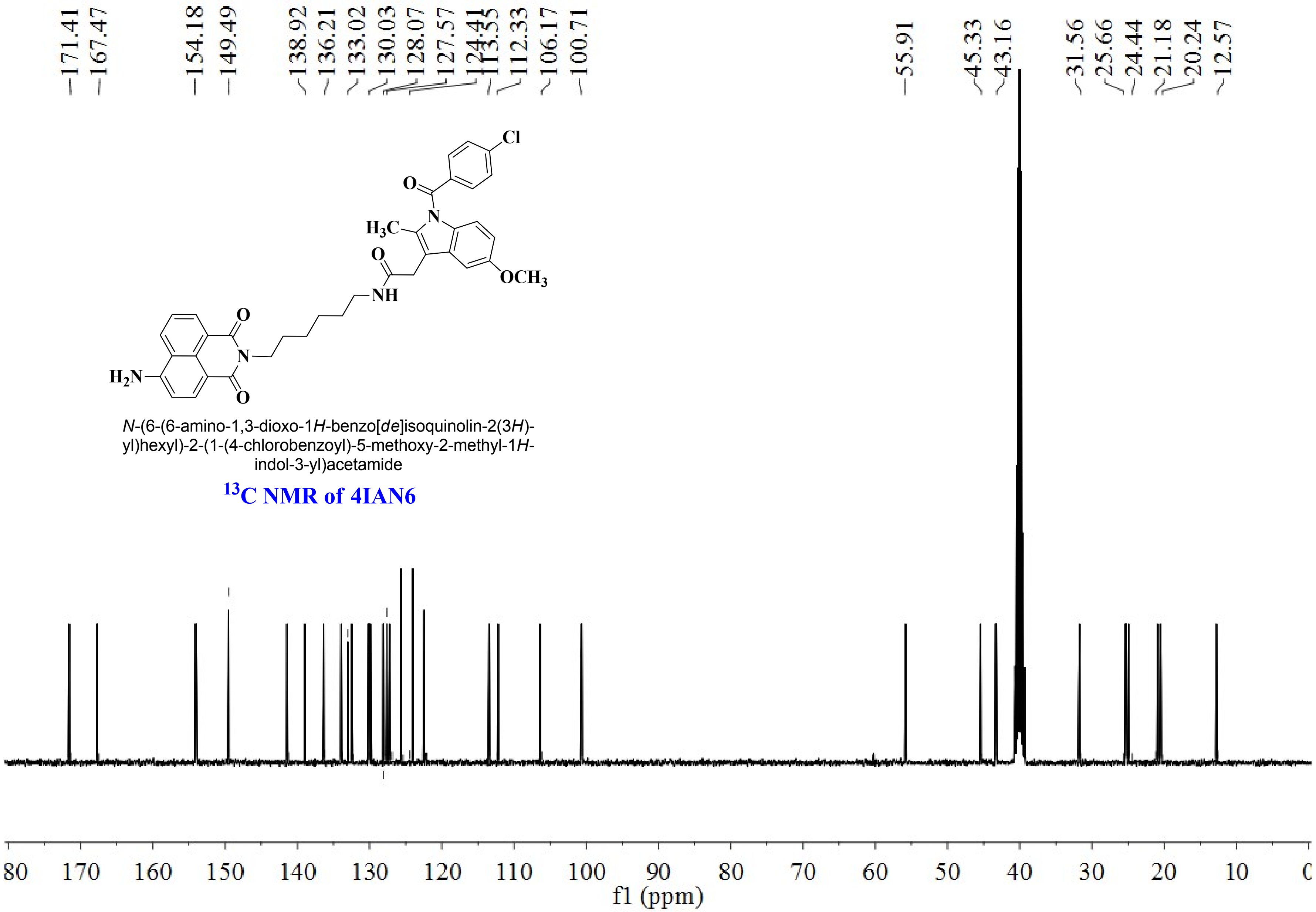

N-(6-(6-amino-1,3-dioxo-1*H*-benzo[*de*]isoquinolin-2(3*H*)-yl)hexyl)-2-(1-(4-chlorobenzoyl)-5-methoxy-2-methyl-1*H*-indol-3-yl)acetamide

^{13}C NMR of 4IAN6
